# Modelling Conformational Flexibility in a Spectrally Addressable Molecular Multi‐Qubit Model System

**DOI:** 10.1002/anie.202207947

**Published:** 2022-10-12

**Authors:** Ciarán J. Rogers, Deepak Asthana, Adam Brookfield, Alessandro Chiesa, Grigore A. Timco, David Collison, Louise S. Natrajan, Stefano Carretta, Richard E. P. Winpenny, Alice M. Bowen

**Affiliations:** ^1^ National Research Facility for Electron Paramagnetic Resonance Spectroscopy Department of Chemistry and Photon Science Institute The University of Manchester Oxford Road Manchester M13 9PL UK; ^2^ Department of Chemistry Ashoka University Sonipat Haryana India; ^3^ Dipartimento di Scienze Matematiche Fisiche e Informatiche Università di Parma 43124 Parma Italy; ^4^ INFN–Sezione di Milano-Bicocca Gruppo Collegato di Parma I-43124 Parma Italy; ^5^ UdR Parma INSTM I-43124 Parma Italy

**Keywords:** Heterometallic Complexes, Molecular Magnetism, Multi-Spin, Pulsed Dipolar Spectroscopy, Quantum Information Processing

## Abstract

Dipolar coupled multi‐spin systems have the potential to be used as molecular qubits. Herein we report the synthesis of a molecular multi‐qubit model system with three individually addressable, weakly interacting, spin 1/2 centres of differing *g*‐values. We use pulsed Electron Paramagnetic Resonance (EPR) techniques to characterise and separately address the individual electron spin qubits; Cu^II^, Cr_7_Ni ring and a nitroxide, to determine the strength of the inter‐qubit dipolar interaction. Orientation selective Relaxation‐Induced Dipolar Modulation Enhancement (os‐RIDME) detecting across the Cu^II^ spectrum revealed a strongly correlated Cu^II^‐Cr_7_Ni ring relationship; detecting on the nitroxide resonance measured both the nitroxide and Cu^II^ or nitroxide and Cr_7_Ni ring correlations, with switchability of the interaction based on differing relaxation dynamics, indicating a handle for implementing EPR‐based quantum information processing (QIP) algorithms.

## Graphical abstract

Systems with weak dipolar interactions have the potential to be used for molecular quantum computing. Orientation selective pulsed EPR was used to model the conformational flexibility of a molecular multi‐qubit model system. Spectrally addressable centres allowed for the individual manipulation of each spin qubit, while differing relaxation dynamics resulted in a proposed handle for the implementation of quantum algorithms.

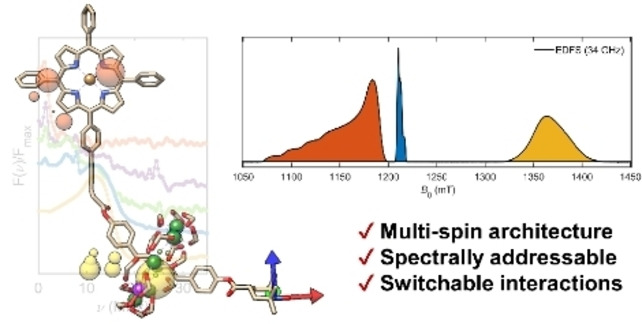

Molecular electron spin qubits are promising candidates for use in scalable Quantum Information Processing (QIP) algorithms.[^1^, ^2^, ^3^, ^4^, ^5^] One[^6^, ^7^] and two[^8^, ^9^, ^10^] qubit systems have been reported, with the latter facilitating the implementation of many quantum logic gates, including the CNOT and CPHASE gates,[^11^, ^12^] while factorisation of 21 has been achieved on an exchanged coupled three qubit system.^[13]^ To this end, the synthesis of complex nanomolecular architectures containing heterospins of different *g*‐values, allowing spectral addressability, has been reported recently by our group,^[14]^ and others,[^15^, ^16^] and has also been shown in an analogous organic biradical system with rigid geometry in an aligned crystal matrix to facilitate the operation of a CNOT gate.^[17]^ Further, molecular electron spins in orthogonal qubit pairs have been shown not to contribute to qubit decoherence, and offer a handle for enhanced qubit control through multi‐frequency spin addressability.^[18]^ The coherent manipulation of entangled qubit interactions between electron spin pairs in a >2 qubit system will thus be essential for implementing more exotic quantum algorithms, such as quantum error correction, or quantum simulation of decoherence.[^19^, ^20^, ^21^] An important consideration for the design of multi‐qubit molecular systems is the extent of flexibility along the semi‐rigid organic linkers joining the qubits, determining the strength and spread of the inter‐qubit dipolar magnetic interactions.

Pulsed Electron Paramagnetic Resonance (EPR) methods can be exploited for coherent manipulation of the quantum sublevels of molecular spin qubit systems,[^22^, ^23^] and through Pulsed Dipolar Spectroscopy (PDS), give access to the distribution of distance between two or more dipolar coupled spin centres.^[24]^ Additionally PDS may allow the relative orientation between spin centres to be determined, identifying geometric constraints on conformational structure in low‐order samples.[^25^, ^26^, ^27^, ^28^] While >2 spin architectures have previously been explored purely for the determination of inter‐spin interactions, or number of coupled spins,[^29^, ^30^, ^31^] they have not yet been exploited for EPR‐based QIP applications, which will rely on precise knowledge of the inter‐qubit interactions, and further, the extent of multi‐spin effects (MSEs) on the fidelity of qubit pair interactions. Anti‐ferromagnetic Cr_7_Ni ring systems have long been proposed as suitable qubits for QIP,[^32^, ^33^, ^34^] and offer a chemically straightforward way to bring together, in a controlled manner, entangled arrays of molecular spin qubits.[^35^, ^36^, ^37^] To demonstrate this, we employ a cross‐coupling reaction in the synthesis of a model three spin 1/2 system containing individually addressable, dissimilar spin centres (full details in Supporting Information, S.1).

Complex (**1**), (Figure 1) is a hybrid [2]‐rotaxane comprised of a Cu^II^ porphyrin covalently linked to one end of a nitroxide capped cationic ammonium thread, around which a Cr_7_Ni ring has been templated using established methods.^[38]^ Continuous Wave (CW) and Echo Detected Field Sweep (EDFS) EPR measurements confirmed the presence of three spin moieties in the multi‐spin complex (**1**) and were collected at Q‐ and X‐band (Figure 2a–c). Samples for CW analysis were prepared to a final concentration of 1 mM (toluene:THF:CHCl_3_/1 : 1 : 1). Spin Hamiltonian parameters were determined via simulation of the experimental spectra with exchange coupling = 0 cm^−1^, indicating a system of well separated Cu^II^, Cr_7_Ni ring, and nitroxide spin centres, where the magnetic inter‐qubit interactions are expected to be purely dipolar. Samples of (**1**) for pulsed EPR experiments were prepared to a final concentration of ca. 200 μM (anhydrous d8‐toluene:d8‐THF:CDCl_3_/1 : 1 : 1). Longitudinal (*T*_1_) relaxation times (Q‐band, 3 K) follow the order nitroxide>Cu^II^≫Cr_7_Ni ring, with *T*_1_ values of 94 ms, 37 ms and 0.024 ms, respectively.

**Figure 1:**
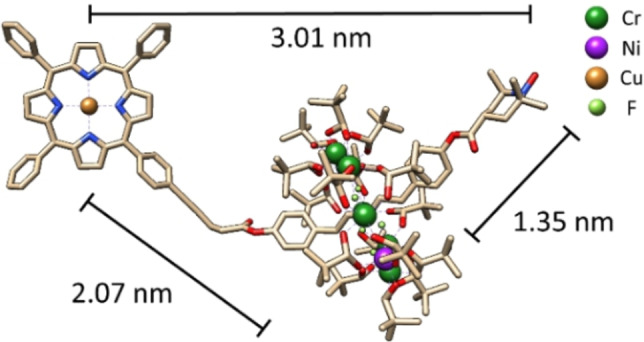
DFT optimised model (see Supporting Information, S.4) of the single crystal XRD (X‐Ray Diffraction) structure of complex (**1**), a multi‐spin Cu^II^ porphyrin‐Cr_7_Ni ring‐nitroxide hybrid [2]‐rotaxane (H atoms have been removed for clarity).

**Figure 2:**
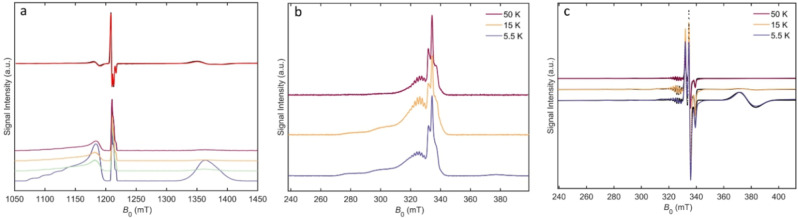
a) Q‐band (34 GHz) Continuous Wave (CW) spectrum of compound (**1**) at 5 K (black) and simulated spectrum (red). Q‐band (34 GHz) variable temperature Echo Detected Field Sweep (EDFS) spectra of (**1**) at 3 K (blue), 4 K (green), 5 K (yellow), 6 K (violet). From low to high field, the resonant spins are Cu^II^, nitroxide, Cr_7_Ni ring. b) X‐band (9.31 GHz) EDFS spectra of (**1**). c) Experimental (coloured) and simulated (black) X‐band (9.38 GHz) CW spectra of (**1**). (Simulation parameters available in Supporting Information, Table S.3.1).

This difference in *T*_1_ times primes the multi‐qubit system for Relaxation‐Induced Dipolar Modulation Enhancement (RIDME) experiments (Figure 3a).^[39]^ RIDME has been developed in systems[^40^, ^41^, ^42^, ^43^] where a detection sequence on a slow relaxing observer spin is modulated by stochastic spin‐flips of a faster relaxing spin during an optimised mixing block time, *T*_mix_. Generally *T*_mix_ is optimally 5 times *T*_1_ of the fast relaxing spin,^[44]^ although good results have been recorded with 0.7 times *T*_1_ of the fast relaxing spin.^[45]^ RIDME has an advantage over Double Electron‐Electron Resonance (DEER) as the method is not limited by the bandwidth of a pump pulse or the achievable detection – pump frequency offset. However, addressability of only one spin centre in RIDME experiments remains a limitation of this pulse sequence for use in EPR‐based QIP algorithms, and would necessitate a spectrometer pulsing at two different resonance frequencies. For compound (**1**) at 3 K, detecting on the Cu^II^ spin, while choosing a *T*_mix_ within which the Cr_7_Ni ring can sufficiently relax, large orientation selectivity is expected due to the anisotropy of the Cu^II^ spectrum which spans ≈ 3 GHz at Q‐band.^[46]^ This interaction is expected to be purely pairwise as a result of both spectral separation and the fact that the nitroxide spin has only relaxed to 0.12 % of its *T*_1_ value within the time of *T*_mix_ (120 μs). In this case, (**1**) can be treated as a two‐spin system, as MSEs, which would be detrimental to the fidelity of any molecular based quantum operation, are expected to be minimal.^[47]^

**Figure 3:**
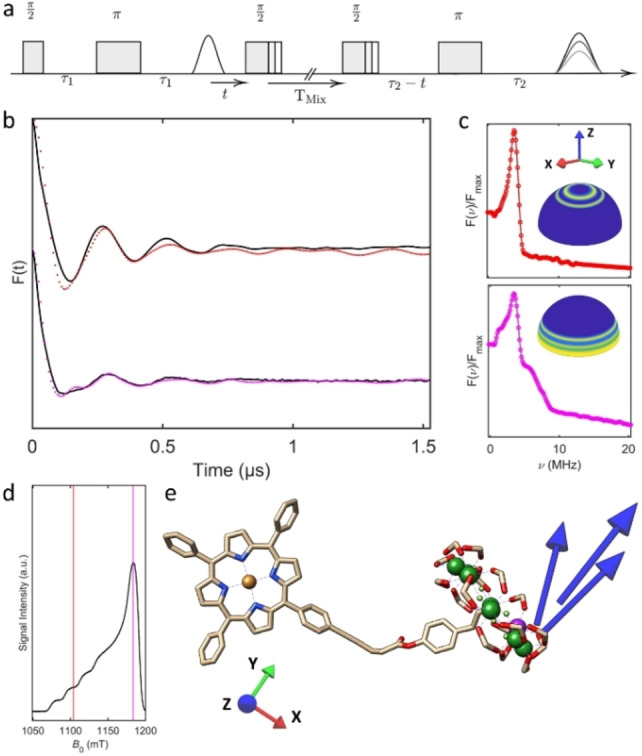
a) Dead‐time free five pulse RIDME sequence.^[39]^ b) Phased and background‐corrected os‐RIDME Form factors (black) and corresponding best fitting simulations at field positions centred at *g_z_*, 1104 mT (red) and *g_xy_*, 1183.5 mT (magenta). c) Fourier transform of experimental RIDME time‐domain data (coloured as above). (Inset) Spherical intensity maps indicating the excited orientational sub‐populations for the Cu^II^ centre with respect to *B*_0_ (blue ‐ low excitation, yellow ‐ high excitation). d) EDFS spectrum (34 GHz) of the Cu^II^ resonance with the corresponding detection sequence field positions shown. e) DFT optimised structure of (**1**) in the detection frame of the Cu^II^ spin, showing the relative orientation of the *g*_z_ component (blue arrows) of the Cr_7_Ni ring, with the base of the arrow at the predicted centre of the fitted ring orientation, determined from the best fitting simulations (top 1/3 of fits). The nitroxide fragment, H atoms and *tert*‐butyl residues have been removed.

For RIDME measurements at Q‐band (34 GHz, 3 K) a five‐pulse dead‐time free sequence^[39]^ was employed and the Cu^II^ observer sequence was positioned at both the high field, *g_xy_*, and the low field, *g_z_*, regions. Selective excitation of the *g_xy_* position yields strong orientation selectivity, resulting in higher relative intensity of the higher frequency contributions to the trace (Figure 3b), and partial Pake pattern (Figure 3c). For the measurement recorded on the Cu^II^ *g_z_* position, the higher frequency components, corresponding to the “wings” or *z*‐orientation of a complete Pake pattern, are suppressed. A modified version of the orientation dependent simulation algorithm reported by Lovett et al.,^[25]^ was used to model the inter‐spin interactions of (**1**), and is described in detail (see Supporting Information, S.5). An iterative least squares fitting routine, as reported previously,^[48]^ identifies the best simulated fits to the experimental time domain data, thereby determining the most probable distance distributions and relative orientations of the corresponding *g*‐matrices in space. The presence of several oscillations in the experimental time traces indicate that a limited range of dipolar interactions and thus conformational distributions are present for these two centres, with the predicted relative orientation of the Cr_7_Ni ring *g*_z_ component, in the frame of the Cu^II^ *g*‐tensor, corroborating single‐crystal studies of Cr_7_Ni ring *g*‐tensor orientation^[49]^ and supporting restricted molecular flexibility between these spin centres. The predicted Cr_7_Ni ring *g*_z_ component of the top 1/3 of fits are plotted relative to the DFT optimised model (Figure 3e). Slightly longer inter‐qubit distances (2.16–2.18 nm) are obtained from the best fitting simulations when compared to the DFT model (2.07 nm).

RIDME measurements at Q‐band (34 GHz) detecting on the nitroxide spin, with *T*_mix_ optimised at 3 K for isolating the nitroxide‐Cr_7_Ni ring interaction resulted in a much faster dipolar oscillation due to the shorter through space spin‐spin distance of ca. 1.33 nm; this is consistent with the single crystal XRD data (1.35 nm) of the two‐spin intermediate complex (crystal structure available in Supporting Information, S.2). This pairwise interaction could be confidently isolated up to temperatures of 5 K and for a *T*_mix_ on the order of 1 ms. As *T*_mix_ is increased, relaxation of the Cu^II^ spin becomes sufficient to also modulate the detection spin echo. It is assumed that the dipolar oscillations seen at 5 K with variable *T*_mix_ are dominated by the individual pairwise interactions rather than by MSEs, therefore best fitting simulations of the pairwise interactions at 3 K and 6 K were simply scaled and summed based on the % of Cu^II^ *T*_1_ that is accessible within the chosen *T*_mix_ in order to fit the data (further details in Supporting Information, S.5). The best fitting centres of spin density at both 3 K (yellow) and 6 K (orange) are shown relative to the DFT model (Figure 4d), demonstrating switchable addressability at 6 K to the Cu^II^‐nitroxide interaction. At temperatures greater than 6 K, the dominant *S*=1/2 ground state of the Cr_7_Ni ring gives way to the population of fast relaxing excited states,^[50]^ enabling isolation and spectral addressability of the Cu^II^‐nitroxide dipolar interaction at higher temperatures by DEER (15 K and 50 K os‐DEER data available in Supporting Information, S.3). A self‐consistent model containing a global multi‐frequency fit of orientation parameters determined from both DEER and RIDME data gives an indication of the restricted conformational flexibility of the molecule relative to a fixed position of the Cr_7_Ni ring (Figure 4c).

**Figure 4:**
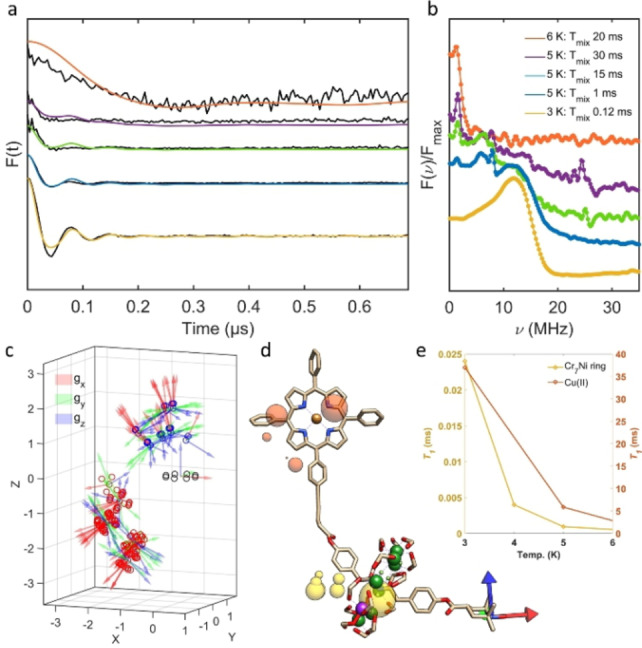
a) Variable temperature RIDME detecting on the nitroxide maximum field position (1210.4 mT) at 34 GHz with varying *T*_mix_ and temperature. Phased and background corrected RIDME Form factors (black) with corresponding orientation dependent best fitting simulations (coloured). b) Fourier transform of experimental RIDME time‐domain data showing the spread and strength of the dipolar interaction as temperature and *T*_mix_ are varied. c) Geometric plot of best fitting simulated *g*‐tensor orientations of Cu^II^ (red circles) and nitroxide (blue circles) from a self‐consistent global fit of the dipolar data in the *g*‐frame of the Cr_7_Ni ring (black circles) d) Centre of spin densities of best fitting RIDME traces to (**1**) at 3 K (yellow spheres) and 6 K (orange spheres), relative to the nitroxide *g*‐tensor (*g_x_* =red, *g_y_* =green, *g_z_* =blue). H atoms and *tert*‐butyl residues have been removed. e) Comparison of *T*_1_ relaxation values of Cr_7_Ni ring (yellow) and Cu^II^ (orange).

The key characteristics of the model multi‐qubit system, namely the weak inter‐qubit interactions and individual qubit addressability, ensuring factorised eigenstates, coupled with the disparity in *T*_1_ of the constituent molecular spins, highlights the Cr_7_Ni ring as an excellent ancillary qubit for mimicking the effect of the environment in inducing decoherence. If an entangled state of the Cr_7_Ni ring with one, or both, of the other spin qubits is generated, the comparatively fast relaxation of the Cr_7_Ni ring would lead to a controlled decoherence in the state of the others. For instance, decoherence of the two “logical” qubits can be simulated by a fast single qubit rotation of the Cr_7_Ni ring, followed by a three qubit Toffoli gate,^[51]^ i.e., a flip of the Cr_7_Ni ring spin conditioned by the state of the “logical” qubits, which is permitted by dipolar interactions. Upon relaxation of the Cr_7_Ni ring, the state of the other spin qubits coincides with the one in which decoherence has acted for a time controlled by the angle of the initial rotation of the Cr_7_Ni ring spin (further details in Supporting Information, S.6). Of particular interest is the study of decoherence on an entangled state of the Cu^II^ and nitroxide spin qubits. The two‐qubit gate operation times are defined by the strength of the interaction between the qubits. In this system the two‐qubit gate operation times range from 10s to 100s of ns, which is favourable for the range of decoherence times for this system (ranging from 100s to 1000s of ns) at cryogenic temperatures, and the achievable single gate operation times, defined by the pulse lengths. The inherent limitation of pulsing at multiple resonance frequencies to achieve the necessary entangled states may be realised using circuit quantum electrodynamic (QED) architectures, as applied recently to Yb(trensal) molecular spin qubits.^[52]^ The weak dipolar interactions in the multi‐qubit model system necessitate long pulses to implement the corresponding gate operations, however optimal control pulse shaping techniques may circumvent this by reducing the probability of unwanted transitions, even with fast pulses.^[53]^ Moreover, stronger dipolar interactions obtained by chemically controlling the inter‐qubit distance would not be detrimental to such a protocol as a result of the individually addressable *g*‐values of each spin qubit. Finally, synthetic approaches to enhance the coherence time on the Cr_7_Ni spin while preserving fast *T*_1_ relaxation have been reported,^[54]^ increasing the fidelity of the required quantum gate operations.

To summarise, we have synthesised and measured coherent, switchable, and spectrally addressable dipolar interactions in a molecular multi‐qubit model system. Highly orientation selective Cu^II^‐Cr_7_Ni ring RIDME interactions have been measured for the first time, and the dipolar data could be modelled to give an indication of global structural flexibility in the complex. Further, variable temperature RIDME experiments detecting on the nitroxide resonance could be selectively tuned to target either the Cu^II^‐nitroxide or nitroxide‐Cr_7_Ni ring interaction, conferring individual spin centre addressability based on differing spin relaxation properties. The results presented suggest that disparity in spin relaxation dynamics can be exploited to mitigate detrimental issues of MSEs in the measurement of pairwise inter‐qubit magnetic interactions, and further, may allow us to probe the controlled decoherence of entangled qubit pairs via the fast longitudinal relaxation of an ancillary spin qubit. These results therefore aim to guide development in frequency addressable molecular multi‐qubit candidates for future quantum computation and simulation routines.

## Conflict of interest

The authors declare no conflict of interest.

## Supporting information

As a service to our authors and readers, this journal provides supporting information supplied by the authors. Such materials are peer reviewed and may be re‐organized for online delivery, but are not copy‐edited or typeset. Technical support issues arising from supporting information (other than missing files) should be addressed to the authors.

Supporting Information

## Data Availability

The data that support the findings of this study are openly available in Github at https://github.com/ciaranrogers16/MATLAB‐scripts‐for‐EPR.git.
