# Supplementary material for: Modelling Conformational Flexibility in a Spectrally Addressable Molecular Multi‐Qubit Model System
Source: Angew Chem Int Ed Engl. 2022 Oct 12;61(45):e202207947. doi: 10.1002/anie.202207947 (PMC9828767; doi:10.1002/anie.202207947)
Supplement: Supplementary file 1 — Supporting Information [file ANIE-61-0-s001.pdf]

Angewandte Chemie
Eine Zeitschrift der Gesellschaft Deutscher Chemiker

Supporting Information

Modelling Conformational Flexibility in a Spectrally Addressable Molecular Multi-Qubit Model System

C. J. Rogers, D. Asthana, A. Brookfield, A. Chiesa, G. A. Timco, D. Collison, L. S. Natrajan, S. Carretta, R. E. P. Winpenny, A. M. Bowen**

Table of Contents

S.1 - Experimental procedures 3
S.2 - Crystallographic data 7
S.3 - EPR measurements 9
S.4 - Construction of model structures for orientation dependent simulations 16
S.5 - Orientation dependent dipolar simulations 32
S.6 - EPR-based Quantum Simulation of Decoherence 39
S.7 - Spectroscopic data 41
References 50

S.1 - Experimental procedures

All reagents and solvents were purchased from Sigma-Aldrich and/or Alfa and used without further purification. Column chromatography was performed using either Machery Nagel 0.063 - 0.2 mm silica gel from Sigma-Aldrich, or a Grace Reverelis ® X2 Autocolumn with Grace Reverelis ® NP cartridges. Reactions were monitored by thin layer chromatography (TLC) on Alugram Xtra SIL G / UV 254 silica gel. ^{1}H NMR spectra were obtained on a Bruker Avance III HD 400 MHz instrument equipped with a BBFO inverse probe and were carried out in $CDCl_3$. Chemical shifts are reported in parts per million (ppm) from low to high frequency and referenced to the residual solvent resonance. UV-vis spectra were recorded on a Mettler Toledo UV5Bio spectrophotometer using 10 mm path length quartz cuvettes in spectroscopic grade solvents. Where the molar extinction coefficient was not calculated explicitly sample concentrations no higher than ~10^{-6} M were used and the data were consistent with previously reported results.[2b] Mass spectrometry and microanalysis experiments were carried out by the services at The University of Manchester.

Synthetic procedures

The synthesis of the mono-amine functionalised free base porphyrin **(2)** was according to the procedure of A.M. Bowen et al.[1] The mono-nitro derivative **(1)** was not isolated and the crude product, containing a mixture of unreacted H_2TPP and the desired mono functionalised compound (**1**), as observed by TLC, was carried through to the following step as only the functionalised compound will be reduced by the excess of $SnCl_2.2H_2O$ **(ii)**. Unreacted H_2TPP was then easily separated via column chromatography from the mono-amine product **(2)**. The Sandmeyer reaction **(iii)**, adapted from that reported by C. Frixa *et al.*,[2] was employed to give the mono-iodo functionalised free base porphyrin **(3)** from the mono-amine derivative, proceeding via a mono-azide intermediate. Metalation of the free base porphyrin using Cu(II) acetate monohydrate was rudimentary. The full conversion of free base porphyrin **(3)** to Cu(II) porphyrin **(4)** was monitored *via* the characteristic porphyrin Q-bands of the UV-vis spectrum, which undergo a change in both position and number due to the altered electronic structure, and hence symmetry, upon binding of Cu(II).

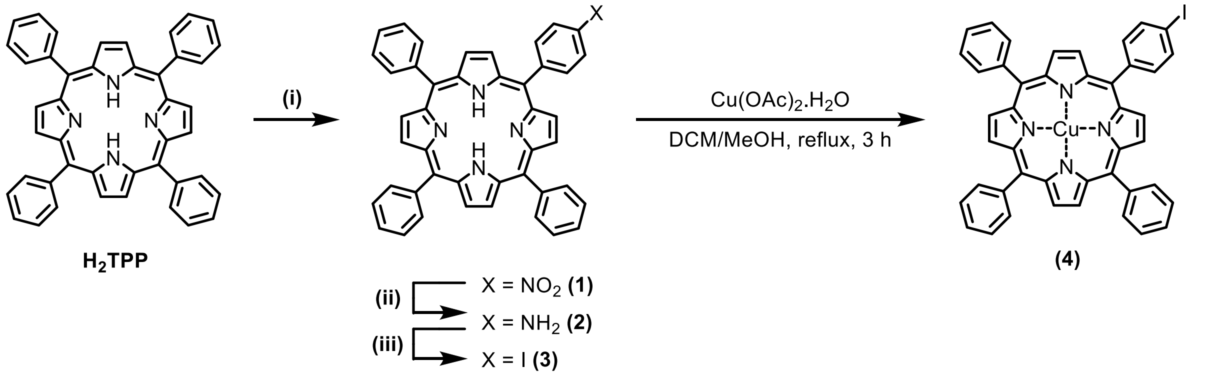

Scheme S.1.1: Synthetic route for the formation of mono-iodo functionalised copper(II) porphyrin (**4**). *Reagents/Conditions:* **(i)** $NaNO_2$, TFA, 10°C, 3 mins **(ii)** $SnCl_2.2H_2O$, conc. HCl, 65°C, 2 h **(iii)** ***a.*** $NaNO_2$, H_2SO_4, H_2O, 0°C, 10 mins ***b.*** KI, H_2O, 40°C, 1h.

The synthesis of the hybrid [2]-rotaxane, CuTPP-{Cr_7Ni}-TEMPO **(9)**, follows the general procedure reported previously by Winpenny and co-workers for the synthesis of anionic Cr_7Ni rings templated around a cationic ammonium thread.[3] Simple nucleophilic substitution under basic conditions **(i)** gives access to the bi-phenol thread **(5)** around which the Cr_7Ni ring is then templated **(6)**, upon mixing at high temperatures, with the appropriate chromium and nickel metal salts in an excess of pivalic acid **(ii)**.

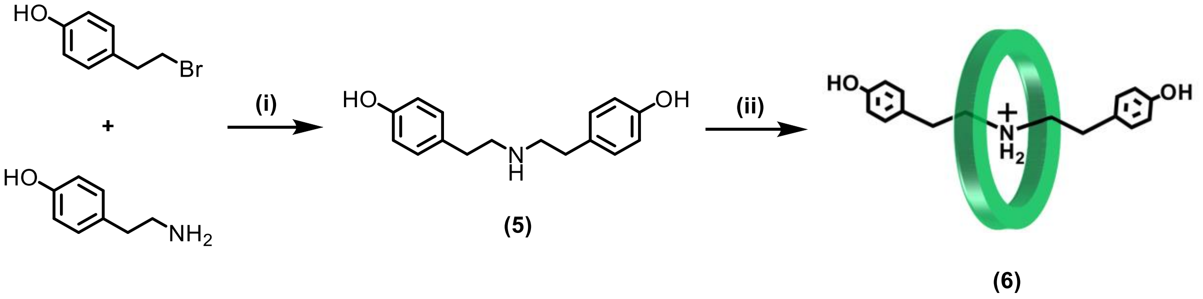

Scheme S.1.2: Synthetic route for the formation of [2]-rotaxane (**6**). *Reagents/Conditions:* **(i)** DIPEA, DMF (anhyd.), 75°C, N_2, 16 h **(ii)** $CrF_3.4H_2O$, $2NiCO_3.3Ni(OH)_2.4H_2O$, PivOH, 140°C, 24 h.

The thread of the newly formed [2]-rotaxane (**6**) is then selectively functionalised *via* an asymmetric esterification reaction under standard coupling conditions **(i)** with an alkyne bearing substituent on a single end of the thread. Once isolated, the remaining phenol substituent **(7)** is free to partake in a subsequent esterification reaction with 4-carboxy-TEMPO **(ii)** to give [2]-rotaxane-TEMPO (**8**). Sonogashira coupling **(i)** to the mono-iodo functionalised Cu(II) porphyrin (**4**) under relatively mild conditions afforded the title three spin compound, CuTPP-{Cr_7Ni}-TEMPO **(9)** in adequate yield (40%).

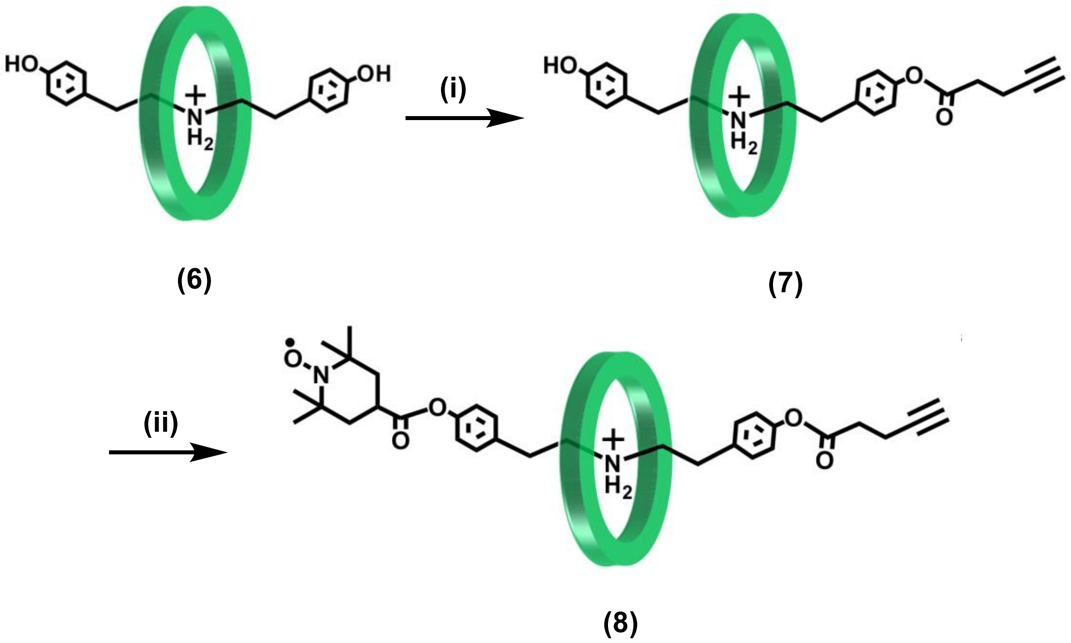

Scheme S.1.3: Synthetic route for the formation of [2]-rotaxane-TEMPO (**8**). *Reagents/Conditions:* **(i)** Pentynoic acid, DCC, DMAP, THF (anhyd.), 50°C, N_2, 16 h **(ii)** 4-Carboxy-TEMPO, DCC, DMAP, DCM (anhyd.), rt, N_2, 16 h.

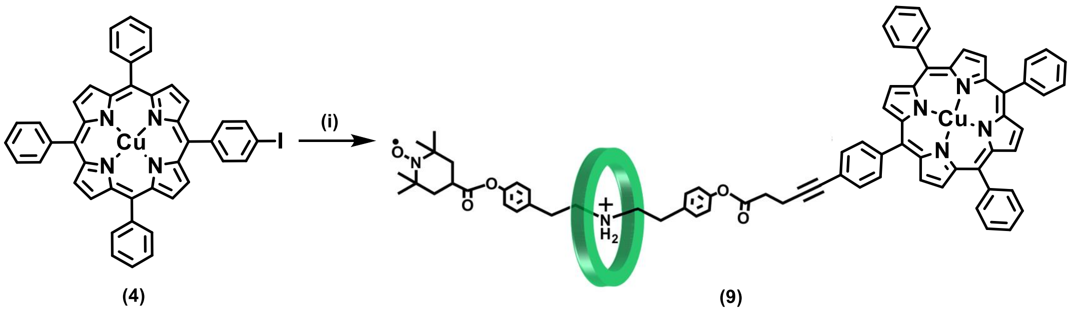

Scheme S.1.4: Synthetic route for the formation of CuTPP-{Cr_7Ni}-TEMPO **(9).** *Reagents/Conditions:* **(i)** [2]-rotaxane-TEMPO **(8),** $Pd(PPh_3)_4$, CuI (cat.), 1,4-dioxane (anhyd.), Diisopropylamine, rt-50°C, N_2, 20 h.

5-(4-nitrophenyl)-10,15,20-triphenylporphyrin - H_2TPPNO_2 (1)

$NaNO_2$ (0.24 g, 3.48 mmol) was added to a green solution of 5,10,15,20-tetraphenylporphyrin (H_2TPP) (1.12 g, 1.82 mmol) in trifluoroacetic acid (TFA) (35 mL) and was stirred at 10°C for 3 minutes. Ice-cold deionised H_2O (80 mL) was then added, followed by DCM (80 mL). The reaction mixture was then slowly poured into a solution of concentrated $NaHCO_3$ at 0℃. The aqueous phase was then extracted with DCM (3 x 100 mL) and the combined organic phases were washed with a saturated aqueous solution of $NaHCO_3$, dried on anhydrous Na_2SO_4, filtered and concentrated under vacuum to give a mix of compound **(1) -** H_2TPPNO_2, and unreacted H_2TPP, as a purple powder (~1 g) that was used in the following step without further purification. R_f = 0.45 (DCM/Hex: 1/1).

4-(10,15,20-triphenylporphyrin-5-yl)aniline - H_2TPPNH_2 (2)

$SnCl_2.2H_2O$ (6.97 g, 36.67 mmol) was added to a green suspension of crude H_2TPPNO_2 (~1 g) in conc. HCl (130 mL) at room temperature. The mixture was heated, with stirring, to 65°C for 2 h. The reaction mixture was then allowed to cool, slowly added to ice-cold deionised H_2O, and the pH adjusted to 9 using a saturated solution of NH_4OH. The aqueous phase was then extracted with DCM (3 x 100 mL) and the organic phases combined and dried on anhydrous Na_2SO_4, filtered and concentrated under vacuum. The crude product was purified by chromatography on silica gel (DCM/NEt_3: 99/1) to give compound **(2)** - H_2TPPNH_2 as a purple powder (450 mg, 40% yield – based on H_2TPP). R_f = 0.3 (DCM/NEt_3: 99/1). ^{1}H NMR (400 MHz, $CDCl_3$, 298 K) δ_H -2.83 (2H, s, pyrrole-NH), 3.97 (2H, s, Ar-NH_2), 7.01 (2H, d, J = 8 Hz, Ar-H), 7.69 (9H, m, Ar-H), 7.93 (2H, d, J = 8 Hz, Ar-H), 8.15 (6H, d, J = 8 Hz, Ar-H), 8.76 (6H, s, Ar-H), 8.88 (2H, d, J = 4 Hz, Ar-H). ESI *m/z* 630.2649 [M+H$^+$], calculated 630.2652 for [M+H$^+$]: C_{44} H_{32} N_5. UV-Vis ($CHCl_3$, nm, 293 K) λ_{max} 422 (Soret band), 517, 554, 592, 667 (Q-bands).

5-(4-iodophenyl)-10,15,20-triphenylporphyrin - H_2TPPI (3)

$NaNO_2$ (0.29 g, 4.20 mmol) in ice-cold deionised H_2O (25 mL) was added dropwise to a solution of H_2TPPNH_2 (0.45 g, 0.71 mmol) in H_2SO_4 (4.5 mL) and deionised H_2O (50 mL) at 0℃. The reaction was stirred for 10 minutes, after which KI (0.9 g, 5.42 mmol) in deionised H_2O (50 mL) was added to the diazonium salt at once and the reaction heated, with stirring, to 50℃ for 1 h, or until N_2 evolution ceased. The reaction mixture was then cooled, extracted with $CHCl_3$ and washed with an aqueous solution of Na_2CO_3, followed by an aqueous solution of Na_2SO_3. The organic phases were then washed with deionised water, combined, and dried on anhydrous Na_2SO_4, filtered and concentrated under vacuum. The crude product was purified by a plug of silica gel ($CHCl_3$, 100%) to give compound **(3)** as a dark purple powder (250 mg, 45%). R_f = 0.9 ($CHCl_3$, 100%). ^{1}H NMR (400 MHz, $CDCl_3$, 298 K) δ_H -2.88 (2H, s, pyrrole-NH), 7.69 (9H, m, Ar-H), 7.89 (2H, d, J = 8 Hz, Ar-H), 8.03 (2H, d, J = 8 Hz, Ar-H), 8.14 (6H, d, J = 8 Hz, Ar-H), 8.78 (8H, s, Ar-H). ESI *m/z* 741.1512 [M+H$^+$], calculated 741.1510 for [M+H$^+$]: C_{44} H_{30} N_4 I. UV-Vis ($CHCl_3$, nm, 293 K) λ_{max} 422 (Soret band), 516, 551, 592, 647 (Q-bands).

5-(4-iodophenyl)-10,15,20-triphenyl-21*H*,23*H*-porphine copper(II) - CuTPPI (4)

$Cu(OAc)_2.H_2O$ (10 eq., 83 mg, 0.42 mmol) was added to a solution of H_2TPPI (30 mg, 0.042 mmol) dissolved in DCM (45 mL) and MeOH (15 mL). The reaction mixture was stirred vigorously at reflux for 3 h in the dark, after which time the reaction was allowed to cool, concentrated under vacuum and dissolved in $CHCl_3$. The organic phase was washed with deionised H_2O (3 x 30 mL), dried on anhydrous Na_2SO_4, filtered and concentrated under vacuum to give compound **(4)** as a red-pink powder (29 mg, 90%). ESI *m/z* 802.0643 [M+H$^+$], calculated 802.0649 for [M+H$^+$]: C_{44} H_{28} N_4 Cu I. UV-Vis ($CHCl_3$, nm, 293 K) λ_{max} 421 (Soret band), 538 (Q-band).

4,4'-(azanediylbis(ethane-2,1-diyl))diphenol - (5)

2-(4-Hydroxyphenyl)-1-bromoethane (2.0 g, 9.95 mmol) and 2-(4-Hydroxyphenyl)ethylamine (6.0 g, 43.7 mmol) were reacted in the presence of *N*-Ethyldiisopropylamine (DIPEA) (5.2 mL) in dry DMF (45 mL). The reaction mixture was

heated at 75 °C for 16 h under an atmosphere of nitrogen. The mixture was then cooled to room temperature and extracted with ethyl acetate. The organic phase was washed with deionised water, a brine solution and then dried over anhydrous $MgSO_4$, before filtration and concentration under vacuum to obtain the crude product. The solid residue was dissolved in hot methanol (MeOH) followed by addition of deionised water (20 mL). The crystalline product was filtered and washed repeatedly with $CHCl_3$ to give compound **(5)** (1.8 g, 70%). ^1H NMR (400 MHz, DMSO-d_6, 298 K) δ_H 9.15 (2H, s, Ar-OH), 6.95 (4H, d, J = 8 Hz, Ar-H), 6.64 (4H, d, J = 8 Hz, Ar-H), 2.67 (4H, m, Ph-C*H*$_2$-CH$_2$), 2.55 (4H, m, Ph-CH$_2$C*H*$_2$), 1.43 (1H, s, NH). Elemental Analysis: calculated (found) for $C_{16}H_{19}NO_2$: **C**, 74.68 (74.03); **H**, 7.44 (7.45); **N**, 5.44 (5.45).

$[(C_{16}H_{20}NO_2)Cr_7NiF_8(C_5H_9O_2)_{16}]$ - [2]-rotaxane (6)

The procedure for the formation of the [2]-rotaxane was as previously reported, but has been briefly included here for the reader. A mixture of pivalic acid (45 g), $[2NiCO_3.3Ni(OH)_2{\cdot}4H_2O]$ (1.0 g), $CrF_3.4H_2O$ (6.0 g) and **(5)** (2.5 g) in a Teflon flask was heated at 140 °C for 24 h. The reaction mixture was then cooled to room temperature and filtered with $CHCl_3$. The crude product was purified by chromatography on silica gel ($CHCl_3$/EtOAc:7/3). ESI *m/z* 2473.63 [M+Na$^+$], calculated 2473.89 for [M+Na$^+$]: $C_{96}H_{164}Cr_7F_8NNiO_{34}Na$. Elemental Analysis: calculated (found) for $C_{96}H_{164}Cr_7F_8NNiO_{34}$: **C**, 47.04 (47.35); **H**, 6.74 (6.84); **N**, 0.57 (0.65); **Cr**, 14.85 (13.78); **Ni**, 2.39 (2.32).

$[(C_{21}H_{24}NO_3)Cr_7NiF_8(C_5H_9O_2)_{16}]$ - [2]-rotaxane (7)

[2]-rotaxane **(6)** (3.0 g, 1.2 mmol) was reacted with pentynoic acid (0.12 g, 1.2 mmol), DCC (0.9 g, 4.3 mmol) and DMAP (0.75 g, 6.1 mmol) in dry THF (100 mL). The reaction mixture was stirred under a flow of nitrogen at 50°C for 16 h. The solvent was then evaporated and the crude product purified by chromatography on silica gel ($CHCl_3$/EtOAc:97/3) to yield the mono-esterified [2]-rotaxane **(7)** (0.75 g, 25%). ESI *m/z* 2553.63 [M+Na$^+$], calculated 2554.04 for [M+Na$^+$]: $C_{101}H_{168}Cr_7F_8NNiO_{35}Na$. Elemental Analysis: calculated (found) for $C_{101}H_{168}Cr_7F_8NNiO_{35}$: **C**, 47.93 (48.11); **H**, 6.69 (6.85); **N**, 0.55 (0.61); **Cr**, 14.38 (13.58); **Ni**, 2.32 (2.30).

$[(C_{31}H_{40}N_2O_5)Cr_7NiF_8(C_5H_9O_2)_{16}]$ – [2]-rotaxane-TEMPO (8)

[2]-rotaxane **(7)** (0.75 g, 0.3 mmol) was reacted with an excess of 4-carboxy-TEMPO (0.18 g, 0.9 mmol), DCC (0.55 g, 2.6 mmol) and DMAP (0.55 g, 4.5 mmol) in dry DCM (50 mL). The reaction mixture was stirred overnight at room temperature under a flow of nitrogen. The solvent was evaporated and the crude product then purified by chromatography on silica gel ($CHCl_3$/MeOH:98/2) to yield pure [2]-rotaxane-TEMPO (**8**) (0.65 g, 80%). Green crystals suitable for single crystal X-Ray diffraction were grown via slow evaporation from acetone. ESI *m/z* 2713.7 [M+H$^+$], calculated 2713.3 for [M+H$^+$]: $C_{111}H_{185}Cr_7F_8N_2NiO_{37}$. Elemental Analysis: calculated (found) for $C_{111}H_{184}Cr_7F_8N_2NiO_{37}$: **C**, 49.14 (50.11); **H**, 6.84 (6.94); **N**, 1.03 (1.39); **Cr**, 13.41 (12.37); **Ni**, 2.16 (2.11). UV-Vis ($CHCl_3$, nm, 293 K) λ_{max} (ε / M^{-1} cm^{-1}) 430 (5625), 622 (5814).

$[C_{75}H_{66}O_5N_6CuCr_7NiF_8(C_5H_9O_2)_{16}]$ - CuTPP-{Cr_7Ni}-TEMPO (9)

A 100 mL dry round bottomed flask was vacuumed and filled with nitrogen gas to which CuTPPI **(4)** (0.030 g, 0.03 mmol), [2]-rotaxane-TEMPO **(8)** (0.085 g, 0.03 mmol), Palladium-tetrakis(triphenylphosphine) (0.020 g, 0.01 mmol), copper(I) iodide (0.050 g, 0.02 mmol) and dry 1,4-dioxane (10 mL) was added. Diisopropylamine (0.5 mL) was subsequently added and the reaction mixture allowed to stir for 4 h at room temperature under an atmosphere of nitrogen. The temperature of the reaction was then raised to 50°C and stirring was continued for 16 h. Completion of the reaction was monitored by TLC. The crude product was purified by chromatography on silica gel ($CHCl_3$/EtOAc:97/3) to give pure CuTPP-{Cr_7Ni}-TEMPO **(9)** as a ruby red powder (0.04 g, 40%). ESI *m/z* 3404.935 [M+NH$_4^+$], calculated 3405.63 for [M+NH$_4^+$]: $C_{155}H_{210}Cr_7CuF_8N_6NiO_{37}$. MALDI-TOF-MS *m/z* 3404.4 [M+NH$_4^+$], M.W. = 3387.60 g/mol. Elemental Analysis: calculated (found) for $C_{155}H_{210}Cr_7CuF_8N_6NiO_{37}$: **C**, 54.96 (54.88); **H**, 6.25 (6.21); **N**, 2.48 (2.43); **Cr**, 10.74 (10.31); **Ni**, 1.73 (1.74); **Cu**, 1.88 (1.82). UV-Vis (toluene:THF:$CHCl_3$/1:1:1, nm, 293 K) λ_{max} (ε / M^{-1} cm^{-1}) 419 (160,000), 541 (13,630). CW EPR data reported in ***Table S.3.1***.

S.2 - Crystallographic data

Single crystal X-Ray diffraction data for **[2]-rotaxane-TEMPO (8)** was collected at a temperature of 100 K using a dual wavelength Rigaku FR-X with Cu-Kα radiation equipped with a HypixHE6000 detector and an Oxford Cryosystems nitrogen flow gas system. Data was measured using GDA and CrysAlisPro suite of programs. Single crystal XRD structures were visualised in Mercury software. The full details for this structure is available in pre-print.[12] Attempts to crystallise (**9**) were unsuccessful in a variety of solvent mixtures and by various crystallisation methods.

Crystal structure determinations and refinements

Single crystal X-Ray diffraction (XRD) data were processed and reduced using CrysAlisPro suite of programmes. Absorption correction was performed using empirical methods (SCALE3 ABSPACK) based upon symmetry-equivalent reflections combined with measurements at different azimuthal angles.[4] The crystal structure was solved and refined against all F^2 values using the SHELXL and Olex 2 suite of programmes.[5] All atoms in crystal structures were refined anisotropically with the exception of the hydrogen atoms that were placed in the calculated idealized positions for all crystal structures. The pivalate and dimethylacrylate ligands, and threads in crystal structures were disordered and modelled over two positions, using structural same distance (SADI) and distance fix (DFIX) Shelxl restraints commands. The atomic displacement parameters (adp) of the ligands have been restrained using similar Ueq and rigid bond (SIMU) and Similar Ueq (SIMU) restraints.

Table S.2.1: Crystallographic data for [2]-rotaxane-TEMPO **(8).**

Identification code	[2]-rotaxane-TEMPO (8)
Empirical formula	$C_{120} H_{202} Cr_7 F_8 N_2 Ni O_{40}$
Formula weight	2887.53
Temperature/K	100.00(10)
Crystal system	monoclinic
Space group	C2/c
a/Å	58.8525(10)
b/Å	16.5562(3)
c/Å	31.1637(4)
α/°	90
β/°	92.731(2)
γ/°	90
Volume/Å^3	30330.6(8)
Z	8
ρ_{calc}g/cm^3	1.265
μ/mm^{-1}	4.781
F(000)	12192.0
Crystal size/mm^3	0.55 × 0.43 × 0.33
Radiation	Cu Kα (λ = 1.54184)
2Θ range for data collection/°	3.006 to 152.486
Index ranges	-74 ≤ h ≤ 71, -7 ≤ k ≤ 20, -39 ≤ l ≤ 39
Reflections collected	85389
Independent reflections	30626 [R_{int} = 0.0454, R_{sigma} = 0.0593]
Data/restraints/parameters	30626/532/1796
Goodness-of-fit on F^2	1.068
Final R indexes [I>=2σ (I)]	R_1 = 0.0772, wR_2 = 0.2216
Final R indexes [all data]	R_1 = 0.0949, wR_2 = 0.2371
Largest diff. peak/hole / e Å^{-3}	1.21/-0.87

Crystal structure of [2]-rotaxane-TEMPO (8)

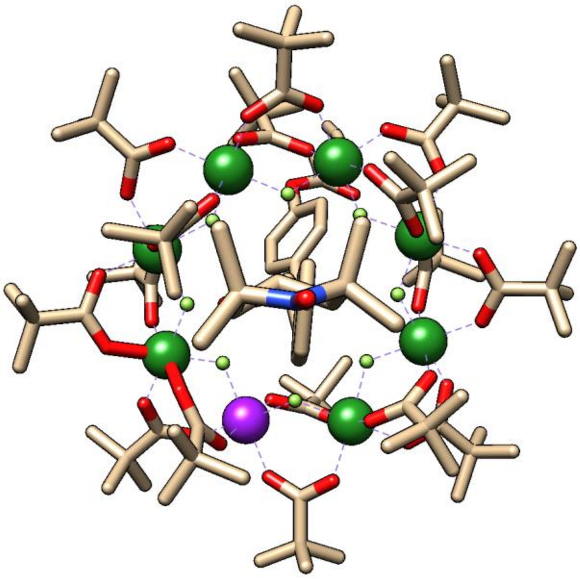

Figure S.2.1.a: Single crystal XRD structure of [2]-rotaxane-TEMPO **(8)** – head-on ring view**.**

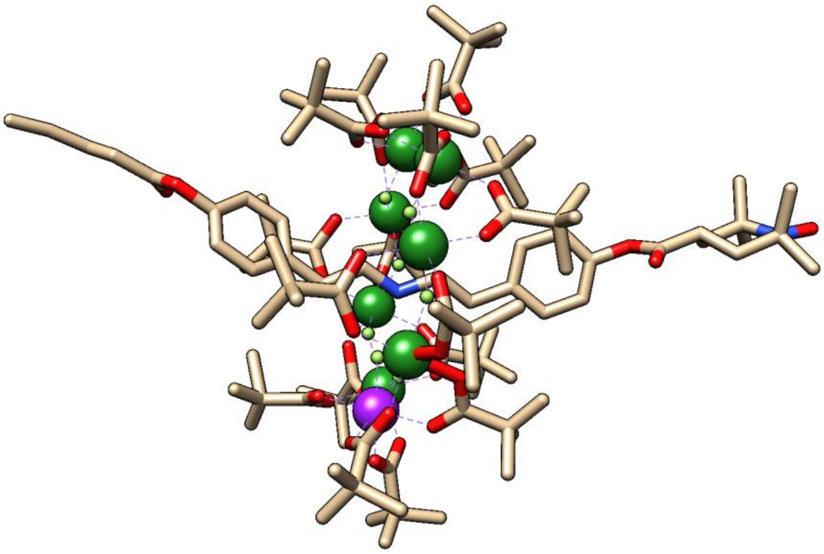

Figure S.2.1.b: Single crystal XRD structure of [2]-rotaxane-TEMPO **(8)** – side-on ring view**.**

S.3 - EPR measurements

Samples of CuTPP-{Cr_7Ni}-TEMPO **(9)** for pulsed EPR were prepared to a final concentration of ca. 200 µM in a 1:1:1 anhydrous mixture of d8-toluene:d8-THF:$CDCl_3$ in 3 mm and 5 mm (o.d.) quartz tubes. Continuous Wave (CW) samples were prepared to a concentration of 1 mM in a 1:1:1 mixture of toluene:THF:$CHCl_3$. Anhydrous deuterated solvents were obtained from Sigma-Aldrich as sealed ampoules and used as purchased, or purged with nitrogen (BOC) and passed through columns containing activated alumina and molecular sieves. The samples were degassed by three freeze-pump-thaw cycles, flame sealed, flash-frozen and then stored in liquid nitrogen. Pulsed EPR data were collected at Q-band on a Bruker ELEXSYS 580 FT spectrometer using a 3 mm Dual Mode Resonator (T2), and at X-band on a Bruker ELEXSYS E580X FT spectrometer using a 5 mm Split-Ring Resonator (MS5). Low temperature measurements were collected using a Cryogenic cryogen free variable temperature cryostat, or an Oxford Instruments cryostat incorporating a closed helium circuit. All pulses were amplified via a pulsed travelling wave tube (TWT) amplifier. Spectral simulations and analysis of spectra were performed in MATLAB R2020a, using *Easyspin* 5.2.30 simulation software[6] and were phase and background corrected to obtain Form factors before Fourier transformation to obtain a Pake pattern. For orientation independent data analysis, Tikhonov Regularization using the DeerAnalysis2019 routine was used to obtain the corresponding distance distributions. Orientation dependent simulations were carried out using a modified version of the algorithm reported by Lovett *et al*.[7] (*Full details and GitHub repository link in S.5*).

Continuous wave EPR simulation parameters

Table S.3.1: Spin system parameters of the individual spins for CuTPP-{Cr_7Ni}-TEMPO **(9)**, determined by simulation of the Q-band (34 GHz) CW EPR spectrum at 5 K using the *Easyspin* routine '*pepper'* implented in MATLAB. The simulation parameters below were used to input spin system data for the orientation dependent simulations detailed in S.5. The spin Hamiltonian parameters obtained from fitting the simulation to the experimental data are in line with those reported for the corresponding component spin centres; Cr_7Ni ring,[8] nitroxide,[9] Cu(II) porphyrin.[10]

Simulation Parameter	Cr_7Ni ring (g_x, g_y, g_z)	Nitroxide (g_x, g_y, g_z)	Cu(II) (g_x, g_y, g_z)
g-matrix	1.74, 1.78, 1.80	2.0019, 2.0059, 2.0096	2.05, 2.05, 2.20
g-strain	0.0054, 0.0050, 0.0032	-	-
H-strain (MHz)	-	-	27, 27, 366
^{14}N - A (MHz)	-	100, 23, 21	21, 21, 77
^{63,65}Cu - A (MHz)	-	-	6, 6, -806
lwpp (mT)	14	1.5	5.84, 1.06

Variable temperature X-band (9.39 GHz) CW measuerements of CuTPP-{Cr_7Ni}-TEMPO (9)

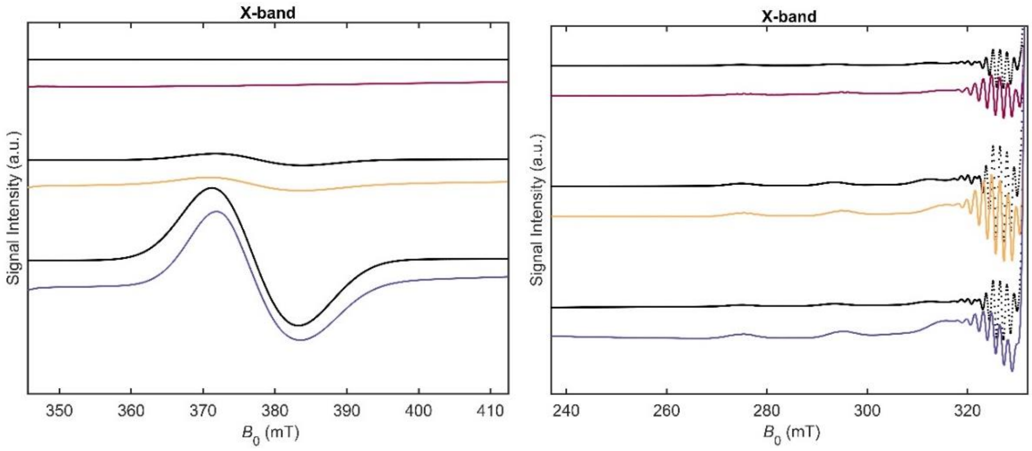

Figure S.3.1: *Left*: Variable temperature X-band (9.39 GHz) CW data of compound (**9**), experimental (black) at 50 K (violet), 15 K (yellow) and 5.5 K (blue), highlighting the Cr_7Ni ring region, simulated using the *Easyspin* routine 'pepper'. *Right:* The same, highlighted from 240 mT to 335 mT.

Spin-lattice relaxation measurements (T_1) of (9) at Q-band (34 GHz) - 3 K

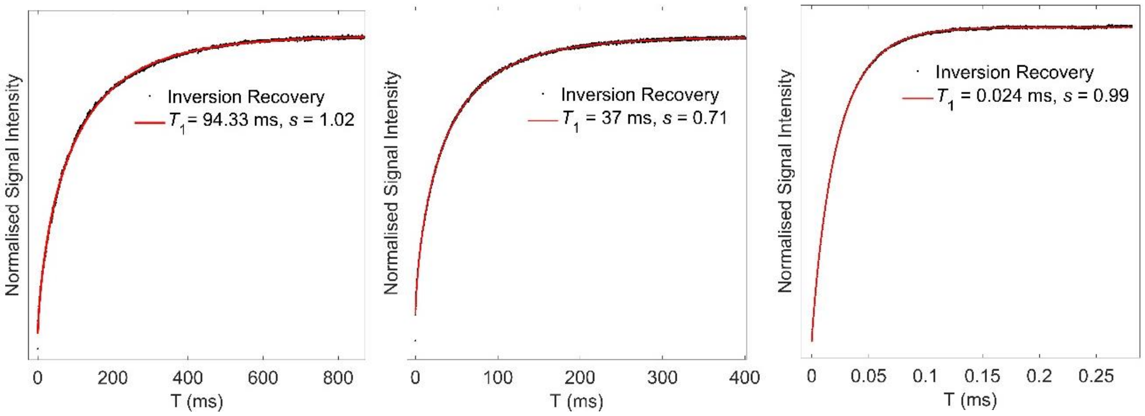

Figure S.3.2: (*Left*) Inversion recovery (T_1) measured at nitroxide field position 1213.2 mT (black). (*Middle*) T_1 measured at Cu(II) field position 1182.4 mT (black). (*Right*) T_1 measured at Cr_7Ni ring field position 1364.2 mT. All T_1 measurements were fit to a stretched exponential decay with a stretching factor *s*, in order to determine T_1 values (*red*). Pulse lengths of 12/24 ns and tau = 400 ns were used with a delay time of 1 μs between the detection pulses and the inversion pulse.

Spin-spin relaxation measurements (T_2) of (9) at Q-band (34 GHz) - 3 K

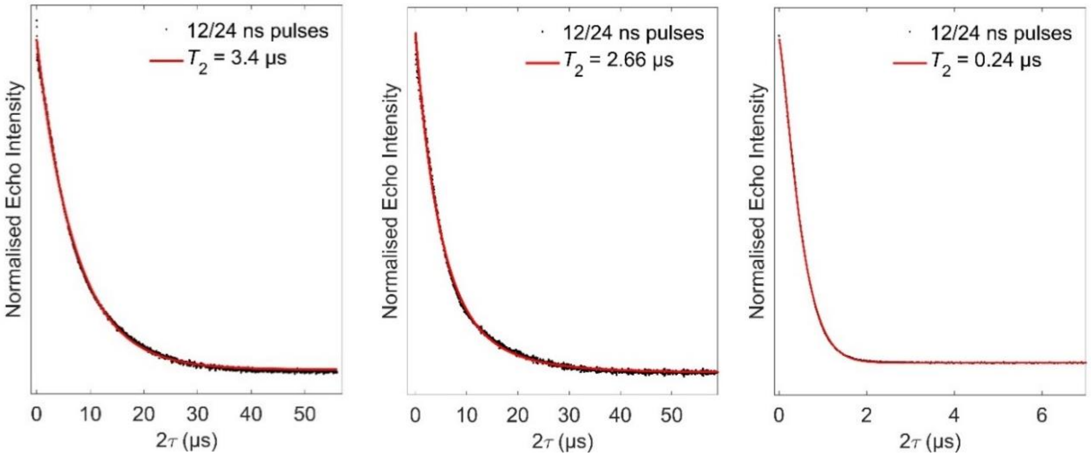

Figure S.3.3: (*Left*) T_2 measured at nitroxide field position 1213.2 mT, fit to a simple mono-exponential decay (red). (*Middle*) T_2 measured at Cu(II) field position 1182.4 mT, fit to a bi-exponential decay (red). (*Right*) T_2 measured at Cr_7Ni ring field position 1364.2 mT, fit to a stretched exponential decay (red).

Variable temperature spin-lattice relaxation measurements (T_1) of (9) at Q-band (34 GHz) detecting on the Cr_7Ni ring (Field position = 1364.2 mT)

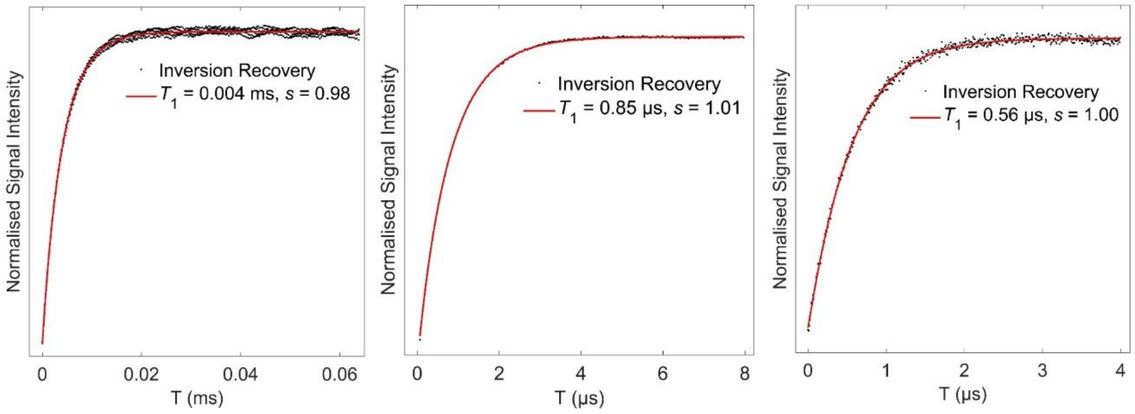

Figure S.3.4: (*Left*) T_1 measured at 4 K, fit to a stretched exponential decay (red). (*Middle*) T_1 measured at 5 K, fit to a stretched exponential decay (red). (*Right*) T_1 measured at 6 K, fit to a stretched exponential decay (red).

Variable temperature spin-lattice relaxation measurements (T_1) of (9) at Q-band (34 GHz) detecting on Cu(II) (Field position = 1183.5 mT)

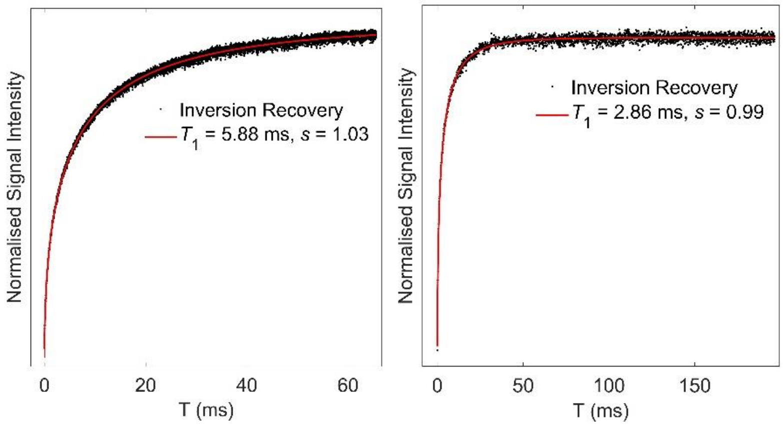

Figure S.3.5: (*Left*) T_1 measured at 5 K, fit to a stretched exponential decay (red). (*Right*) T_1 measured at 6 K, fit to a stretched exponential decay (red).

RIDME experimental details at Q-band

Table S.3.2: Experimental RIDME parameters for measurements at 3 K (Q-band) as presented in the main text.

Bruker E580Q (T2 - Dual Mode Cavity) - 3 mm quartz tube

CuTPP-{Cr7Ni}-TEMPO (9) (34 GHz)	Cu(II) - Cr_7Ni	Cu(II) - Cr_7Ni	Nitroxide - Cr_7Ni	Nitroxide - Cr_7Ni	Nitroxide - Cr_7Ni
**Magnetic Field – Det. (mT)**	1104	1183.5	1210.4	1213.2	1216.6
**Det. π/2 Pulse Length (ns)**	12	12	12	12	12
**Det. π Pulse Length (ns)**	24	24	24	24	24
**Tau 2 (ns)**	2500	2500	800	1000	800
**Number of Scans**	64	64	32	64	32
**Temperature (K)**	3	3	3	3	3
**T_{mix} (μs)**	120	120	120	120	120
**Background T_{mix} (μs)**	10	10	10	10	10
**Time step tau averaging (ns)**	16	16	32	32	32
**Number of taus to average**	8	8	4	4	4

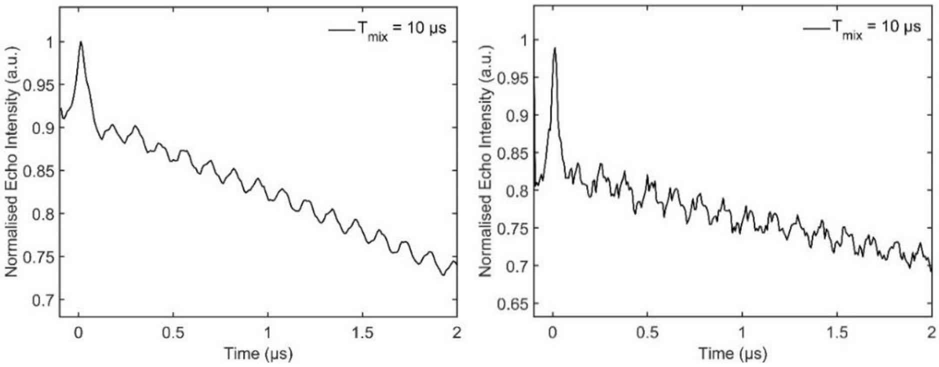

Figure S.3.6: (*Left*) Tau averaged background RIDME trace with T_{mix} = 10 μs (equal to less than half the T_1 of the Cr_7Ni ring, containing only nitrogen and deuterium ESEEM contributions) for Cu_{xy} – Cr_7Ni RIDME, with the detection field centred at 1183.5 mT. The 10 μs tau-averaged background trace was subsequently divided out from the Cu_{xy} – Cr_7Ni RIDME at T_{mix} =120 μs to give the ESEEM-suppressed RIDME trace in the main text. (*Right*) The non-tau averaged RIDME trace with T_{mix} = 10 μs. A background measurement with the same T_{mix} block of 10 μs was obtained for each field position measured.

The supplementary orientation selective RIDME data are presented as raw traces and in the experimental time and frequency domains after division of a background trace (T_{mix} = 10 μs), phasing and background correction, and for **Fig S.3.7,** the distance domain achieved after Tikhonov regularization – as this is dependent on *g*-values the obtained distance distributions are purely to visualise the suggested distance distribution between spin centres, and further, may contain artefacts owing to orientation selectivity between the centres, even after averaging.

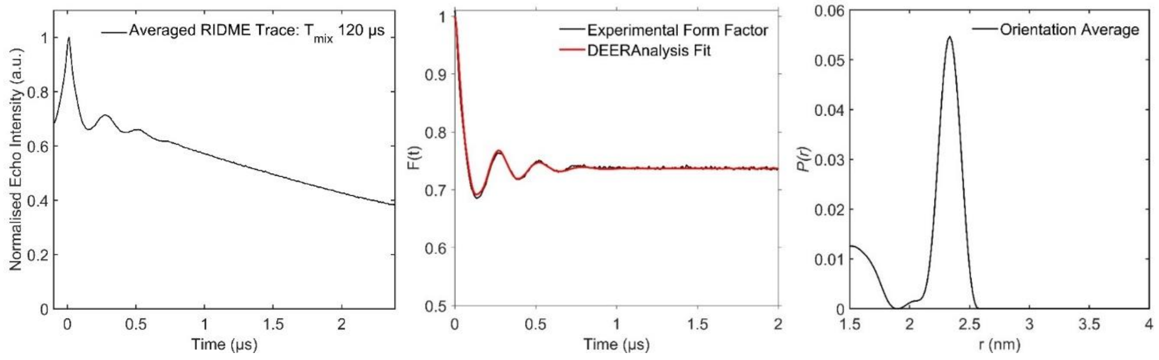

Figure S.3.7: Suppression of orientation selection in tau-averaged RIDME data for CuTPP-{Cr_7Ni}-TEMPO (**9**) at 3 K, with the RIDME detection sequence at the Cu(II) g_{xy} and the g_z positions as shown in the main text. The orientation selection was averaged by taking the sum of both RIDME traces at the aforementioned field positions (1183.5 mT and 1104 mT) and averaging. (*Top left*) Orientation averaged RIDME trace with a T_{mix} of 120 μs. (*Top right*) Form factor (black) and orientation independent DEERAnalysis fit (red). (*Bottom*) Distance distribution obtained after Tikhonov regularisation.

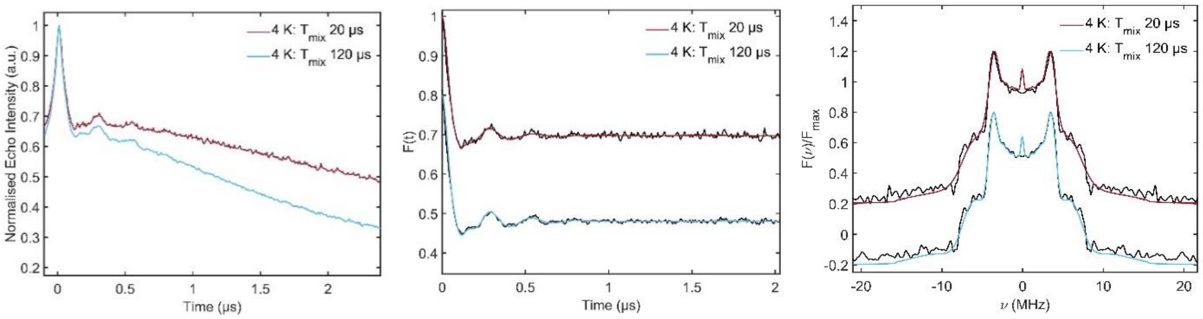

Figure S.3.8: (*Left*) Raw experimental RIDME traces detecting at 1183.5 mT (34 GHz) on the Cu_{xy} position at 4 K. Form Factor (black) and DEERAnalysis fit (coloured) for varying T_{mix} block times in non-tau averaged Cu_{xy} – Cr_7Ni RIDME, with detection field at 1183.5 mT, where the optimal T_{mix} time was taken as 5 x T_1 of the Cr_7Ni ring at 4 K (20 μs) (red) and 3 K (120 μs) (blue). (*Right*) Experimental frequency domain spectra (black) with DEERAnalysis fits (coloured)

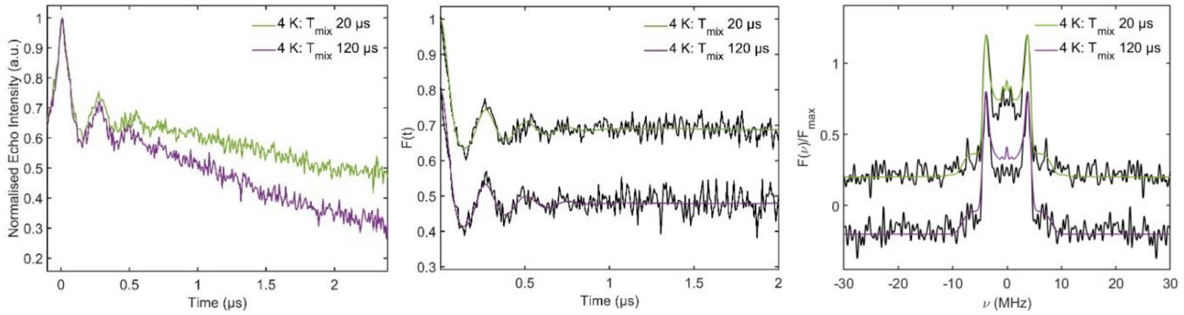

Figure S.3.9 (*Left*) Raw RIDME trace detecting at 1104.0 mT (34 GHz) on the Cu_z position at 4 K. Form factor (black) and DEERAnalysis fit (coloured) for varying T_{mix} block times in non-tau averaged Cu_z – Cr_7Ni RIDME, with detection field at 1104.0 mT, where the optimal T_{mix} time was taken as 5 x T_1 of the Cr_7Ni ring at 4 K (20 μs) (green) and 3 K (120 μs) (purple). (*Right*) Experimental frequency domain spectra.

VT-RIDME Cu(II)-Cr_7Ni (34 GHz)

Variable temperature RIDME detecting on the Cu(II) spin gave Cu(II)-Cr_7Ni RIDME signals up to 4 K, after which the traces become dominated by Electron Spin Envelope Echo Modulation (ESEEM) contributions, even with a sacrificial background trace.

DEER experimental details at X-band

Table S.3.3: Experimental DEER parameters for measurements at 15 K (X-band) as presented in the main text and below.

Bruker E580X (MS5 resonator) - 5 mm quartz tube (o.d.)

CuTPP-{Cr7Ni}-TEMPO (9)	4 pulse	4 pulse	3 pulse	3 pulse	3 pulse
**Offset (MHz)**	[-126]	[-160]	[-160]	[-100, -160]	[-300, -425, -550]
**Det. Freq. (GHz)**	[9.763]	[9.47]	[9.47]	[9.41, 9.47]	[9.40, 9.525, 9.65]
**Pump Freq. - (GHz)**	[9.637]	[9.31]	[9.31]	[9.31, 9.31]	[9.10, 9.10, 9.10]
**Det. field (mT)**	345.95	331.9	334.4	331.9	322.0
**Pump Pulse (ns)**	20	20	20	20	20
**Det. π/2 Pulse (ns)**	40	26	26	26	26
**Det. π Pulse (ns)**	40	52	52	52	52
**Tau 2 (ns)**	2000	1500	1500	1500	1500
**Temperature (K)**	15	15	15	15	15
**Time step tau averaging (ns)**	56	56	56	56	56
**Number of taus to average**	8	8	8	8	8

Three pulse DEER

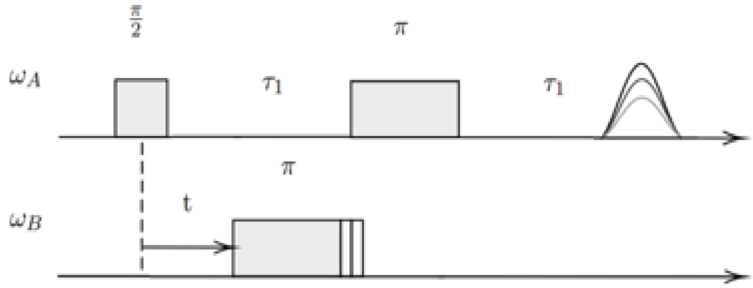

Figure S.3.10: The three pulse DEER sequence used to collect the orientation selective Cu(II)-nitroxide dipolar data at 15 K and 50 K.

The three pulse DEER sequence presented in the main text was chosen to afford greater sensitivity due to reduced transverse relaxation, however suffers from a dead-time around T = 0 of ca. 50 ns due to a temporal overlap of pump and detection pulses. When the oscillation of the dipolar interaction is expected to be large this dead-time becomes negligible. To verify this assumption, a dead-time free four pulse DEER sequence was measured under the same experimental conditions and no appreciable difference, beyond a more accurately defined modulation depth, was found between the spectra. Further, measurements pumping on the maximum of the nitroxide resonance showed little to no orientation selectivity. Three pulse orientation selective DEER (os-DEER) measurements were performed (X-band, 15 K and 50 K) to investigate the Cu(II)-nitroxide dipolar interaction.

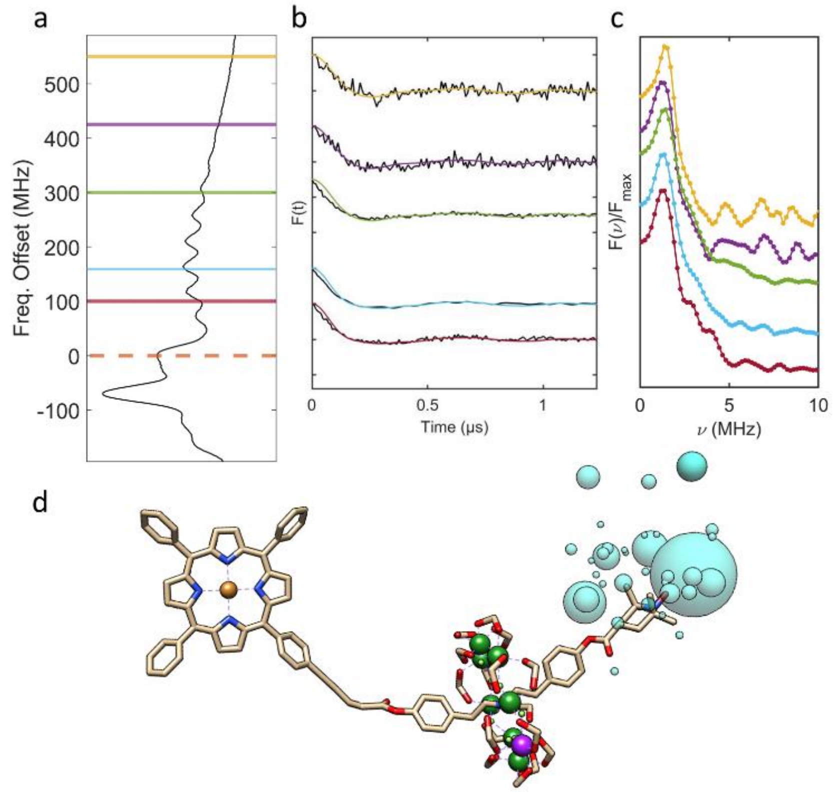

Figure S.3.11. a) EDFS spectra (black) plotted as the frequency offset of the pump frequency (orange, dashed) from the detection frequencies. ***b)*** Phased and background corrected and os-DEER Form factors of (**1**) (black) and corresponding simulated best fits at 15 K at offsets of 100 MHz (red), 160 MHz (blue), 300 MHz (green), 425 MHz (purple), 550 MHz (yellow) (*50 K os-DEER measurements available in SI).* ***c)*** Fourier transform of experimental os-DEER time-domain data (coloured as above). ***d)*** DFT optimised structure of (**1**) showing the relative centres of nitroxide spin density determined from the best fitting simulated traces (turquoise spheres, scaled by the relative number of contributions the orientation makes to the final fits). H atoms and *tert*-butyl residues have been removed.

Trends in the orientation selectivity lead to a narrower distribution in the dipolar interactions as the frequency offset between the pump and detection pulses is increased *(*Fig. S.3.11., a-c), however bandwidth limitations preclude fully sampling the g_z component of the Cu(II) spectrum and isolating it from other orientations. In all cases, the dipolar interactions are less well-defined than for the Cu(II)-Cr_7Ni interactions, as previously reported on a Cu(II) porphyrin-nitroxide model system at X-band.[11] The relative positions of simulated nitroxide spin density is plotted relative to the DFT model as turquoise spheres, scaled to represent the number of contributions to the final fit, and suggests little spread in the strength of the dipolar interaction and a higher level of flexibility between the two centres (Fig. S.3.11, d).

Cu(II)-nitroxide DEER at 15 K (X-band)

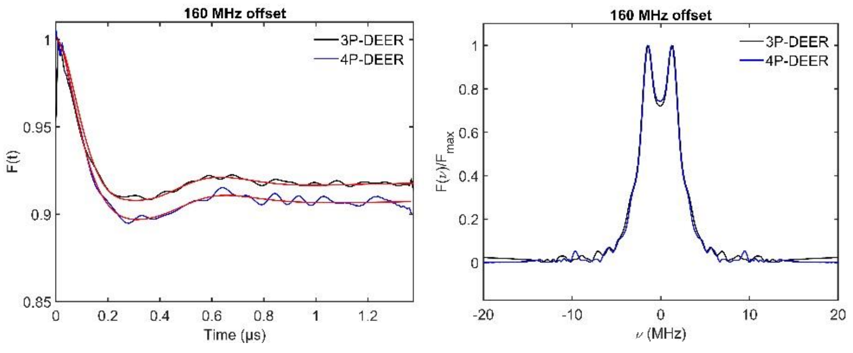

Figure S.3.12: (*Left*) 3P-DEER Form factor (black) and DEERAnalysis fit (red) vs 4P-DEER Form Factor (blue) and DEERAnalysis fit (red) at 15 K detecting on Cu(II), with the pump pulse offset by -160 MHz, under the same experimental conditions. (*Right*) Experimental dipolar trace (Fourier transform of the phased and background corrected Form factors).

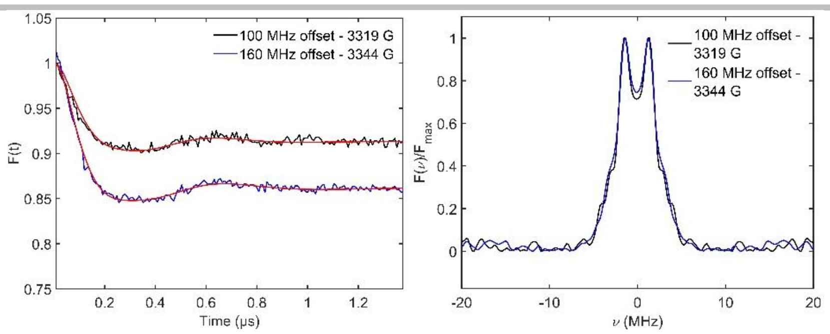

Figure S.3.13: (*Left*) 3P-DEER Form factor (black) and DEERAnalysis fit (red) for pumping on nitroxide second max (3319 G) vs 3P-DEER Form factor (blue) and DEERAnalysis fit (red) for pumping on nitroxide max (3344 G). at 15 K detecting on Cu(II), under the same experimental conditions. (*Right*) Experimental dipolar trace (Fourier transform of the phased and background corrected Form factors).

Cu(II)-NO RIDME at 50 K (Q-band)

To complement the Cu(II)-nitroxide os-DEER data presented in both the main text (15 K) and the supplementary information (50 K), os-RIDME at 50 K was performed detecting across the nitroxide spectrum. At 50 K the Cr_7Ni ring loses its well defined spin ½ ground state and as such is effectively EPR silent – thus relaxation of the ring does not need to be taken into consideration upon analysis of high temperature RIDME. These results are consistent with the data obtained from the 3P-DEER experiment and further demonstrate minimal orientation selectivity across the nitroxide spectrum, suggesting that there is a higher level of flexibility between these two spins and hence a higher number of conformational distributions between the Cu(II) spin centre and the nitroxide spin, as seen in the orientational analysis of the os-DEER experiments.

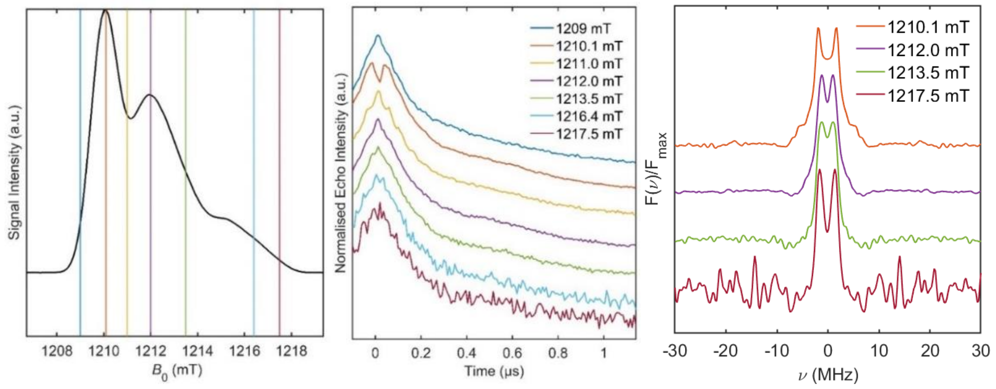

Figure S.3.14: (*Left*) EDFS of (**9**) at Q-band, 34 GHz (50 K) with coloured lines corresponding to os-RIDME field positions of the detection sequence across the nitroxide spectrum. (Right) Experimental os-RIDME traces at Q-band, 34 GHz (50 K) after division of a background RIDME trace of T_{mix} = 5 µs. Optimised T_{mix} time based on Cu(II) T_1 relaxation at 50 K = 5 x T_1 = 125 µs. (*Right*) Experimental frequency domain spectra of some representative field positions (coloured).

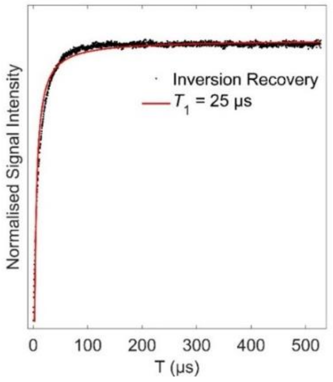

Figure S.3.15: Inversion recovery measured at Cu(II) field position 1182.4 mT (black) fit to a simple monoexponential decay (red), at Q-band, 34 GHz (50 K)

S.4 - Construction of model structures for orientation dependent simulations

Modifications of the single crystal X-ray diffraction (XRD) structure of the [2]-rotaxane-TEMPO **(8)** were carried out in Chemcraft (V.1.8) to build a structural model of the title three spin compound (**9**). DFT (Density Functional Theory) calculations were carried out in ORCA (V.4.2.1)[12] *in vacuo* to obtain optimised geometries of model fragments of the three spin system to determine both the overall confirmation structure of the compound and the orientation of the *g*-matrix of the individual spin centres with respect to the molecular frame, and relative electron spin density information, necessary for the simulation of orientation dependent dipolar spectra. The *g*-matrix orientations were aligned and plotted using Avogadro (V.1.2.0). DFT results suggested accessible energy minima at the freezing point of the solvent at ca. 120°, 170° and 20° for dihedral angles A (blue), B (orange) and C (purple) respectively – the input model for the orientation simulation was allowed to vary around these angles in order to generate the cones depicted in *S.5.1.* The DFT optimised single crystal XRD structure was presented in the main text was guided by these results, particularly the accessible orientations of dihedral A (blue).

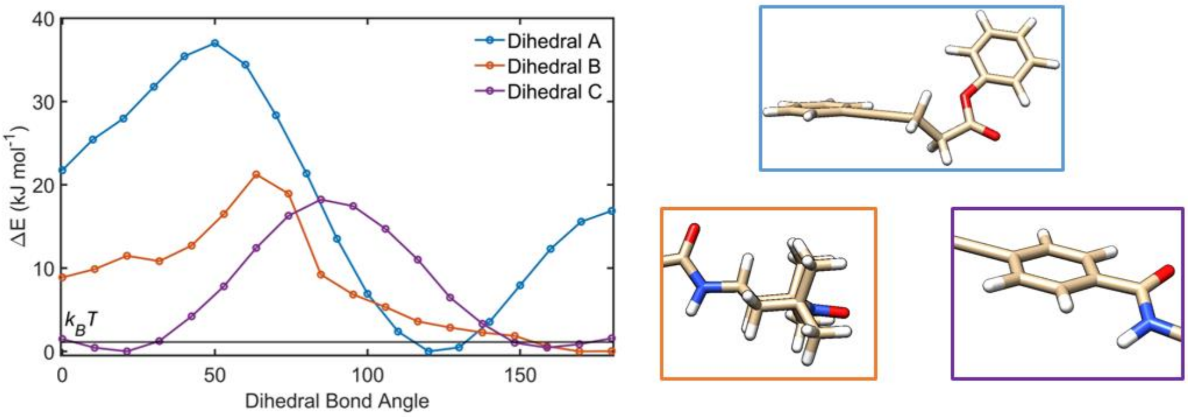

Figure S.4.1: DFT derived dihedral bond angle energies of complex **(9)** with the k_BT value indicating the range of confirmations accessible at the freezing point of the solvent mixture (1:1:1/d8THF:d8Tol:$CDCl_3$). The inset coloured boxes represent the lowest energy confirmations of the individual model fragments from the DFT results.

Orientation of the Cu(II) porphyrin *g*-matrix with respect to the molecular frame

The DFT geometry optimised structure of the Cu(II) porphyrin fragment of complex (**9**) was used to compute the EPR parameters necessary for orientation dependent simulations. The axial *g*-matrix of Cu(II) porphyrins has been well reported,[13] and this was confirmed by DFT calculations implemented in ORCA using the BP functional in combination with basis sets def2-SVP, and def2-TZVP on the Cu(II) atom, an RI approximation, and an empirical dispersion correction to the energies. The g_z component of the axial *g*-matrix is aligned perpendicular to the plane of the porphyrin ring, while the g_x=g_y components lie in the plane of the porphyrin ring.

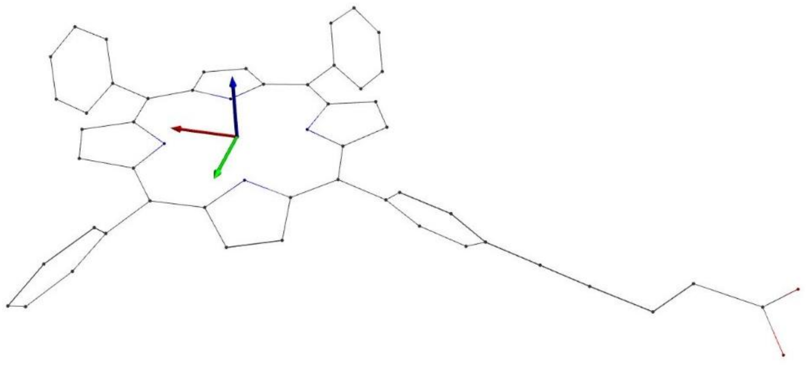

Figure S.4.2: Orientation of the *g*-matrix in the molecular frame of the Cu(II) porphyrin fragment of the title compound (**9**), with the xyz matrix coordinates corresponding, in order, to the RGB colour sequence. Hydrogen atoms have been removed for clarity.

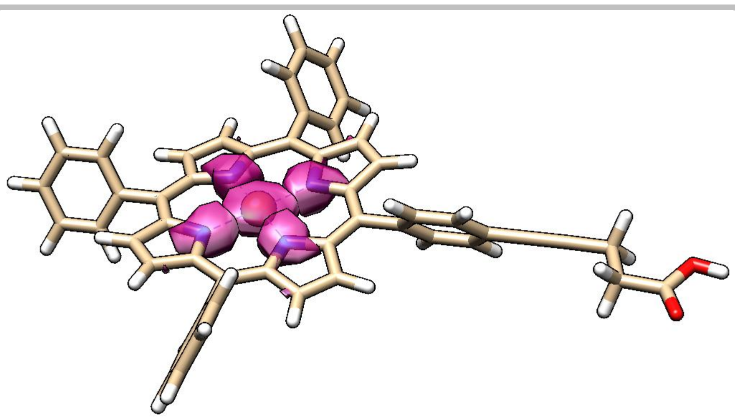

Figure S.4.3: The distribution of electronic spin density (*magenta*) in the Cu(II) porphyrin fragment of the title compound (**9**), calculated in ORCA (V.4.2.1) as described for the geometry optimised structure, and visualised using ORCA plot in UCSF Chimera with the isovalue threshold set to α = 0.001.

Orientation of the Cr_7Ni ring *g*-matrix with respect to the molecular frame

In the three spin system, the *g*-matrix of the Cr_7Ni ring is assumed to be oriented according to previously reported single-crystal studies on isolated anionic Cr_7Ni rings which have shown that the g_z component of the approximately axial *g*-matrix lies perpendicular to the plane of the ring, while the $g_x = g_y$ component lies in the plane of the ring.[14]

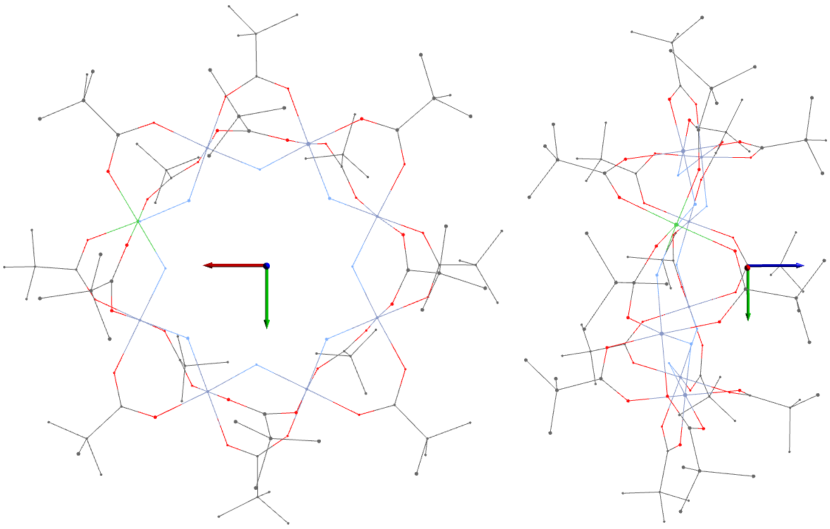

Figure S.4.4: Orientation of the *g*-matrix in the molecular frame of the Cr_7Ni ring with the xyz matrix coordinates corresponding, in order, to the RGB colour sequence. The cationic ammonium thread has been removed for clarity. In the right image, the *g*-matrix has been shifted along the z-axis away from the plane of the Cr_7Ni ring for clarity.

Orientation of the nitroxide *g*-matrix with respect to the molecular frame

The nitroxide spin has a well-documented *g*-matrix orientation and DFT results on the isolated single crystal XRD structure of the cationic ammonium thread using the BP functional in combination with the def2-SVP basis set, an RI approximation, and an empirical dispersion correction to the energies, corroborate previously reported studies.[15] The g_z component of the nitroxide *g*-matrix lies perpendicular to the nitrogen-oxygen bond and is parallel to the $2p_z$ orbital of the nitrogen atom, while the g_x component lies approximately along the nitrogen-oxygen bond. The nitroxide spin density is assumed for the simulations to be split evenly across the nitrogen-oxygen bond, however in reality the ratio of spin density across the bond is dependent on the nature of the solvent.[11]

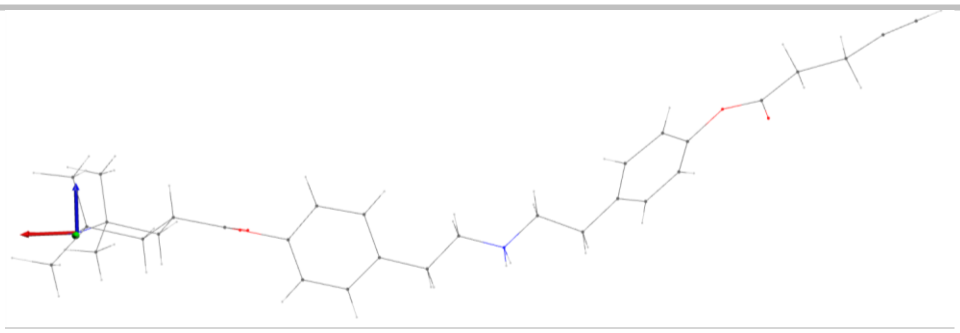

Figure S.4.5: (*Top*) Orientation of the g-matrix of the nitroxide spin in the molecular frame of the cationic ammonium thread with the xyz matrix coordinates corresponding, in order, to the RGB colour sequence. (*Bottom*) The same cationic ammonium thread viewed along the g_z axis.

Atomic coordinates of the DFT optimised single crystal XRD structure of compound (9)

ATOM	1	Cr1	UNK	1	-9.764	2.779	7.856	1.00	0.00	Cr
ATOM	2	Cr2	UNK	1	-10.036	-0.561	7.140	1.00	0.00	Cr
ATOM	3	Cr3	UNK	1	-10.155	5.782	6.279	1.00	0.00	Cr
ATOM	4	Cr4	UNK	1	-12.083	-2.039	4.881	1.00	0.00	Cr
ATOM	5	Cr5	UNK	1	-12.361	6.665	3.267	1.00	0.00	Cr
ATOM	6	Cr6	UNK	1	-13.356	5.067	0.773	1.00	0.00	Cr
ATOM	7	Cr7	UNK	1	-14.611	1.660	0.535	1.00	0.00	Cr
ATOM	8	Ni1	UNK	1	-13.797	-1.301	2.050	1.00	0.00	Ni
ATOM	9	F1	UNK	1	-9.438	1.539	6.557	1.00	0.00	F1+
ATOM	10	F2	UNK	1	-12.080	-1.010	3.382	1.00	0.00	F1+
ATOM	11	F3	UNK	1	-11.523	-0.565	5.742	1.00	0.00	F1+
ATOM	12	F4	UNK	1	-11.065	5.311	4.747	1.00	0.00	F1+
ATOM	13	F5	UNK	1	-10.645	3.817	6.684	1.00	0.00	F1+
ATOM	14	F6	UNK	1	-13.424	5.235	2.911	1.00	0.00	F1+
ATOM	15	F7	UNK	1	-13.422	3.315	1.240	1.00	0.00	F1+
ATOM	16	F8	UNK	1	-13.973	0.339	2.539	1.00	0.00	F
ATOM	17	O1	UNK	1	-8.492	-0.510	8.285	1.00	0.00	O
ATOM	18	O2	UNK	1	-13.477	4.651	-1.124	1.00	0.00	O
ATOM	19	O3	UNK	1	-11.542	4.535	0.823	1.00	0.00	O
ATOM	20	O4	UNK	1	-10.133	4.074	9.273	1.00	0.00	O
ATOM	21	O5	UNK	1	-12.838	-3.551	3.959	1.00	0.00	O
ATOM	22	O6	UNK	1	-10.013	-2.474	7.296	1.00	0.00	O

ATOM 23 O7 UNK 1 -11.314 6.836 1.617 1.00 0.00 O

ATOM 24 O8 UNK 1 -13.207 6.269 4.958 1.00 0.00 O

ATOM 25 O9 UNK 1 -15.594 2.602 1.956 1.00 0.00 O

ATOM 26 O10 UNK 1 -9.552 7.203 5.077 1.00 0.00 O

ATOM 27 O11 UNK 1 -15.215 3.018 -0.735 1.00 0.00 O

ATOM 28 O12 UNK 1 -13.856 7.541 2.374 1.00 0.00 O

ATOM 29 O13 UNK 1 -13.646 0.770 -0.828 1.00 0.00 O

ATOM 30 O14 UNK 1 -9.183 5.842 7.932 1.00 0.00 O

ATOM 31 O15 UNK 1 -12.107 -3.186 6.463 1.00 0.00 O

ATOM 32 O16 UNK 1 -13.864 -1.377 5.249 1.00 0.00 O

ATOM 33 O17 UNK 1 -15.144 -1.193 3.254 1.00 0.00 O

ATOM 34 O18 UNK 1 -11.138 8.118 3.644 1.00 0.00 O

ATOM 35 O19 UNK 1 -16.043 0.477 0.038 1.00 0.00 O

ATOM 36 O20 UNK 1 -13.592 -3.084 1.797 1.00 0.00 O

ATOM 37 O21 UNK 1 -14.848 -1.545 0.618 1.00 0.00 O

ATOM 38 O22 UNK 1 -12.615 -0.259 1.033 1.00 0.00 O

ATOM 39 O23 UNK 1 -8.107 3.741 7.470 1.00 0.00 O

ATOM 40 O24 UNK 1 -9.070 -0.772 5.429 1.00 0.00 O

ATOM 41 O25 UNK 1 -11.470 1.849 8.223 1.00 0.00 O

ATOM 42 O26 UNK 1 -11.120 -0.430 8.698 1.00 0.00 O

ATOM 43 O27 UNK 1 -11.966 6.243 6.875 1.00 0.00 O

ATOM 44 O28 UNK 1 -8.871 1.698 9.171 1.00 0.00 O

ATOM 45 O29 UNK 1 -10.296 -2.619 4.410 1.00 0.00 O

ATOM 46 O30 UNK 1 -13.214 6.866 0.222 1.00 0.00 O

ATOM 47 O31 UNK 1 -8.459 5.270 5.643 1.00 0.00 O

ATOM 48 O32 UNK 1 -15.290 4.840 1.003 1.00 0.00 O

ATOM 49 C1 UNK 1 -10.164 -4.167 9.589 1.00 0.00 C

ATOM 50 H1 UNK 1 -10.088 -5.119 10.161 1.00 0.00 H

ATOM 51 H2 UNK 1 -10.562 -3.385 10.267 1.00 0.00 H

ATOM 52 H3 UNK 1 -9.130 -3.883 9.292 1.00 0.00 H

ATOM 53 C2 UNK 1 -15.804 2.703 -3.454 1.00 0.00 C

ATOM 54 H4 UNK 1 -16.719 2.720 -2.819 1.00 0.00 H

```
ATOM    55  H5  UNK     1     -15.372   1.685  -3.424  1.00  0.00           H
ATOM    56  H6  UNK     1     -16.142   2.907  -4.498  1.00  0.00           H
ATOM    57  C3  UNK     1      -6.303   4.482   6.025  1.00  0.00           C
ATOM    58  C4  UNK     1     -14.294   9.007   0.445  1.00  0.00           C
ATOM    59  C5  UNK     1     -16.087  -0.870   0.526  1.00  0.00           C1-
ATOM    60  C6  UNK     1     -11.073  -4.348   8.350  1.00  0.00           C
ATOM    61  C7  UNK     1     -12.705  -5.313   2.358  1.00  0.00           C
ATOM    62  C8  UNK     1      -8.144  -1.910   2.602  1.00  0.00           C
ATOM    63  H7  UNK     1      -7.321  -2.211   1.917  1.00  0.00           H
ATOM    64  H8  UNK     1      -8.186  -0.810   2.620  1.00  0.00           H
ATOM    65  H9  UNK     1      -9.095  -2.278   2.155  1.00  0.00           H
ATOM    66  C9  UNK     1     -12.789   2.103  10.938  1.00  0.00           C
ATOM    67  H10 UNK     1     -13.124   2.860  10.207  1.00  0.00           H
ATOM    68  H11 UNK     1     -11.887   2.484  11.457  1.00  0.00           H
ATOM    69  H12 UNK     1     -13.597   1.977  11.696  1.00  0.00           H
ATOM    70  C10 UNK     1      -9.771   9.651   4.898  1.00  0.00           C
ATOM    71  C11 UNK     1     -14.048   7.582   6.825  1.00  0.00           C
ATOM    72  C12 UNK     1     -13.917   7.380   8.355  1.00  0.00           C
ATOM    73  H13 UNK     1     -14.781   7.832   8.891  1.00  0.00           H
ATOM    74  H14 UNK     1     -12.986   7.841   8.740  1.00  0.00           H
ATOM    75  H15 UNK     1     -13.910   6.296   8.606  1.00  0.00           H
ATOM    76  C13 UNK     1      -7.867  -4.027   3.908  1.00  0.00           C
ATOM    77  H16 UNK     1      -8.812  -4.424   3.479  1.00  0.00           H
ATOM    78  H17 UNK     1      -7.729  -4.481   4.912  1.00  0.00           H
ATOM    79  H18 UNK     1      -7.035  -4.356   3.251  1.00  0.00           H
ATOM    80  C14 UNK     1      -5.422   3.314   6.473  1.00  0.00           C
ATOM    81  H19 UNK     1      -5.679   2.402   5.926  1.00  0.00           H
ATOM    82  H20 UNK     1      -5.574   3.101   7.549  1.00  0.00           H
ATOM    83  H21 UNK     1      -4.348   3.544   6.306  1.00  0.00           H
ATOM    84  C15 UNK     1     -16.914  -1.735  -0.495  1.00  0.00           C
ATOM    85  C16 UNK     1      -9.668   6.288  10.261  1.00  0.00           C
ATOM    86  C17 UNK     1     -14.784   3.778  -3.000  1.00  0.00           C
```

```
ATOM     87  C18 UNK     1     -13.798   0.256   9.565  1.00  0.00           C
ATOM     88  H22 UNK     1     -14.614   0.136  10.312  1.00  0.00           H
ATOM     89  H23 UNK     1     -13.647  -0.732   9.081  1.00  0.00           H
ATOM     90  H24 UNK     1     -14.148   0.967   8.790  1.00  0.00           H
ATOM     91  C19 UNK     1      -5.963   4.876   4.563  1.00  0.00           C
ATOM     92  H25 UNK     1      -4.889   5.133   4.456  1.00  0.00           H
ATOM     93  H26 UNK     1      -6.537   5.774   4.241  1.00  0.00           H
ATOM     94  H27 UNK     1      -6.205   4.060   3.855  1.00  0.00           H
ATOM     95  C20 UNK     1      -5.981   5.699   6.931  1.00  0.00           C
ATOM     96  H28 UNK     1      -6.198   5.471   7.997  1.00  0.00           H
ATOM     97  H29 UNK     1      -6.576   6.593   6.645  1.00  0.00           H
ATOM     98  H30 UNK     1      -4.906   5.974   6.851  1.00  0.00           H
ATOM     99  C21 UNK     1     -11.627  -0.396  -1.357  1.00  0.00           C
ATOM    100  C22 UNK     1     -12.090  -0.262  11.330  1.00  0.00           C
ATOM    101  H31 UNK     1     -11.160   0.072  11.839  1.00  0.00           H
ATOM    102  H32 UNK     1     -11.898  -1.263  10.886  1.00  0.00           H
ATOM    103  H33 UNK     1     -12.886  -0.388  12.093  1.00  0.00           H
ATOM    104  C23 UNK     1     -16.177  -2.223   5.292  1.00  0.00           C
ATOM    105  C24 UNK     1     -16.579  -3.697   5.125  1.00  0.00           C
ATOM    106  H34 UNK     1     -17.459  -3.943   5.760  1.00  0.00           H
ATOM    107  H35 UNK     1     -15.740  -4.359   5.428  1.00  0.00           H
ATOM    108  H36 UNK     1     -16.837  -3.916   4.067  1.00  0.00           H
ATOM    109  C25 UNK     1      -9.617   6.043  -0.039  1.00  0.00           C
ATOM    110  C26 UNK     1     -15.401   6.943   6.404  1.00  0.00           C
ATOM    111  H37 UNK     1     -15.439   5.858   6.611  1.00  0.00           H
ATOM    112  H38 UNK     1     -15.602   7.112   5.328  1.00  0.00           H
ATOM    113  H39 UNK     1     -16.248   7.408   6.962  1.00  0.00           H
ATOM    114  C27 UNK     1     -14.821   8.887  -0.996  1.00  0.00           C
ATOM    115  H40 UNK     1     -15.736   8.257  -1.019  1.00  0.00           H
ATOM    116  H41 UNK     1     -14.059   8.404  -1.652  1.00  0.00           H
ATOM    117  H42 UNK     1     -15.055   9.886  -1.417  1.00  0.00           H
ATOM    118  C28 UNK     1     -17.463  -2.966   0.245  1.00  0.00           C
```

ATOM	119	H43	UNK	1	-16.636	-3.581	0.663	1.00	0.00	H
ATOM	120	H44	UNK	1	-18.055	-3.610	-0.446	1.00	0.00	H
ATOM	121	H45	UNK	1	-18.123	-2.648	1.079	1.00	0.00	H
ATOM	122	C29	UNK	1	-14.130	9.077	6.519	1.00	0.00	C
ATOM	123	H46	UNK	1	-14.990	9.545	7.048	1.00	0.00	H
ATOM	124	H47	UNK	1	-14.259	9.238	5.426	1.00	0.00	H
ATOM	125	H48	UNK	1	-13.203	9.580	6.852	1.00	0.00	H
ATOM	126	C30	UNK	1	-11.296	-5.915	2.473	1.00	0.00	C
ATOM	127	H49	UNK	1	-10.875	-5.737	3.486	1.00	0.00	H
ATOM	128	H50	UNK	1	-10.623	-5.463	1.715	1.00	0.00	H
ATOM	129	H51	UNK	1	-11.321	-7.016	2.303	1.00	0.00	H
ATOM	130	C31	UNK	1	-16.047	-2.230	-1.695	1.00	0.00	C
ATOM	131	H52	UNK	1	-15.596	-1.380	-2.250	1.00	0.00	H
ATOM	132	H53	UNK	1	-16.659	-2.824	-2.409	1.00	0.00	H
ATOM	133	H54	UNK	1	-15.222	-2.901	-1.366	1.00	0.00	H
ATOM	134	C32	UNK	1	-13.610	-6.043	3.403	1.00	0.00	C
ATOM	135	H55	UNK	1	-13.654	-7.138	3.193	1.00	0.00	H
ATOM	136	H56	UNK	1	-14.650	-5.657	3.393	1.00	0.00	H
ATOM	137	H57	UNK	1	-13.209	-5.948	4.437	1.00	0.00	H
ATOM	138	C33	UNK	1	-10.822	10.732	5.207	1.00	0.00	C
ATOM	139	H58	UNK	1	-10.370	11.749	5.181	1.00	0.00	H
ATOM	140	H59	UNK	1	-11.240	10.579	6.222	1.00	0.00	H
ATOM	141	H60	UNK	1	-11.651	10.703	4.468	1.00	0.00	H
ATOM	142	C34	UNK	1	-11.051	0.997	-1.736	1.00	0.00	C
ATOM	143	H61	UNK	1	-11.823	1.653	-2.190	1.00	0.00	H
ATOM	144	H62	UNK	1	-10.249	0.898	-2.503	1.00	0.00	H
ATOM	145	H63	UNK	1	-10.633	1.520	-0.855	1.00	0.00	H
ATOM	146	C35	UNK	1	-9.148	9.962	3.509	1.00	0.00	C
ATOM	147	H64	UNK	1	-9.921	9.981	2.710	1.00	0.00	H
ATOM	148	H65	UNK	1	-8.380	9.209	3.238	1.00	0.00	H
ATOM	149	H66	UNK	1	-8.670	10.968	3.502	1.00	0.00	H
ATOM	150	C36	UNK	1	-9.117	4.807	-0.804	1.00	0.00	C

ATOM	151	H67	UNK	1	-9.939	4.336	-1.386	1.00	0.00	H
ATOM	152	H68	UNK	1	-8.700	4.074	-0.097	1.00	0.00	H
ATOM	153	H69	UNK	1	-8.310	5.082	-1.520	1.00	0.00	H
ATOM	154	C37	UNK	1	-15.503	5.152	-3.059	1.00	0.00	C
ATOM	155	H70	UNK	1	-15.844	5.367	-4.099	1.00	0.00	H
ATOM	156	H71	UNK	1	-14.831	5.982	-2.760	1.00	0.00	H
ATOM	157	H72	UNK	1	-16.400	5.173	-2.401	1.00	0.00	H
ATOM	158	C38	UNK	1	-15.329	9.709	1.335	1.00	0.00	C
ATOM	159	H73	UNK	1	-15.559	10.724	0.952	1.00	0.00	H
ATOM	160	H74	UNK	1	-14.935	9.819	2.373	1.00	0.00	H
ATOM	161	H75	UNK	1	-16.264	9.112	1.383	1.00	0.00	H
ATOM	162	C39	UNK	1	-13.288	-5.644	0.967	1.00	0.00	C
ATOM	163	H76	UNK	1	-12.687	-5.175	0.162	1.00	0.00	H
ATOM	164	H77	UNK	1	-14.338	-5.281	0.893	1.00	0.00	H
ATOM	165	H78	UNK	1	-13.319	-6.744	0.800	1.00	0.00	H
ATOM	166	C40	UNK	1	-10.126	7.068	-1.075	1.00	0.00	C
ATOM	167	H79	UNK	1	-9.302	7.353	-1.770	1.00	0.00	H
ATOM	168	H80	UNK	1	-10.453	8.000	-0.577	1.00	0.00	H
ATOM	169	H81	UNK	1	-10.958	6.660	-1.685	1.00	0.00	H
ATOM	170	C41	UNK	1	-17.347	-1.332	4.814	1.00	0.00	C
ATOM	171	H82	UNK	1	-17.654	-1.598	3.782	1.00	0.00	H
ATOM	172	H83	UNK	1	-17.042	-0.276	4.813	1.00	0.00	H
ATOM	173	H84	UNK	1	-18.235	-1.436	5.475	1.00	0.00	H
ATOM	174	C42	UNK	1	-16.721	5.211	3.619	1.00	0.00	C
ATOM	175	H85	UNK	1	-17.540	5.741	4.157	1.00	0.00	H
ATOM	176	H86	UNK	1	-15.974	5.974	3.318	1.00	0.00	H
ATOM	177	H87	UNK	1	-16.265	4.517	4.344	1.00	0.00	H
ATOM	178	C43	UNK	1	-18.289	3.416	2.880	1.00	0.00	C
ATOM	179	H88	UNK	1	-17.800	2.701	3.573	1.00	0.00	H
ATOM	180	H89	UNK	1	-18.723	2.849	2.030	1.00	0.00	H
ATOM	181	H90	UNK	1	-19.120	3.905	3.437	1.00	0.00	H
ATOM	182	C44	UNK	1	-7.918	-2.491	4.018	1.00	0.00	C

ATOM	183	C45	UNK	1	-14.191	3.484	-1.577	1.00	0.00	C1-
ATOM	184	C46	UNK	1	-7.937	0.722	8.745	1.00	0.00	C1-
ATOM	185	C47	UNK	1	-11.317	0.859	9.226	1.00	0.00	C1-
ATOM	186	C48	UNK	1	-10.485	8.240	4.901	1.00	0.00	C1-
ATOM	187	C49	UNK	1	-10.699	5.676	1.044	1.00	0.00	C1-
ATOM	188	C50	UNK	1	-10.043	5.481	8.979	1.00	0.00	C1-
ATOM	189	C51	UNK	1	-12.501	0.759	10.245	1.00	0.00	C
ATOM	190	C52	UNK	1	-6.571	-1.992	4.563	1.00	0.00	C
ATOM	191	H91	UNK	1	-5.747	-2.266	3.869	1.00	0.00	H
ATOM	192	H92	UNK	1	-6.358	-2.430	5.559	1.00	0.00	H
ATOM	193	H93	UNK	1	-6.576	-0.891	4.664	1.00	0.00	H
ATOM	194	C53	UNK	1	-7.023	0.426	9.995	1.00	0.00	C
ATOM	195	C54	UNK	1	-12.611	-3.739	2.589	1.00	0.00	C1-
ATOM	196	C55	UNK	1	-12.848	-0.298	-0.374	1.00	0.00	C1-
ATOM	197	C56	UNK	1	-17.278	4.468	2.390	1.00	0.00	C
ATOM	198	C57	UNK	1	-6.501	-1.031	9.990	1.00	0.00	C
ATOM	199	H94	UNK	1	-7.348	-1.747	10.101	1.00	0.00	H
ATOM	200	H95	UNK	1	-5.817	-1.212	10.848	1.00	0.00	H
ATOM	201	H96	UNK	1	-5.965	-1.261	9.047	1.00	0.00	H
ATOM	202	C58	UNK	1	-7.754	0.635	11.358	1.00	0.00	C
ATOM	203	H97	UNK	1	-8.110	1.682	11.483	1.00	0.00	H
ATOM	204	H98	UNK	1	-7.062	0.441	12.208	1.00	0.00	H
ATOM	205	H99	UNK	1	-8.624	-0.046	11.464	1.00	0.00	H
ATOM	206	C59	UNK	1	-18.088	-0.927	-1.084	1.00	0.00	C
ATOM	207	H100	UNK	1	-18.732	-0.522	-0.278	1.00	0.00	H
ATOM	208	H101	UNK	1	-18.702	-1.562	-1.759	1.00	0.00	H
ATOM	209	H102	UNK	1	-17.705	-0.081	-1.699	1.00	0.00	H
ATOM	210	C60	UNK	1	-5.834	1.388	9.928	1.00	0.00	C
ATOM	211	H103	UNK	1	-5.273	1.221	8.985	1.00	0.00	H
ATOM	212	H104	UNK	1	-5.142	1.221	10.781	1.00	0.00	H
ATOM	213	H105	UNK	1	-6.185	2.442	9.960	1.00	0.00	H
ATOM	214	C61	UNK	1	-12.193	-1.034	-2.645	1.00	0.00	C

ATOM	215	H106	UNK	1	-13.002	-0.402	-3.075	1.00	0.00	H
ATOM	216	H107	UNK	1	-12.628	-2.030	-2.416	1.00	0.00	H
ATOM	217	H108	UNK	1	-11.410	-1.162	-3.421	1.00	0.00	H
ATOM	218	C62	UNK	1	-10.466	-1.266	-0.839	1.00	0.00	C
ATOM	219	H109	UNK	1	-9.659	-1.307	-1.611	1.00	0.00	H
ATOM	220	H110	UNK	1	-10.790	-2.306	-0.637	1.00	0.00	H
ATOM	221	H111	UNK	1	-10.019	-0.825	0.075	1.00	0.00	H
ATOM	222	C63	UNK	1	-18.006	5.508	1.512	1.00	0.00	C
ATOM	223	H112	UNK	1	-18.840	5.993	2.065	1.00	0.00	H
ATOM	224	H113	UNK	1	-18.416	5.031	0.598	1.00	0.00	H
ATOM	225	H114	UNK	1	-17.307	6.317	1.199	1.00	0.00	H
ATOM	226	C64	UNK	1	-10.425	-5.431	7.448	1.00	0.00	C
ATOM	227	H115	UNK	1	-9.420	-5.118	7.089	1.00	0.00	H
ATOM	228	H116	UNK	1	-11.056	-5.662	6.565	1.00	0.00	H
ATOM	229	H117	UNK	1	-10.289	-6.381	8.017	1.00	0.00	H
ATOM	230	C65	UNK	1	-12.982	9.826	0.444	1.00	0.00	C
ATOM	231	H118	UNK	1	-12.222	9.370	-0.221	1.00	0.00	H
ATOM	232	H119	UNK	1	-12.552	9.913	1.463	1.00	0.00	H
ATOM	233	H120	UNK	1	-13.176	10.853	0.062	1.00	0.00	H
ATOM	234	C66	UNK	1	-8.422	6.667	0.713	1.00	0.00	C
ATOM	235	H121	UNK	1	-8.000	5.938	1.439	1.00	0.00	H
ATOM	236	H122	UNK	1	-8.735	7.572	1.273	1.00	0.00	H
ATOM	237	H123	UNK	1	-7.610	6.960	0.012	1.00	0.00	H
ATOM	238	C67	UNK	1	-9.084	-2.127	4.983	1.00	0.00	C1-
ATOM	239	C68	UNK	1	-7.814	4.100	6.119	1.00	0.00	C1-
ATOM	240	C69	UNK	1	-16.165	3.820	1.498	1.00	0.00	C1-
ATOM	241	C70	UNK	1	-12.787	7.027	6.042	1.00	0.00	C1-
ATOM	242	C71	UNK	1	-11.272	-2.983	7.600	1.00	0.00	C1-
ATOM	243	C72	UNK	1	-14.870	-1.956	4.439	1.00	0.00	C1-
ATOM	244	C73	UNK	1	-14.108	7.584	0.981	1.00	0.00	C1-
ATOM	245	C74	UNK	1	-10.925	6.329	11.134	1.00	0.00	C
ATOM	246	H124	UNK	1	-11.757	6.799	10.565	1.00	0.00	H

ATOM	247	H125	UNK	1	-10.752	6.920	12.057	1.00	0.00	H
ATOM	248	H126	UNK	1	-11.234	5.300	11.419	1.00	0.00	H
ATOM	249	C75	UNK	1	-9.241	7.736	9.913	1.00	0.00	C
ATOM	250	H127	UNK	1	-8.294	7.742	9.326	1.00	0.00	H
ATOM	251	H128	UNK	1	-9.045	8.323	10.838	1.00	0.00	H
ATOM	252	H129	UNK	1	-10.020	8.260	9.321	1.00	0.00	H
ATOM	253	C76	UNK	1	-8.505	5.637	11.053	1.00	0.00	C
ATOM	254	H130	UNK	1	-8.749	4.598	11.358	1.00	0.00	H
ATOM	255	H131	UNK	1	-8.300	6.215	11.983	1.00	0.00	H
ATOM	256	H132	UNK	1	-7.567	5.617	10.456	1.00	0.00	H
ATOM	257	C77	UNK	1	-15.970	-1.959	6.811	1.00	0.00	C
ATOM	258	H133	UNK	1	-16.926	-2.090	7.369	1.00	0.00	H
ATOM	259	H134	UNK	1	-15.629	-0.918	7.005	1.00	0.00	H
ATOM	260	H135	UNK	1	-15.235	-2.666	7.253	1.00	0.00	H
ATOM	261	C78	UNK	1	-12.449	-4.848	8.811	1.00	0.00	C
ATOM	262	H136	UNK	1	-12.358	-5.803	9.372	1.00	0.00	H
ATOM	263	H137	UNK	1	-13.109	-5.030	7.936	1.00	0.00	H
ATOM	264	H138	UNK	1	-12.930	-4.092	9.467	1.00	0.00	H
ATOM	265	C79	UNK	1	-13.602	3.780	-3.974	1.00	0.00	C
ATOM	266	H139	UNK	1	-13.102	2.787	-3.952	1.00	0.00	H
ATOM	267	H140	UNK	1	-12.864	4.558	-3.688	1.00	0.00	H
ATOM	268	H141	UNK	1	-13.944	3.976	-5.014	1.00	0.00	H
ATOM	269	C80	UNK	1	-8.637	9.760	5.945	1.00	0.00	C
ATOM	270	H142	UNK	1	-8.176	10.775	5.908	1.00	0.00	H
ATOM	271	H143	UNK	1	-7.824	9.034	5.729	1.00	0.00	H
ATOM	272	H144	UNK	1	-9.010	9.593	6.976	1.00	0.00	H
ATOM	273	O33	UNK	1	-4.777	-0.822	0.887	1.00	0.00	O
ATOM	274	O34	UNK	1	1.356	-0.085	-0.026	1.00	0.00	O1-
ATOM	275	O35	UNK	1	-17.304	2.891	9.661	1.00	0.00	O
ATOM	276	O36	UNK	1	-17.143	5.079	10.124	1.00	0.00	O
ATOM	277	O37	UNK	1	-4.587	1.039	2.108	1.00	0.00	O
ATOM	278	N1	UNK	1	-12.134	2.437	3.736	1.00	0.00	N1+

ATOM 279 H145 UNK 1 -12.612 1.966 2.953 1.00 0.00 H

ATOM 280 H146 UNK 1 -12.115 3.458 3.539 1.00 0.00 H

ATOM 281 C81 UNK 1 -14.226 2.867 4.937 1.00 0.00 C

ATOM 282 H147 UNK 1 -14.811 2.377 4.143 1.00 0.00 H

ATOM 283 H148 UNK 1 -14.101 3.930 4.685 1.00 0.00 H

ATOM 284 N2 UNK 1 0.000 0.000 0.000 1.00 0.00 N

ATOM 285 C82 UNK 1 -8.628 1.858 2.325 1.00 0.00 C

ATOM 286 C83 UNK 1 -7.840 1.686 3.473 1.00 0.00 C

ATOM 287 H149 UNK 1 -8.237 1.743 4.466 1.00 0.00 H

ATOM 288 C84 UNK 1 -8.021 1.763 1.059 1.00 0.00 C

ATOM 289 H150 UNK 1 -8.605 1.883 0.157 1.00 0.00 H

ATOM 290 C85 UNK 1 -5.896 1.328 2.117 1.00 0.00 C

ATOM 291 C86 UNK 1 -4.054 0.011 1.423 1.00 0.00 C

ATOM 292 C87 UNK 1 -0.440 1.191 0.776 1.00 0.00 C

ATOM 293 C88 UNK 1 -2.559 -0.104 1.378 1.00 0.00 C

ATOM 294 H151 UNK 1 -2.200 -0.294 2.412 1.00 0.00 H

ATOM 295 C89 UNK 1 -17.560 3.969 10.434 1.00 0.00 C

ATOM 296 C90 UNK 1 -16.508 2.879 8.574 1.00 0.00 C

ATOM 297 C91 UNK 1 -16.970 2.197 7.452 1.00 0.00 C

ATOM 298 H152 UNK 1 -17.932 1.701 7.481 1.00 0.00 H

ATOM 299 C92 UNK 1 -15.268 3.540 8.531 1.00 0.00 C

ATOM 300 H153 UNK 1 -14.896 4.079 9.393 1.00 0.00 H

ATOM 301 C93 UNK 1 -1.981 1.222 0.865 1.00 0.00 C

ATOM 302 H154 UNK 1 -2.299 2.073 1.510 1.00 0.00 H

ATOM 303 H155 UNK 1 -2.405 1.399 -0.149 1.00 0.00 H

ATOM 304 C94 UNK 1 -18.507 3.786 11.581 1.00 0.00 C

ATOM 305 H156 UNK 1 -19.433 3.328 11.168 1.00 0.00 H

ATOM 306 H157 UNK 1 -18.064 3.091 12.326 1.00 0.00 H

ATOM 307 C95 UNK 1 -2.137 -1.219 0.411 1.00 0.00 C

ATOM 308 H158 UNK 1 -2.573 -0.955 -0.581 1.00 0.00 H

ATOM 309 H159 UNK 1 -2.574 -2.198 0.715 1.00 0.00 H

ATOM 310 C96 UNK 1 -0.336 -2.225 -0.960 1.00 0.00 C

ATOM 311 H160 UNK 1 -0.762 -1.750 -1.872 1.00 0.00 H

ATOM 312 H161 UNK 1 -0.807 -3.226 -0.845 1.00 0.00 H

ATOM 313 H162 UNK 1 0.751 -2.362 -1.127 1.00 0.00 H

ATOM 314 C97 UNK 1 -0.009 -2.046 1.514 1.00 0.00 C

ATOM 315 H163 UNK 1 1.096 -2.111 1.447 1.00 0.00 H

ATOM 316 H164 UNK 1 -0.403 -3.082 1.581 1.00 0.00 H

ATOM 317 H165 UNK 1 -0.282 -1.573 2.467 1.00 0.00 H

ATOM 318 C98 UNK 1 -10.744 1.992 3.781 1.00 0.00 C

ATOM 319 H166 UNK 1 -10.214 2.615 4.524 1.00 0.00 H

ATOM 320 H167 UNK 1 -10.681 0.922 4.060 1.00 0.00 H

ATOM 321 C99 UNK 1 -14.984 2.839 6.228 1.00 0.00 C

ATOM 322 C100 UNK 1 -12.866 2.192 4.991 1.00 0.00 C

ATOM 323 H168 UNK 1 -12.306 2.601 5.838 1.00 0.00 H

ATOM 324 H169 UNK 1 -13.021 1.111 5.154 1.00 0.00 H

ATOM 325 C101 UNK 1 -16.224 2.194 6.284 1.00 0.00 C

ATOM 326 H170 UNK 1 -16.622 1.707 5.414 1.00 0.00 H

ATOM 327 C102 UNK 1 -14.500 3.505 7.364 1.00 0.00 C

ATOM 328 H171 UNK 1 -13.550 4.024 7.334 1.00 0.00 H

ATOM 329 C103 UNK 1 -6.654 1.497 0.949 1.00 0.00 C

ATOM 330 H172 UNK 1 -6.201 1.420 -0.032 1.00 0.00 H

ATOM 331 C104 UNK 1 -6.489 1.431 3.352 1.00 0.00 C

ATOM 332 H173 UNK 1 -5.880 1.277 4.217 1.00 0.00 H

ATOM 333 C105 UNK 1 -0.606 -1.336 0.271 1.00 0.00 C

ATOM 334 C106 UNK 1 0.000 2.451 -0.000 1.00 0.00 C

ATOM 335 H174 UNK 1 1.106 2.500 -0.089 1.00 0.00 H

ATOM 336 H175 UNK 1 -0.350 3.377 0.506 1.00 0.00 H

ATOM 337 H176 UNK 1 -0.424 2.434 -1.028 1.00 0.00 H

ATOM 338 C107 UNK 1 0.203 1.277 2.188 1.00 0.00 C

ATOM 339 H177 UNK 1 -0.071 0.452 2.859 1.00 0.00 H

ATOM 340 H178 UNK 1 -0.132 2.203 2.703 1.00 0.00 H

ATOM 341 H179 UNK 1 1.309 1.315 2.121 1.00 0.00 H

ATOM 342 C108 UNK 1 -10.095 2.158 2.400 1.00 0.00 C

```
ATOM    343 H180 UNK     1     -10.186   3.204   2.089  1.00  0.00           H
ATOM    344 H181 UNK     1     -10.640   1.504   1.685  1.00  0.00           H
ATOM    345 C109 UNK     1     -18.604   4.924  14.944  1.00  0.00           C
ATOM    346 C110 UNK     1     -18.860   5.113  12.233  1.00  0.00           C
ATOM    347 H182 UNK     1     -18.278   5.950  11.796  1.00  0.00           H
ATOM    348 H183 UNK     1     -19.944   5.310  12.075  1.00  0.00           H
ATOM    349 C111 UNK     1     -18.691   5.054  13.678  1.00  0.00           C
ATOM    350 C112 UNK     1     -18.260   4.332  20.754  1.00  0.00           C
ATOM    351 C113 UNK     1     -21.388   4.002  24.331  1.00  0.00           C
ATOM    352 C114 UNK     1     -17.792   3.622  27.584  1.00  0.00           C
ATOM    353 C115 UNK     1     -14.528   3.967  23.857  1.00  0.00           C
ATOM    354  N3  UNK     1     -19.255   3.792  25.672  1.00  0.00           N
ATOM    355  N4  UNK     1     -19.485   4.166  22.846  1.00  0.00           N1+
ATOM    356  N5  UNK     1     -16.618   4.072  22.659  1.00  0.00           N
ATOM    357  N6  UNK     1     -16.462   3.880  25.472  1.00  0.00           N1+
ATOM    358 C116 UNK     1     -15.262   4.032  22.729  1.00  0.00           C
ATOM    359 C117 UNK     1     -16.911   4.189  21.344  1.00  0.00           C
ATOM    360 C118 UNK     1     -19.397   4.319  21.493  1.00  0.00           C
ATOM    361 C119 UNK     1     -20.819   4.168  23.120  1.00  0.00           C
ATOM    362 C120 UNK     1     -15.137   3.913  25.200  1.00  0.00           C
ATOM    363 C121 UNK     1     -16.548   3.762  26.813  1.00  0.00           C
ATOM    364 C122 UNK     1     -19.005   3.636  27.003  1.00  0.00           C
ATOM    365 C123 UNK     1     -20.606   3.798  25.566  1.00  0.00           C
ATOM    366 C124 UNK     1     -14.714   4.046  21.456  1.00  0.00           C
ATOM    367 C125 UNK     1     -15.752   4.139  20.587  1.00  0.00           C
ATOM    368 C126 UNK     1     -20.662   4.486  20.948  1.00  0.00           C
ATOM    369 C127 UNK     1     -21.550   4.390  21.963  1.00  0.00           C
ATOM    370 C128 UNK     1     -21.195   3.570  26.797  1.00  0.00           C
ATOM    371 C129 UNK     1     -20.189   3.471  27.701  1.00  0.00           C
ATOM    372 C130 UNK     1     -14.399   3.899  26.370  1.00  0.00           C
ATOM    373 C131 UNK     1     -15.292   3.805  27.390  1.00  0.00           C
ATOM    374 H184 UNK     1     -13.672   3.990  21.170  1.00  0.00           H
```

ATOM	375	H185	UNK	1	-15.651	4.169	19.511	1.00	0.00	H
ATOM	376	H186	UNK	1	-20.936	4.668	19.917	1.00	0.00	H
ATOM	377	H187	UNK	1	-22.622	4.481	21.849	1.00	0.00	H
ATOM	378	H188	UNK	1	-22.247	3.484	27.030	1.00	0.00	H
ATOM	379	H189	UNK	1	-20.326	3.292	28.760	1.00	0.00	H
ATOM	380	H190	UNK	1	-13.326	3.949	26.483	1.00	0.00	H
ATOM	381	H191	UNK	1	-15.036	3.769	28.441	1.00	0.00	H
ATOM	382	C132	UNK	1	-18.343	4.485	19.265	1.00	0.00	C
ATOM	383	C133	UNK	1	-17.670	5.536	18.613	1.00	0.00	C
ATOM	384	C134	UNK	1	-19.089	3.577	18.495	1.00	0.00	C
ATOM	385	H192	UNK	1	-17.107	6.263	19.187	1.00	0.00	H
ATOM	386	H193	UNK	1	-19.593	2.742	18.969	1.00	0.00	H
ATOM	387	C135	UNK	1	-17.733	5.664	17.223	1.00	0.00	C
ATOM	388	C136	UNK	1	-19.183	3.728	17.109	1.00	0.00	C
ATOM	389	H194	UNK	1	-17.224	6.489	16.738	1.00	0.00	H
ATOM	390	H195	UNK	1	-19.754	3.013	16.531	1.00	0.00	H
ATOM	391	C137	UNK	1	-18.499	4.766	16.472	1.00	0.00	C
ATOM	392	C138	UNK	1	-22.881	4.007	24.456	1.00	0.00	C
ATOM	393	C139	UNK	1	-23.648	3.023	23.811	1.00	0.00	C
ATOM	394	C140	UNK	1	-23.527	4.990	25.225	1.00	0.00	C
ATOM	395	H196	UNK	1	-23.167	2.249	23.228	1.00	0.00	H
ATOM	396	H197	UNK	1	-22.955	5.762	25.724	1.00	0.00	H
ATOM	397	C141	UNK	1	-25.042	3.021	23.936	1.00	0.00	C
ATOM	398	C142	UNK	1	-24.923	4.994	25.333	1.00	0.00	C
ATOM	399	H198	UNK	1	-25.625	2.253	23.445	1.00	0.00	H
ATOM	400	H199	UNK	1	-25.418	5.761	25.915	1.00	0.00	H
ATOM	401	C143	UNK	1	-25.678	4.008	24.693	1.00	0.00	C
ATOM	402	H200	UNK	1	-26.756	4.008	24.783	1.00	0.00	H
ATOM	403	C144	UNK	1	-17.673	3.464	29.063	1.00	0.00	C
ATOM	404	C145	UNK	1	-16.960	2.429	29.614	1.00	0.00	C
ATOM	405	C146	UNK	1	-18.206	4.439	29.924	1.00	0.00	C
ATOM	406	H201	UNK	1	-16.608	1.581	28.963	1.00	0.00	H

ATOM	407	H202	UNK	1	-18.690	5.313	29.519	1.00	0.00	H
ATOM	408	C147	UNK	1	-16.910	2.205	30.996	1.00	0.00	C
ATOM	409	C148	UNK	1	-18.080	4.303	31.308	1.00	0.00	C
ATOM	410	H203	UNK	1	-16.417	1.337	31.414	1.00	0.00	H
ATOM	411	H204	UNK	1	-18.479	5.064	31.964	1.00	0.00	H
ATOM	412	C149	UNK	1	-17.436	3.183	31.845	1.00	0.00	C
ATOM	413	H205	UNK	1	-17.346	3.076	32.918	1.00	0.00	H
ATOM	414	C150	UNK	1	-13.039	3.953	23.775	1.00	0.00	C
ATOM	415	C151	UNK	1	-12.352	5.058	23.241	1.00	0.00	C
ATOM	416	C152	UNK	1	-12.307	2.844	24.234	1.00	0.00	C
ATOM	417	H206	UNK	1	-12.898	5.925	22.897	1.00	0.00	H
ATOM	418	H207	UNK	1	-12.820	1.981	24.640	1.00	0.00	H
ATOM	419	C153	UNK	1	-10.957	5.053	23.176	1.00	0.00	C
ATOM	420	C154	UNK	1	-10.913	2.838	24.151	1.00	0.00	C
ATOM	421	H208	UNK	1	-10.434	5.913	22.776	1.00	0.00	H
ATOM	422	H209	UNK	1	-10.354	1.976	24.491	1.00	0.00	H
ATOM	423	C155	UNK	1	-10.238	3.943	23.624	1.00	0.00	C
ATOM	424	H210	UNK	1	-9.158	3.939	23.565	1.00	0.00	H
ATOM	425	Cu1	UNK	1	-17.944	3.989	24.144	1.00	0.00	Cu

S.5 - Orientation dependent dipolar simulations

The initial input for orientation dependent simulations was derived from the single crystal XRD structure of [2]-rotaxane-TEMPO **(8)**. The spin density of the Cr_7Ni ring was arranged equally at eight points around a ring of radius 0.45 nm (the average radius from single crystal XRD data), while the spin is localised within each molecule to the site of the nickle ion in the Cr_7Ni ring, the ensemble nature of the EPR measurement means that within the frozen solution sample statistically there will be multiple molecules with near identical global conformational geometry in which the nickle ion is located at a different site in each molecule, averaging these molecular conformations results in a representation where the spin density is delocalised over the eight metal ions in the Cr_7Ni ring. Performing a single calculation in which the spin density is equally distributed over all metal sites in the ring is computationally more efficient than performing eight calculations localising the spin density to a single site each time and averaging the results, however in each case the results are mathematically identical. A similar approach has previously been adopted in simulating Cr_7Ni ring systems.[8] For the Cu(II) porphyrin, the delocalisation of the spin density, and lowest energy conformation of the CH_2-CH_2 linker (dihedral A) was taken from the DFT calculations as described in **S.4**. The spin density was assigned as $\sigma = 0.6$ on Cu and $\sigma = 0.1$ on each nitrogen atom. Initially, a distribution of spin centres in a three spin geometric model in the *g*-frame of the Cr_7Ni ring, g_z = [0 0 1], was constructed using the '*sphrand*' function implemented in *Easyspin*[6] to randomly pick points on the unit sphere within a pre-defined cone angle centred around an inter-spin vector beginning at the origin i.e. the centre point of the Cr_7Ni ring, chosen to represent the centre of the delocalised spin density of the ring (Fig. S.5.1 and S.5.2). The length of these inter-spin vectors is varied within a given range and the angle that the g_z component of the dipolar coupled spin makes with this vector is rotated away from the inter-spin vector within the cone. This angle is also allowed to vary within a given range. This gives a restricted distribution of oriented spin centres relative to the Cr_7Ni *g*-frame (Fig. S.5.3). In order to run the orientation dependent simulation algorithm described previously, the input parameters need to be in the *g*-frame of the detection spin – in this case either the Cu(II) or nitroxide *g*-frame. For example, taking Cu(II) as the detection spin, the molecular coordinates in the *g*-frame of the Cr_7Ni are translated so that the Cu(II) spin is located at the origin, and then the entire spin system (coordinates and *g*-matrices) is rotated (using an active rotation) into the Cu(II) *g*-frame (Fig. S.5.4).

GitHub Repository:

https://github.com/ciaranrogers16/MATLAB-scripts-for-EPR/tree/main/Modelling-conformational-flexibility-in-a-spectrally-addressable-multi-spin-molecular-qubit

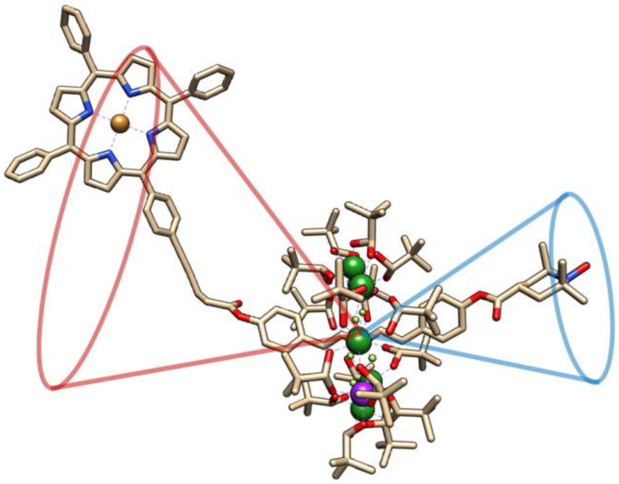

Figure S.5.1: Geometric conical model used to sample the conformational space of the most probable orientations of the respective spin centres relative to the centre of the Cr_7Ni ring used in the orientation dependent algorithm simulation input. The cone angle used was varied, and initially set to 90 degrees to capture any potential secondary confirmation before restriction to a smaller angle based on the expected confirmations from the DFT model.

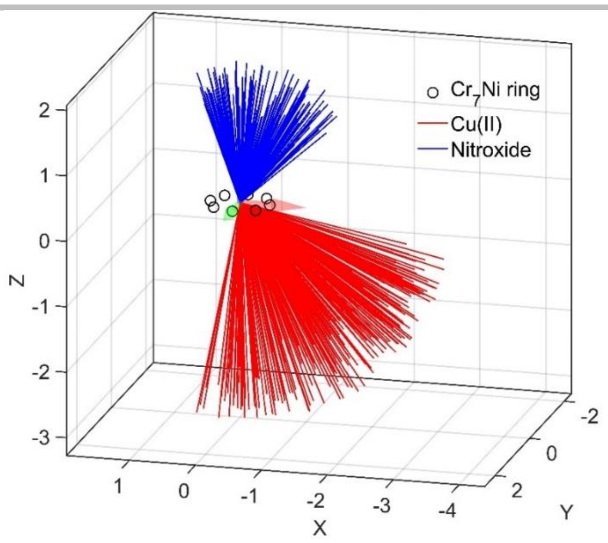

Figure S.5.2: Plot of a random sample set of conically distributed Cu(II) (red lines) and nitroxide (blue lines) inter-spin vectors relative to the centre of spin density of the Cr_7Ni ring (*g*-matrix for ring shown, following an RGB=xyz colour sequence).

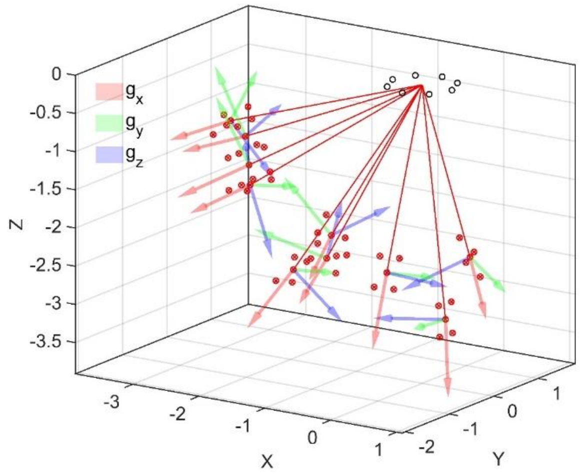

Figure S.5.3: Plot of some randomly distributed Cu(II) spin centres (red circles) and interspin vectors (red lines) in the *g*-frame of the Cr_7Ni ring (black) (Cu(II) *g*-matrices shown following an RGB=xyz colour sequence).

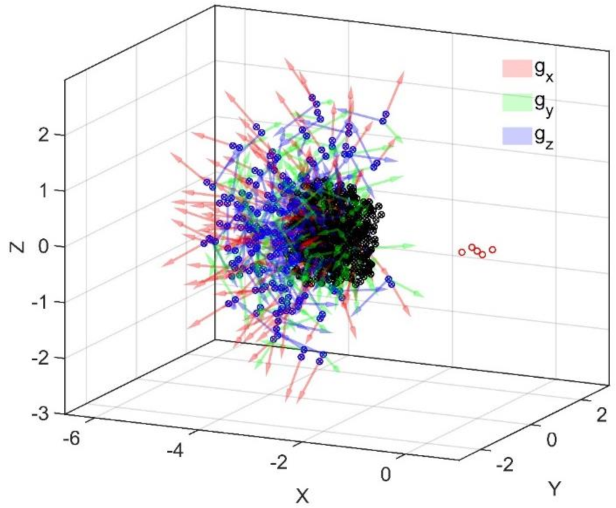

Figure S.5.4: Plot of some randomly distrubted translated and rotated Cr_7Ni (black circles) and nitroxide (blue circles) spin centres, with their *g*-matrices shown (RGB=xyz colour sequence), relative to the the *g*-frame of the Cu(II) spin centre (red circles).

Dipolar distance distribution data

Table S.5.1: Most probable distances between dipolar coupled spins obtained – **a)** DEER Analysis: orientation independent Tikhonov Regularisation. **b)** Scaling factor to take into account deviation of g-values from that of the free electron *g*-value, g_e. **c)** *g*-value corrected DEERAnalysis distances. **d)** Distances corresponding to best fititng traces from orientation dependent iterative fitting algorithm. **e)** DFT derived distances from optimised single crystal XRD structure.

Dipolar coupled spin pair	DEER Analysis distance ($r_{2.0023}$) / nm	Scaling factor (g_{eff} = $g_{pump/relax}$)	g-value corrected distance (r_{gcorr}) / nm	Average distances obtained from best fitting simulated traces / nm	DFT optimised XRD structure distance / nm
Cu(II)-Cr_7Ni	2.34	0.98	2.29	2.16-2.18	2.07
NO-Cr_7Ni	1.54	0.96	1.48	1.28-1.38	1.35
Cu(II)-NO	3.05	0.99	3.08	ca. 2.7	3.01

os-RIDME Cu(II)-Cr_7Ni ring at 3 K (34 GHz) – orientation dependent simulations

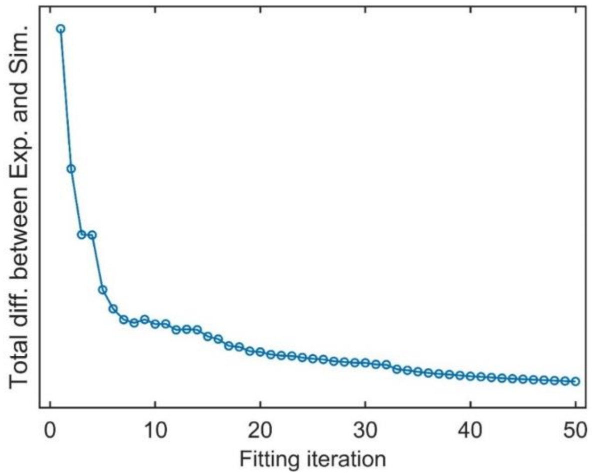

Figure S.5.5: Total difference between experimental and simulated traces vs. fitting iteration, leading to the least-square fits shown in the main text for the Cu(II)-Cr_7Ni os-RIDME experiments (Fig. 3, b).

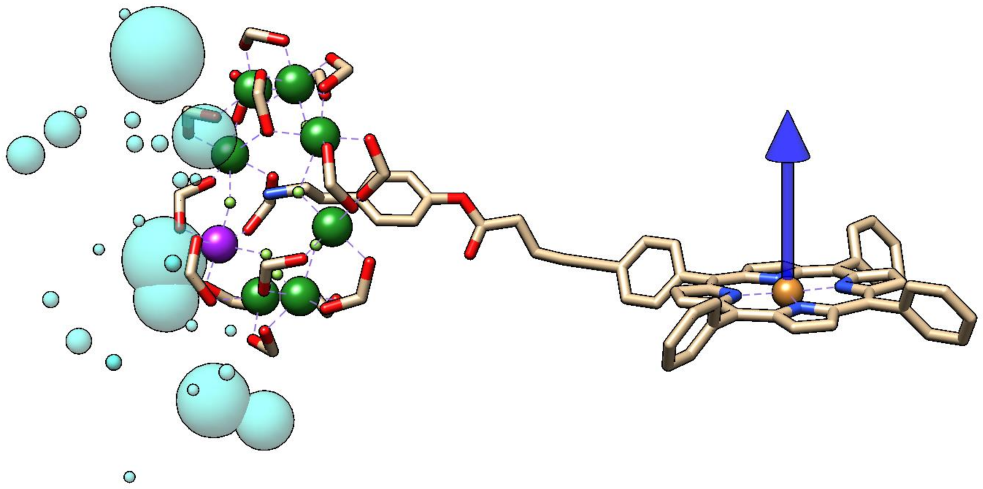

Figure S.5.6: Distribution of the centre of Cr_7Ni ring spin density relative to the centre of spin density, in the *g*-frame, of the Cu(II) spin. The larger the sphere, the larger the contribution to the final time domain fits, shown in the main text for the Cu(II)-Cr_7Ni os-RIDME experiments (Fig. 3, b). The nitroxide fragment, H atoms, and *tert*-butyl groups have been removed.

os-RIDME NO-Cr7Ni ring at 3 K (34 GHz) – orientation dependent simulations

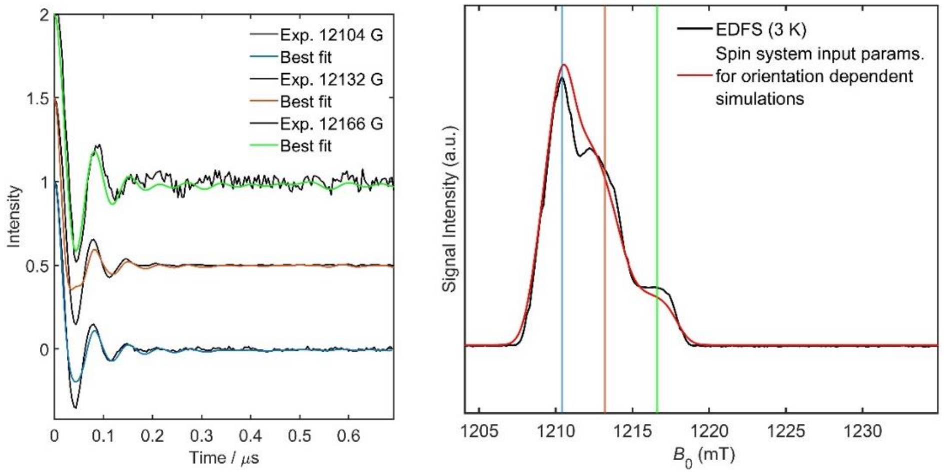

Figure S.5.7: (*Left*) Best fitting simulations (coloured) to experimental RIDME data (black) at three representive field positions across the nitroxide spectrum. At a field position of 12132 G (orange), the algorithm attempts to fit simulated traces with a higher frequency component that is not present in the experimental trace. (*Right*) Comparing the EDFS spectrum at 3 K (black) and the CW derived simulation input parameters for the orientation dependent algorithm (red), we reason that the excited sub-populations are most probably not accurately represented at this field position due to slight deviations between the experimental field swept spectrum and the simulation spin system input.

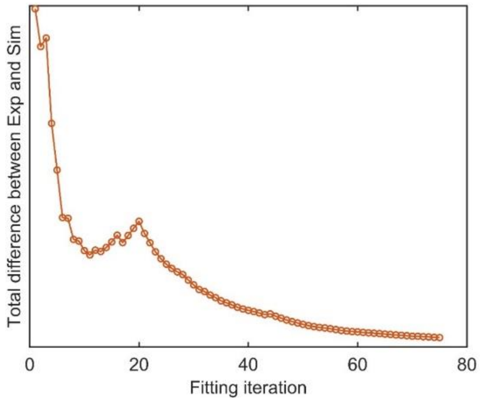

Figure S.5.8: Total difference between experimental and simulated traces vs. fitting iteration, leading to the least-square fits shown in the main text for the NO-Cr_7Ni os-RIDME experiments (Fig. 5, a).

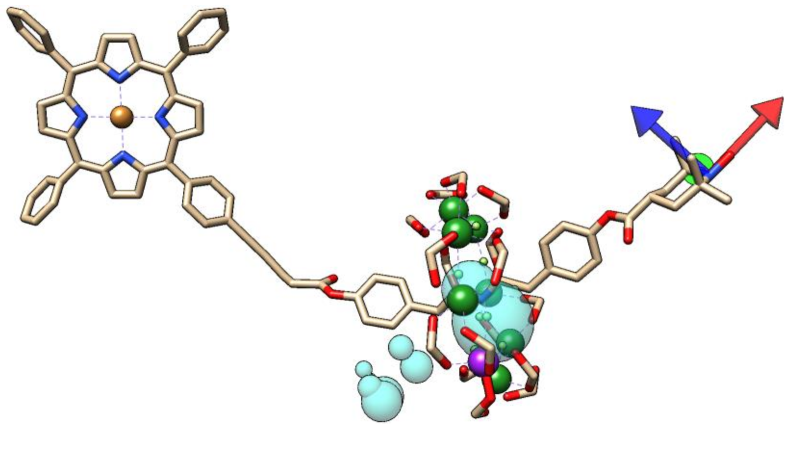

Figure S.5.9: Distribution of the centre of Cr_7Ni ring spin density relative to the nitroxide *g*-matrix. The larger the sphere, the larger the contribution to the final time domain fits for the NO-Cr_7Ni RIDME experiments H atoms, and *tert*-butyl groups have been removed.

os-DEER Cu(II)-NO at 50 K and 15 K (X-band) orientation dependent simulations

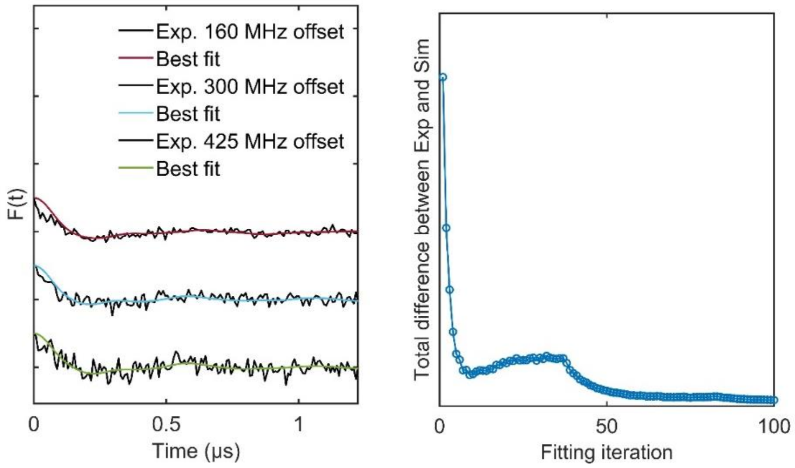

Figure S.5.10: (*Left*) Best fitting simulations (coloured) to experimental os-DEER (50 K) data (black) at three representive offsets of the pump pulse from the detection pulse – comparable to those used at 15 K – centred at the maximum of the nitroxide spectrum at X-band (322 mT). (*Right*) Total difference between experimental and simulated traces vs. fitting iteration, leading to the least-square fits shown for Cu(II)-nitroxide os-DEER at 50 K.

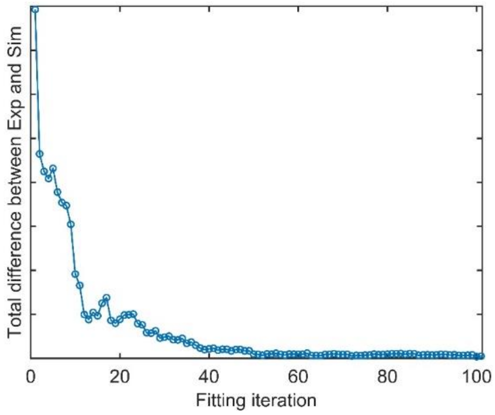

Figure S.5.11: Total difference between experimental and simulated traces vs. fitting iteration, leading to the least-square fits shown for Cu(II)-nitroxide os-DEER at 15 K as presented in the main text (Fig. 4, a).

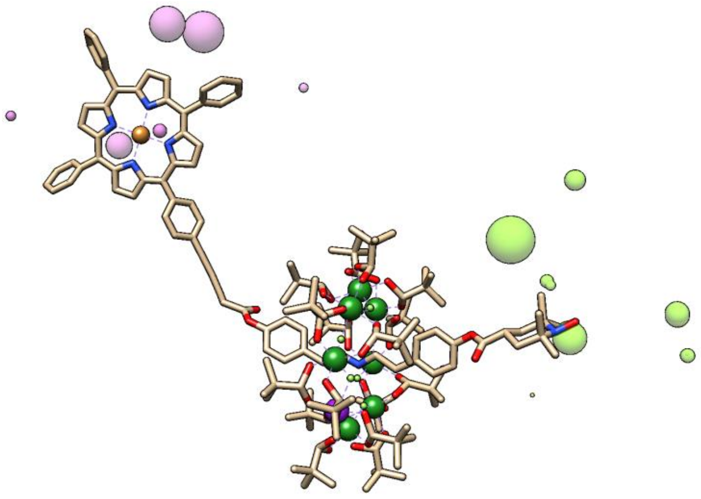

Figure S.5.12: Self consitent model of best fitting traces from orientation dependent algorithm represented as most probable centres of spin densities of Cu(II) and nitroxide in the detection frame of the Cr_7Ni ring for Cu(II)-Cr_7Ni ring RIDME at 3 K (purple spheres) and NO-Cr_7Ni ring RIDME at 3 K.

RIDME Cu(II)-NO at 6 K (34 GHz)

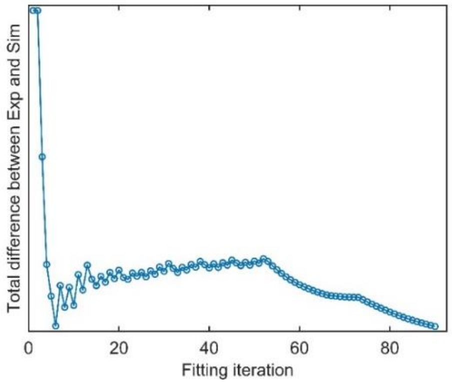

Figure S.5.13: Total difference between experimental and simulated traces vs. fitting iteration, leading to the least-square fits shown for Cu(II)-nitroxide RIDME at 6 K as presented in the main text (Fig. 5, a).

Temperature dependent os-RIDME (34 GHz)

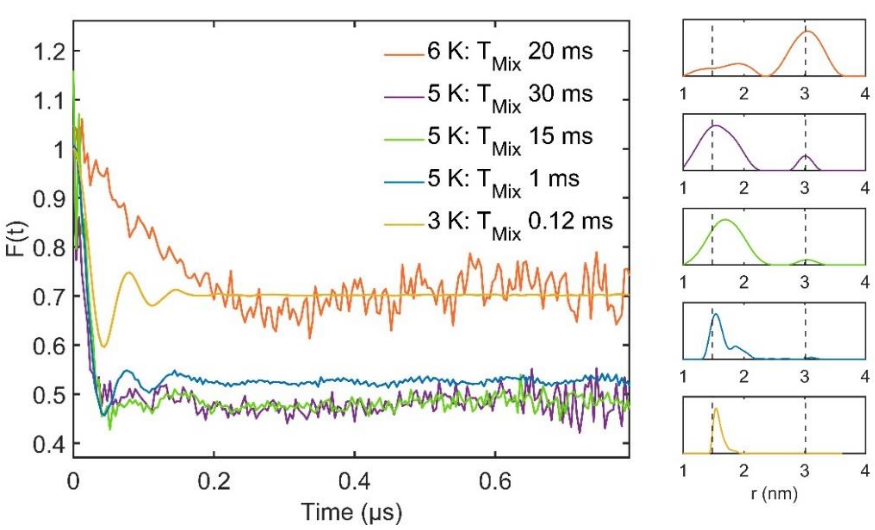

Figure S.5.14: (*Left*) Normalised, phased and background corrected RIDME Form factors detecting on the nitroxide maximum (1210.4 mT) at 34 GHz with varying T_{mix} and temperature. (*Right*) Orientation independent DEERAnalysis derived distance distributions obtained via orientation independent Tikhonov regularisation – purely to visualise distance domain representation of experimental traces (coloured) with the expected NO-Cr_7Ni and NO-Cu(II) distances from the DFT optimised XRD structure shown (black dashed lines).

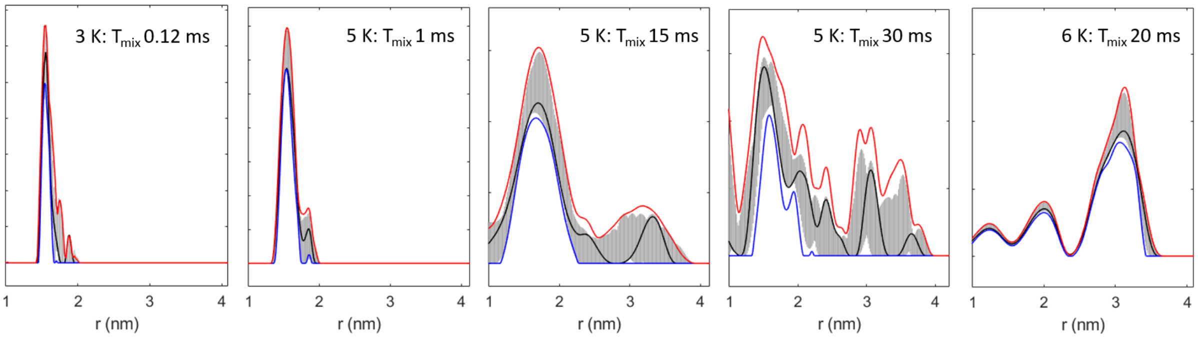

Figure S.5.15: Validation of DEERAnalysis orientation independent distance distributions after Tikhonov Regularisation for background corrected and phased temperature dependent os-RIDME traces (Fig S.5.14). Black = best fits; Grey = error bars; Blue = lower error estimate corresponding to the mean value of the probability minus two times its standard deviation; Red = upper error estimate corresponding to the mean value plus two times the standard deviation.

Modulation depth and Form factor analysis in temperature dependent/multi-spin RIDME

Increased dipolar modulation depths are often associated with the presence of multi-spin effects (MSE) which result in sum and difference combinations to the pairwise dipolar frequency due to the higher probability of multiple spin-flipping events. In the case where the pure NO-Cr_7Ni dipolar interaction is measured a modulation depth of 30% is obtained at the optimum T_{mix} (5 times T_1 of the fastest relaxing spin) of the experiment. An increase in modulation depths is seen as we begin to extend T_{mix} and the temperature of the experiment, up to a maximum of ~50%. Due to the selectivity of the detection pulses and the large disparity in T_{mix} from that for the optimised trace (yellow), the enhanced modulation depths may be a result of the extended T_{mix} in which the fast relaxing spin of the targeted pairwise interaction has more time to undergo stochastic spin flipping events which increase the modulation of the detection spin echo. However, studying the range of distance distributions predicted from the datasets (Figure S.5.15) shows that as the mixing time is extended at 5 K there is both an increase in the signal predicted at ca. 3 nm, corresponding to the NO-Cu(II) interaction, and a shift to lower distances of the peak centred at ca. 1.35 nm, corresponding to the NO-Cr_7Ni interaction. In particular the broadening of the peak centred at ca. 1.35 nm may be indicative of the presence of so-called ghost distances, which are a result of multi-spin interactions. Further work on the influence of multi-spin interactions in orthogonal spin models is planned. However, as the relative intensity of the two interactions seen in the distance distributions is always heavily skewed towards the presence of only one interaction, and the data measured at 3 K and 6 K are dominated by the NO-Cr_7Ni and NO-Cu(II) interactions respectively, it is a reasonable first approximation to simply scale and combine the best fitting orientation dependent simulations of the pure pairwise interactions at 3 K and 6 K in order to fit the data at 5 K and intermediary T_{mix} as presented in the main text based off the % of the T_1 value of the Cu(II) at 5 K that can be accessed (Fig. 5, a – main text). Given a fitted T_1 of 5.88 ms (Figure S.3.7) these values correspond to 17% of T_1 (best fit scaled to 10% of Cu(II) 6 K RIDME fit), 255% of T_1 (best fit scaled to 25% of Cu(II) 6 K RIDME fit), and 510% of T_1 (best fit scaled to 50% of Cu(II) 6 K RIDME fit). The values used in the scaling of variable temperature RIDME detecting on the nitroxide spin provide reasonable fits to the data. If multi-spin effects were assumed, the simulation of orientation dependent dipolar taking into account the effect of more than one spin on the detection spin echo would be required in order to accurately fit the data.

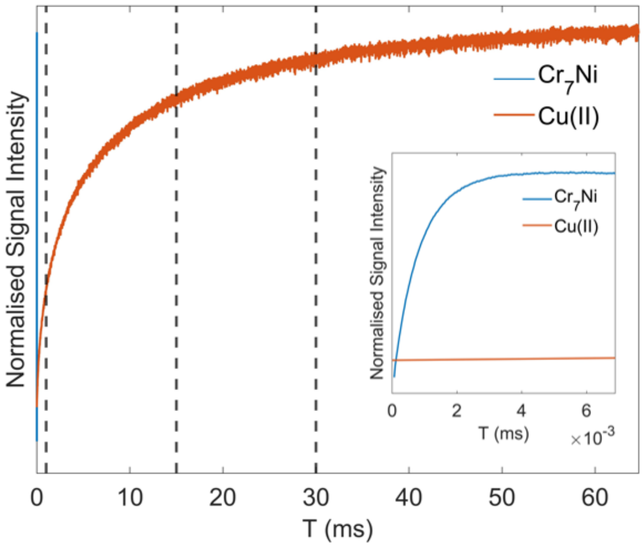

Figure S.5.16: (*Main*) T_1 experiments on Cu(II) (orange) and Cr_7Ni (blue) spins as presented in **S3**, showing T_{mix} chosen at 5 K for the "multi-spin" RIDME experiments. (*Inset*) Zoom into Cr_7Ni inversion recovery experiment showing fast T_1 relaxation compared to Cu(II).

S.6 - EPR-based Quantum Simulation of Decoherence

In order to perform a quantum simulation of the incoherent evolution of a quantum system, we consider an extended register containing additional qubits used to mimic the interaction of the system with the environment. In particular, the Cu(II) and nitroxide spins encode the "logical" qubits (L), while the Cr_7Ni ring is the ancilla (A), representing the environment.

The incoherent dynamics of L is then simulated by first performing a coherent quantum simulation on the enlarged L+A system, in which L and A are entangled, followed by fast relaxation of A. This last step is equivalent to tracing out the environment degrees of freedom, thus making the simulated dynamics of L incoherent.

Here we focus, in particular, on the simulation of pure dephasing acting on a pair of system qubits. The steps involved in the procedure are the following:

1. Preparation of the initial state of L (see below).
2. Rotation of A by a pulse resonant with a Cr_7Ni ring transition. The rotation angle controls the values of t/T_2 that we aim to simulate. This can be done with fast pulses, because of the need to rotate the Cr_7Ni ring independently from the state of the other two.
3. Generate an entangling gate between A and one (controlled-NOT) or both (controlled-controlled-NOT) L qubits. This requires long pulses to resolve the dipole-dipole interactions in order to make the Cr_7Ni excitation dependent on the state of one or both L qubits.
4. Wait for the longitudinal relaxation of the Cr_7Ni ring.

We now illustrate the preparation of the initial L state and the quantum simulation of decoherence in more detail.

A. Preparation of the initial state of L

A generic (factorised) state of the two "logical" qubits can be prepared by microwave pulses resonant with Cu(II) and/or nitroxide transitions.

In order to prepare an initial entangled state of the Cu(II) and nitroxide spin qubits (L), two-qubit gates among them are needed. The latter can be implemented by temporary excitations of the Cr_7Ni spin.[16] More specifically, as long as the Cr_7Ni ring is in the ground state the Cr_7Ni ring-Cu(II) and Cr_7Ni ring-nitroxide interactions only renormalise the external magnetic field, thus enabling single-qubit rotations of the two "logical qubits". Conversely, the excitation energy of Cr_7Ni depends on the state of the other two spin qubits and therefore it is possible to perform excitations of the ring conditioned to the states of Cu(II) and nitroxide qubit pairs and hence two-qubit gates.

In particular, we can induce a full Rabi on the Cr_7Ni ring only when both system qubits are ↓. In this way, a phase -1 is added only to the $|\downarrow\downarrow\rangle$ component of the two system qubit wavefunction, thus implementing a controlled-Z gate on L. By complementing this controlled-Z gate with rotations on L qubits, an entangled Bell state can be generated. The shorter T_1 of the ring is not a major limitation here, because it is excited only during these two-qubit gates.

B. Quantum simulation of pure dephasing on L

On a **single qubit coupled to an ancilla** to mimic the environment, we can simulate the effect of pure dephasing on a generic system state $\alpha|0\rangle + \beta|1\rangle$ as follows:

- Start with A qubit in $|0\rangle$. Hence, the L+A state becomes $(\alpha|0\rangle + \beta|1\rangle) \otimes |0\rangle$

- Rotate A by an angle ϑ, e.g. $R_y^{(E)}(\vartheta) \to (\alpha|0\rangle + \beta|1\rangle) \otimes (\cos\frac{\vartheta}{2}|0\rangle + \sin\frac{\vartheta}{2}|1\rangle)$

- Controlled-NOT where L is the control and A is the target:

$$\to \alpha\cos\frac{\vartheta}{2}|00\rangle + \beta\cos\frac{\vartheta}{2}|11\rangle + \alpha\sin\frac{\vartheta}{2}|01\rangle + \beta\sin\frac{\vartheta}{2}|10\rangle$$

- Wait for the longitudinal relaxation of A. The effect of relaxation at $T = 0$ is expressed on a single qubit by the Kraus operators $E_0 = |0\rangle\langle 0| + |1\rangle\langle 1|e^{-t/2T_1}$, $E_1 = |0\rangle\langle 1|\sqrt{1 - e^{-t/T_1}}$, which for $t \gg T_1$ become $E_0 = |0\rangle\langle 0|$ and $E_1 = |0\rangle\langle 1|$. It is worth noting that the relaxation time of Cr_7Ni ring can be further shortened if needed by exciting the ring to the S = 3/2 quartet (this excitation is allowed due to the significant difference between the g values of the Cr(III) and Ni(II) ions of the Cr_7Ni ring).

The result of the Kraus map on the system density matrix $\rho \to E_0 \rho E_0 + E_1\, \rho E_1$ is equivalent to tracing over the bath degrees of freedom, i.e. applying the transformation:

$$\rho = |\psi\rangle\langle\psi| \to \rho' = |0\rangle_E\langle 0|\rho|0\rangle_E\langle 0| + |0\rangle_E\langle 1|\rho|1\rangle_E\langle 0| = |0\rangle_E\langle 0| \otimes (\langle 0_E|\rho|0_E\rangle + \langle 1_E|\rho|1_E\rangle) \equiv |0\rangle_E\langle 0| \otimes \rho_S$$

$$\rho_S = \begin{pmatrix} |\alpha|^2 & \alpha\beta^* \cos\vartheta \\ \alpha^*\beta \cos\vartheta & |\beta|^2 \end{pmatrix}$$

which models pure dephasing by assuming $\cos\vartheta = e^{-t/T_2}$.

The above scheme can be generalised to a pair of entangled L qubits, interacting with a single A qubit. For instance, consider a Bell pair $(|00\rangle + |11\rangle)/\sqrt{2}$ of the L qubits, with A (the third qubit in the following) prepared in a generic state through an $R_y^{(E)}(\vartheta)$ rotation. The state of the **three-qubits** is then given by:

$$(|00\rangle + |11\rangle)/\sqrt{2} \otimes (\cos\frac{\vartheta}{2}|0\rangle + \sin\frac{\vartheta}{2}|1\rangle)$$

We then implement a controlled-controlled-NOT gate on A controlled by both L qubits, i.e. we perform the transformation $|110\rangle \leftrightarrow |111\rangle$. This leads to the entangled state:

$$\cos\frac{\vartheta}{2}|000\rangle + \sin\frac{\vartheta}{2}|001\rangle + \cos\frac{\vartheta}{2}|111\rangle + \sin\frac{\vartheta}{2}|110\rangle$$

Which after waiting for the longitudinal relaxation of A leads to the reduced density matrix on the system:

$$\rho_S = \frac{1}{2}(|00\rangle\langle 00| + |11\rangle\langle 11|) + \sin\vartheta\,(|00\rangle\langle 11| + |11\rangle\langle 00|)$$

which models pure dephasing by assuming $\sin\vartheta = e^{-t/T_2}$, with T_2 the relative coherence between $|00\rangle$ and $|11\rangle$.

S.7 - Spectroscopic data

UV-vis

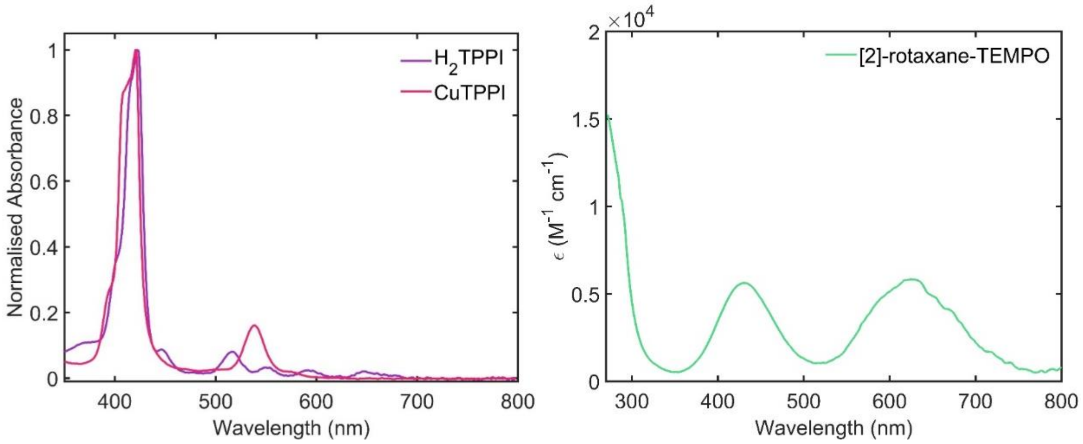

Figure S.7.1: (*Left*) UV-vis spectra of H_2TPPI **(3)** (purple), and CuTPPI **(4)** (magenta), recorded at 293 K in $CHCl_3$ at concentrations no higher than ~10^{-6} M. (*Right*) UV-vis spectra of [2]-rotaxane-TEMPO (**8**) (green) recorded at 293 K in $CHCl_3$.

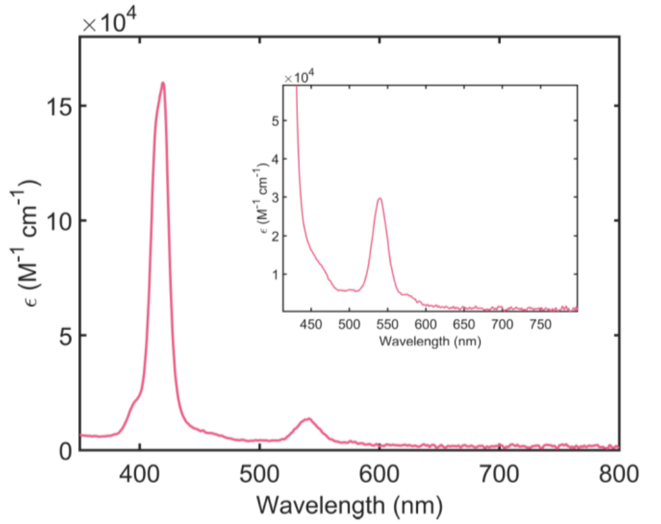

Figure S.7.2: UV-vis spectra of CuTPP-{Cr_7Ni}-TEMPO **(9)** (pink) recorded at 293 K in toluene:THF:$CHCl_3$/1:1:1. (*Inset*) Vertical zoom on the Q-band region.

^{1}H NMR

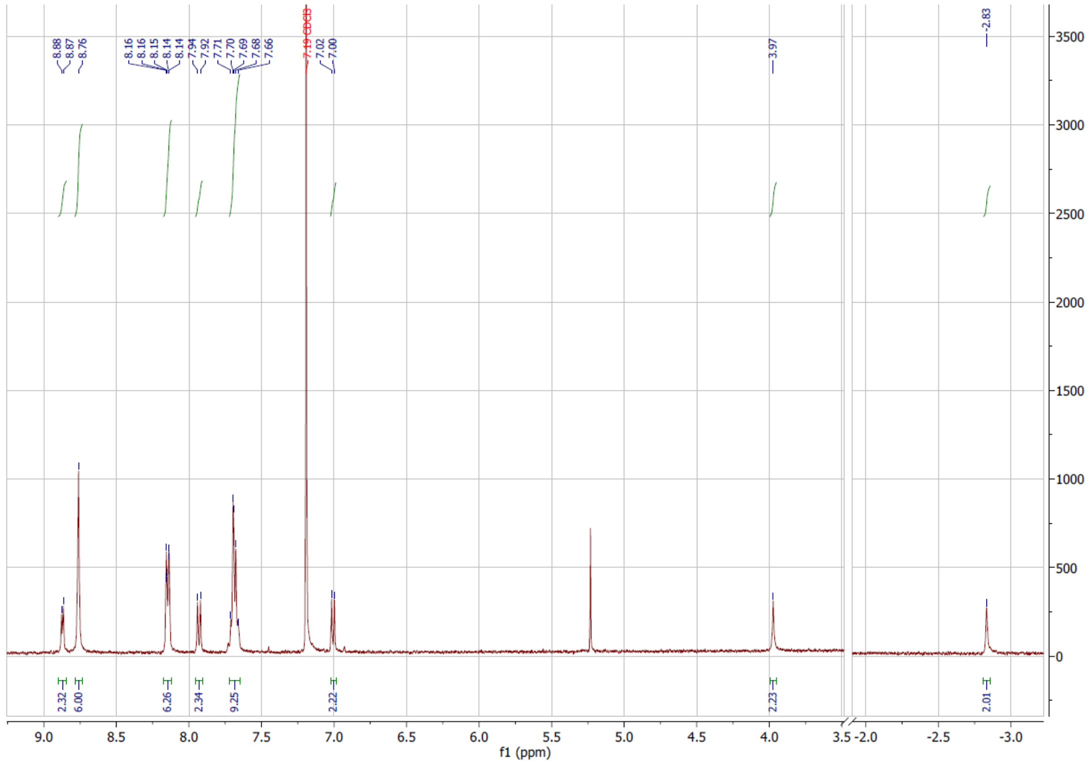

Figure S.7.3: ^{1}H NMR (400 MHz) of compound (**2**) – H_2TPPNH_2, recorded at 298 K in $CDCl_3$.

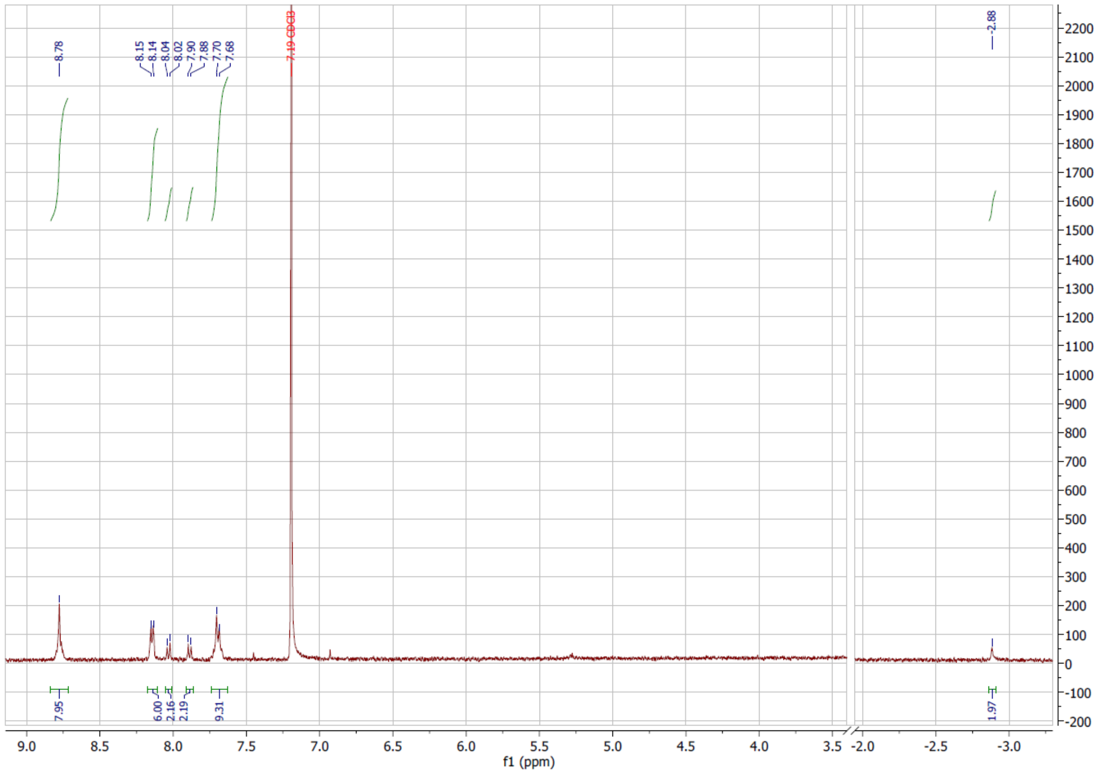

Figure S.7.4: ^{1}H NMR (400 MHz) of compound (**3**) – H_2TPPI, recorded at 298 K in $CDCl_3$.

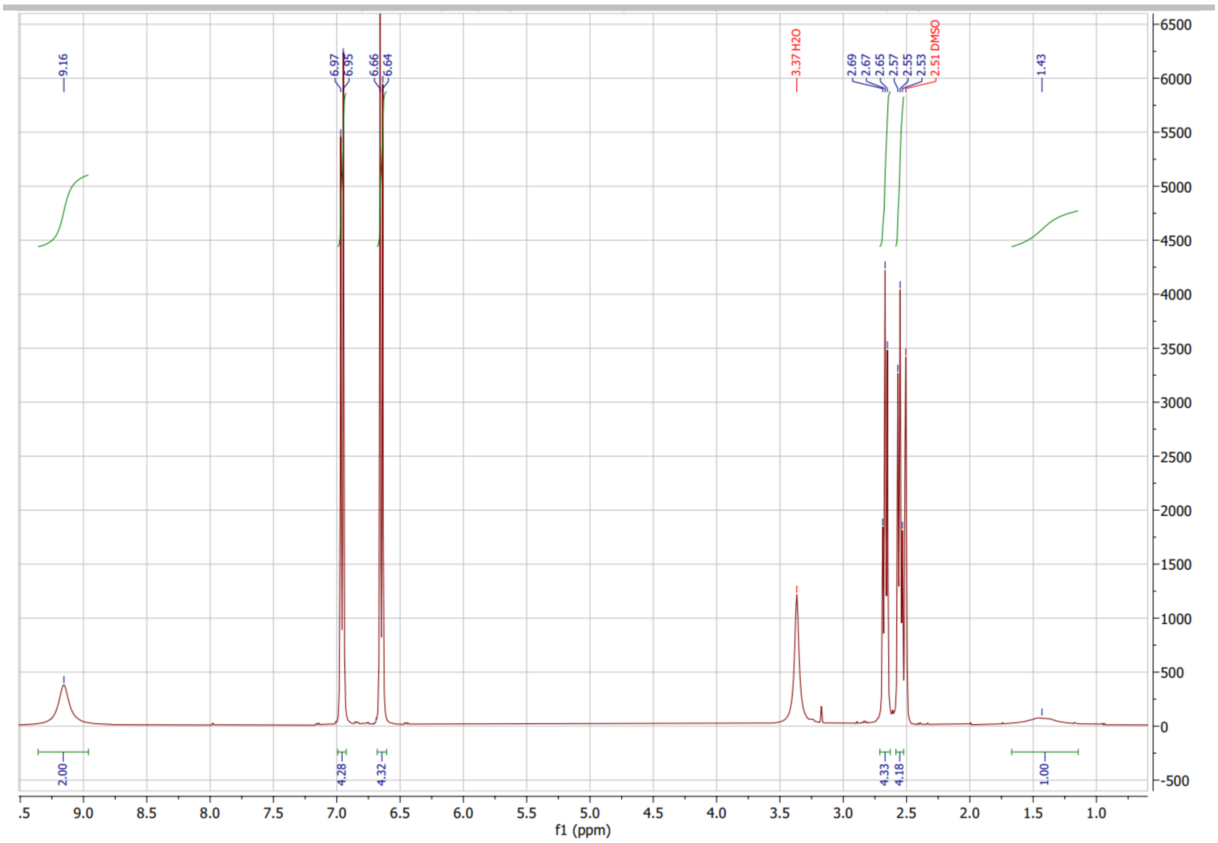

Figure S.7.5: ^{1}H NMR (400 MHz) of compound (**5**), recorded at 298 K in DMSO-d_6.

Mass Spectrometry

Thermo Exactive Plus EMR Orbitrap ASAP pos and neg

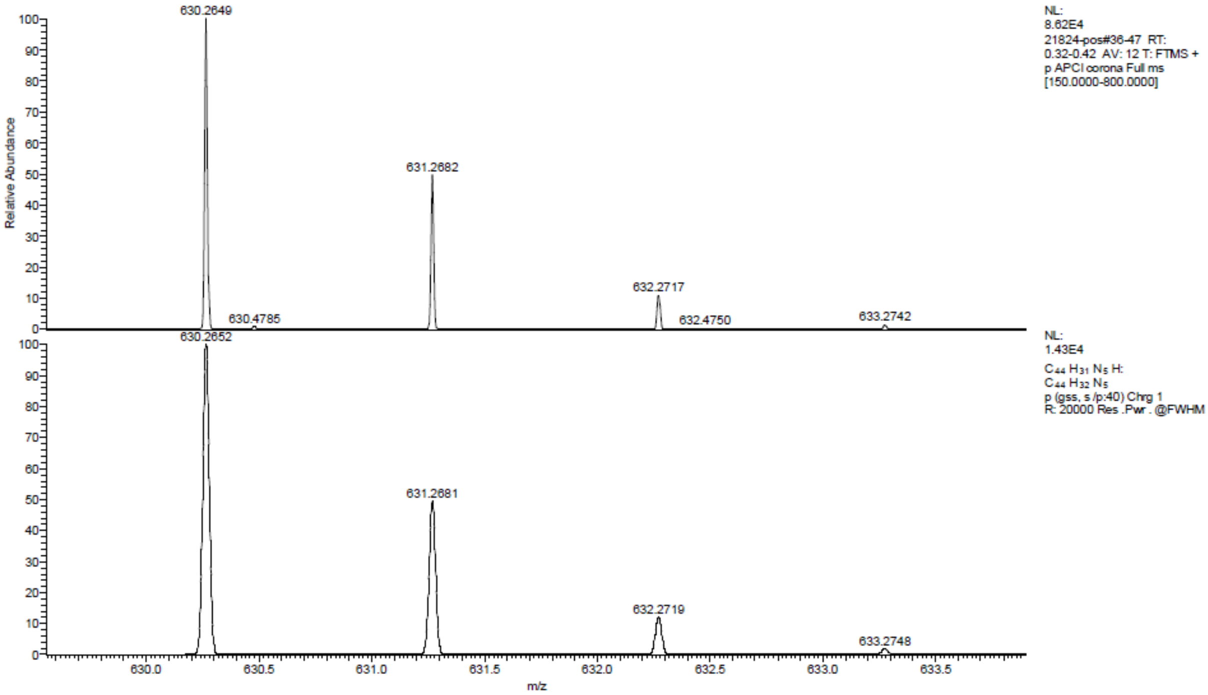

m/z	Theo. Mass	Delta (ppm)	RDB equiv.	Composition
**630.2649**	**630.2652**	**-0.51**	**31.5**	**C44 H32 N5 ←**

Figure S.7.6: Atmospheric Pressure Chemical Ionisation (APCI) high resolution accurate mass measurement of compound (**2**) – H_2TPPNH_2, recorded in DCM.

Thermo Exactive Plus EMR Orbitrap ASAP pos

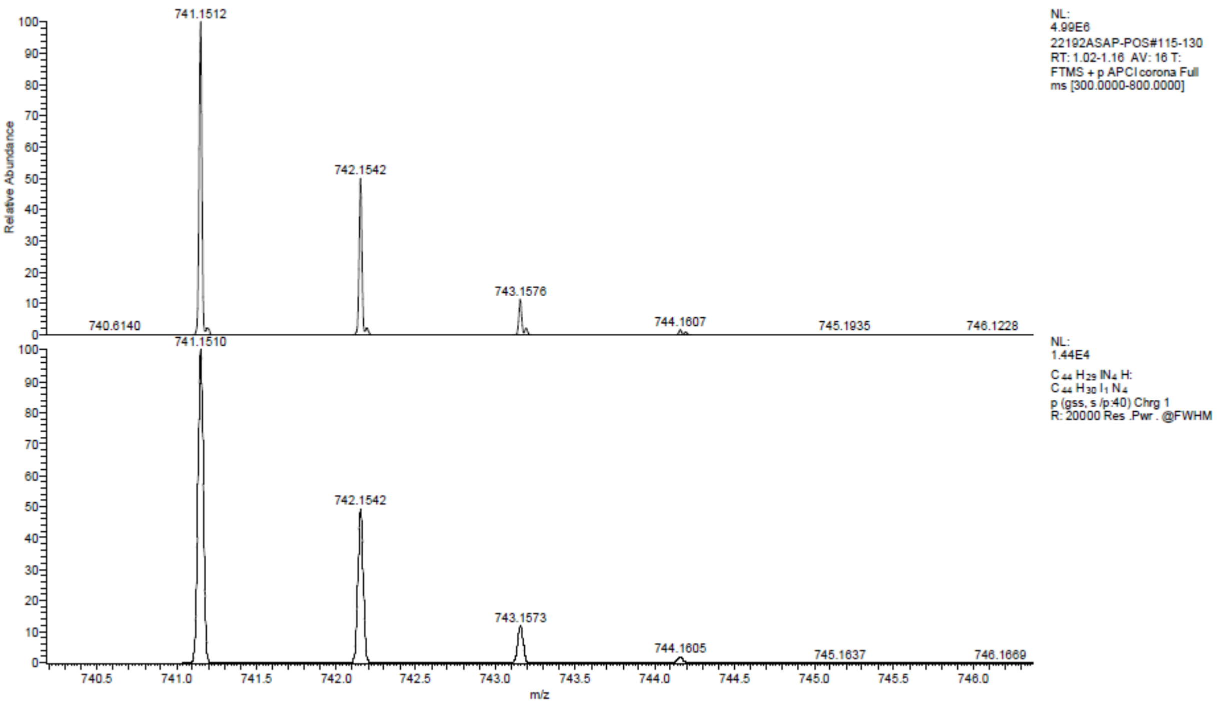

m/z	Theo. Mass	Delta (ppm)	RDB equiv.	Composition
**741.1512**	**741.1510**	**0.32**	**31.5**	**C44 H30 N4 I ←**

Figure S.7.7: Atmospheric Pressure Chemical Ionisation (APCI) high resolution accurate mass measurement of compound (**3**) – H_2TPPI, recorded in $CHCl_3$.

Thermo Exactive Plus EMR Orbitrap ASAP pos

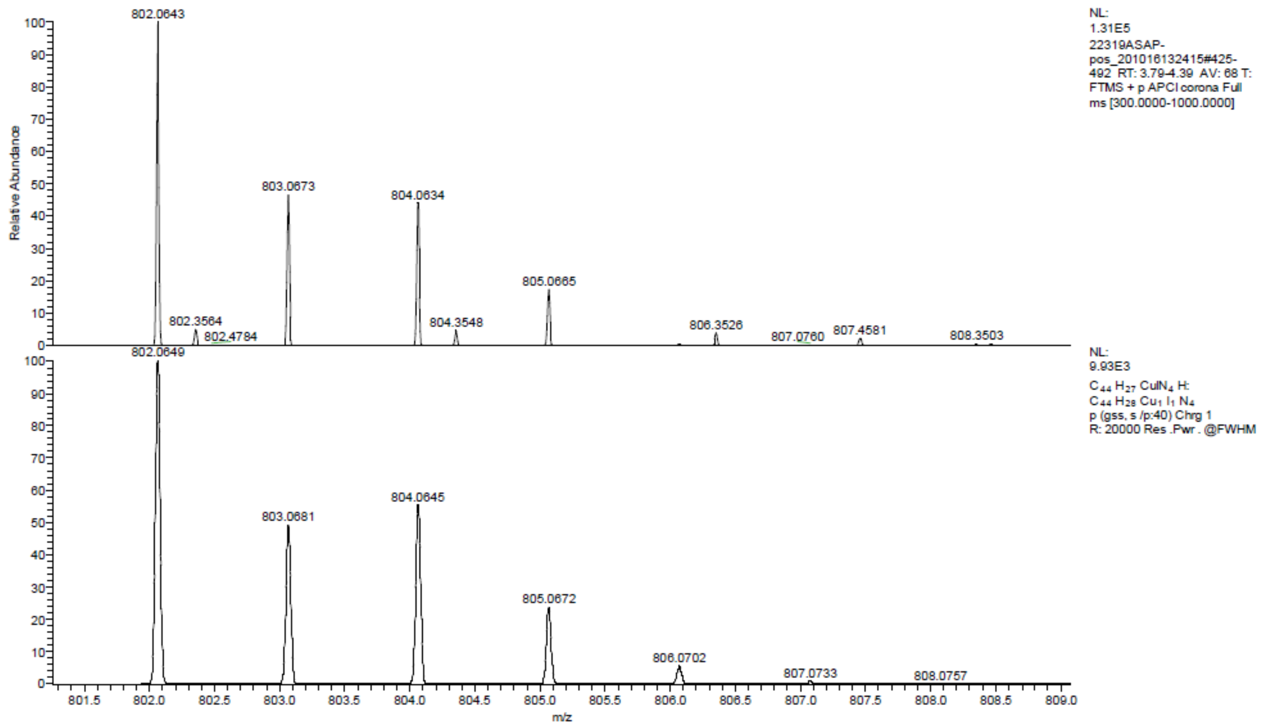

m/z	Theo. Mass	Delta (ppm)	RDB equiv.	Composition
**802.0643**	**802.0649**	**-0.77**	**32.5**	**C44 H28 N4 Cu I ← [M+H$^+$]**

Figure S.7.8: Atmospheric Pressure Chemical Ionisation (APCI) high resolution accurate mass measurement of compound (**4**) – CuTPPI, recorded in $CHCl_3$.

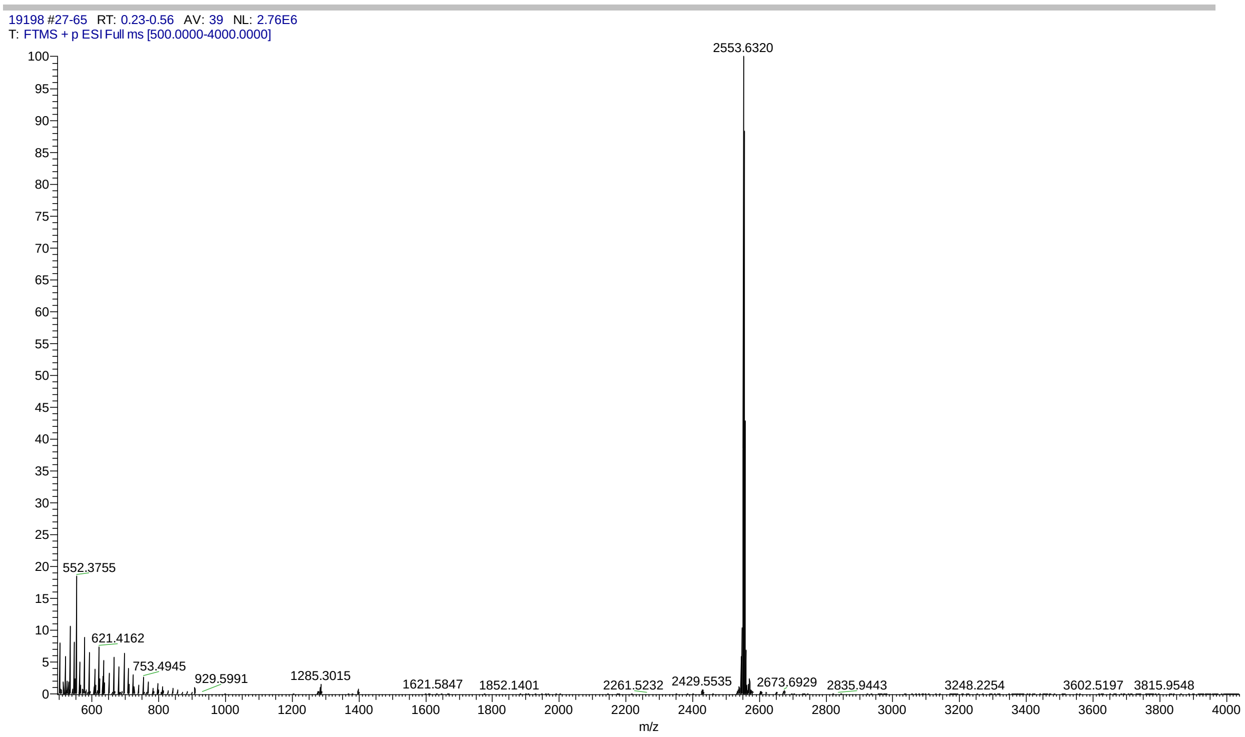

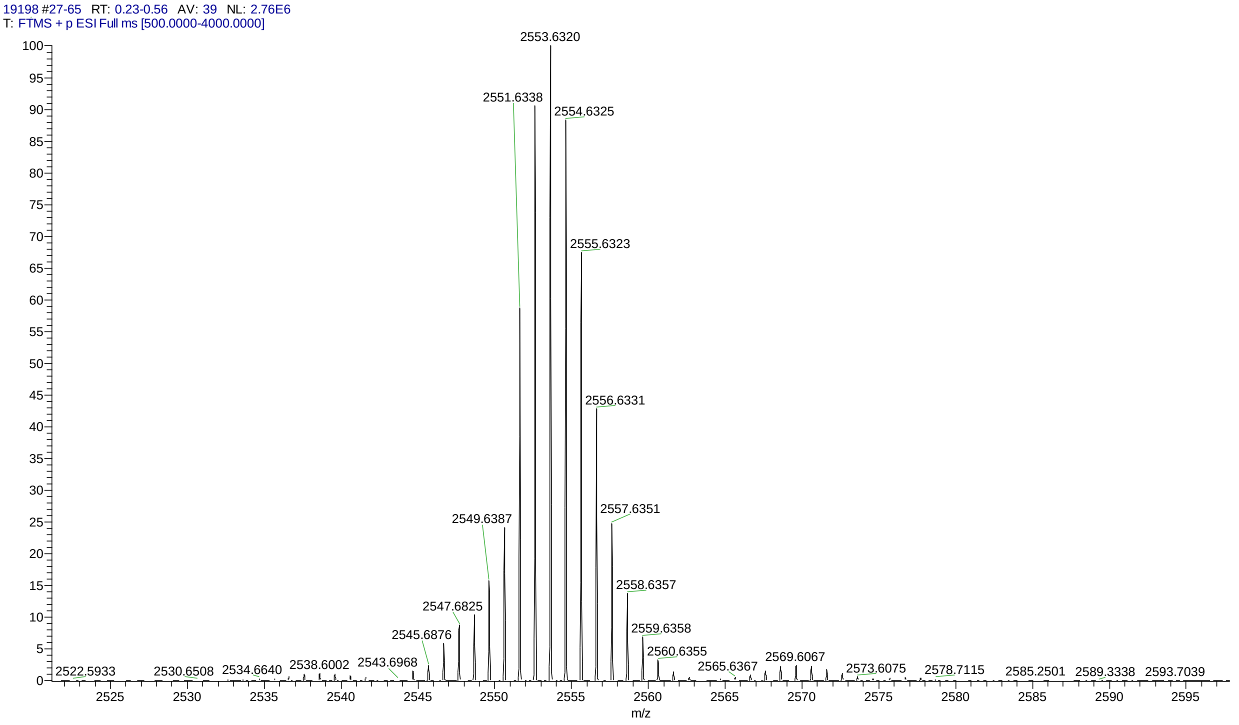

Figure S.7.9: Electrospray ionisation accurate mass measurement of [2]-rotaxane (**7**), recorded in DCM.

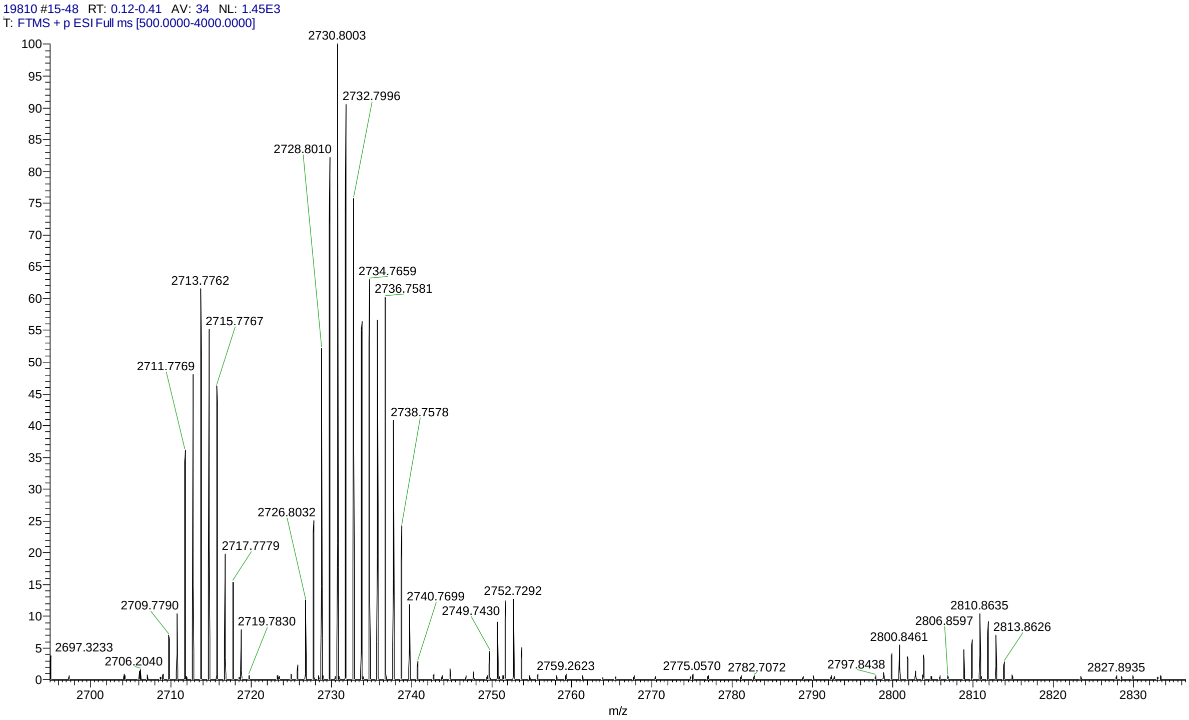

Figure S.7.10: Electrospray ionisation accurate mass measurement of [2]-rotaxane-TEMPO (**8**), recorded in DCM.

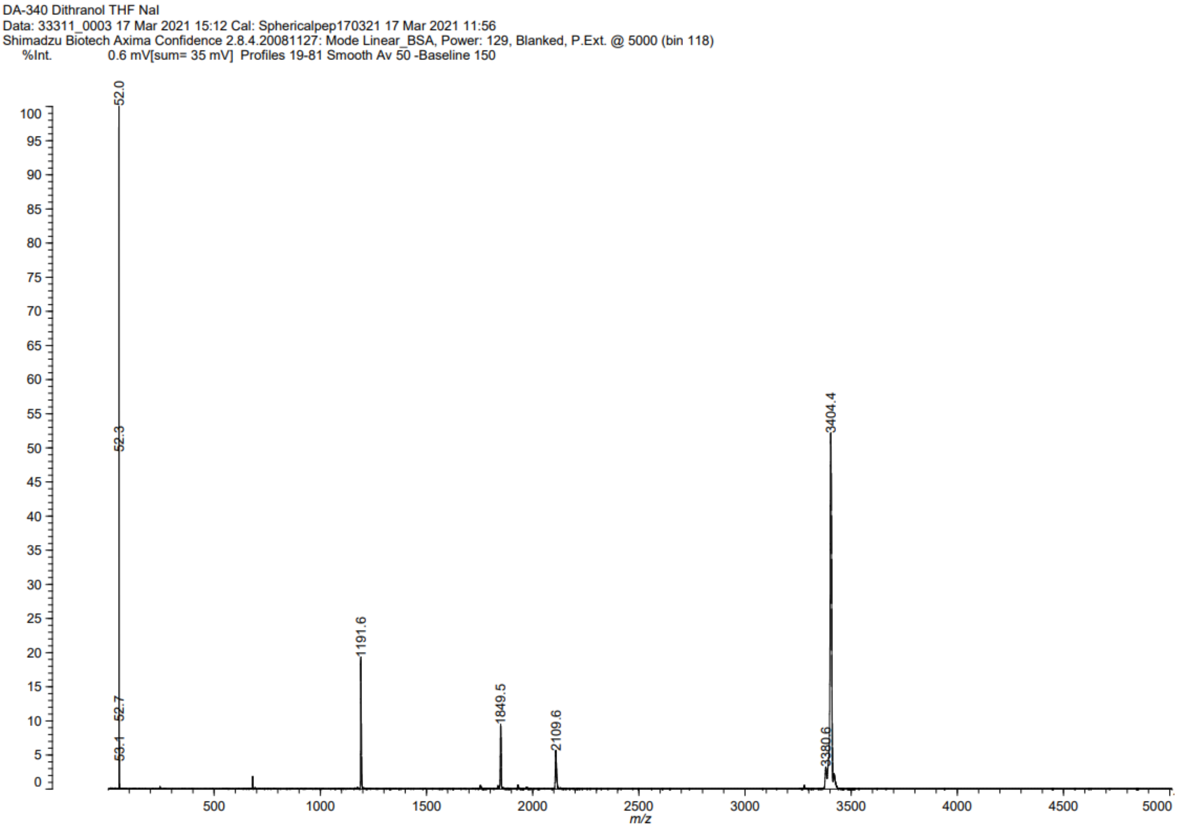

Figure S.6.11: MALDI-TOF mass measurement of CuTPP-{Cr_7Ni}-TEMPO **(9),** recorded in DCM.

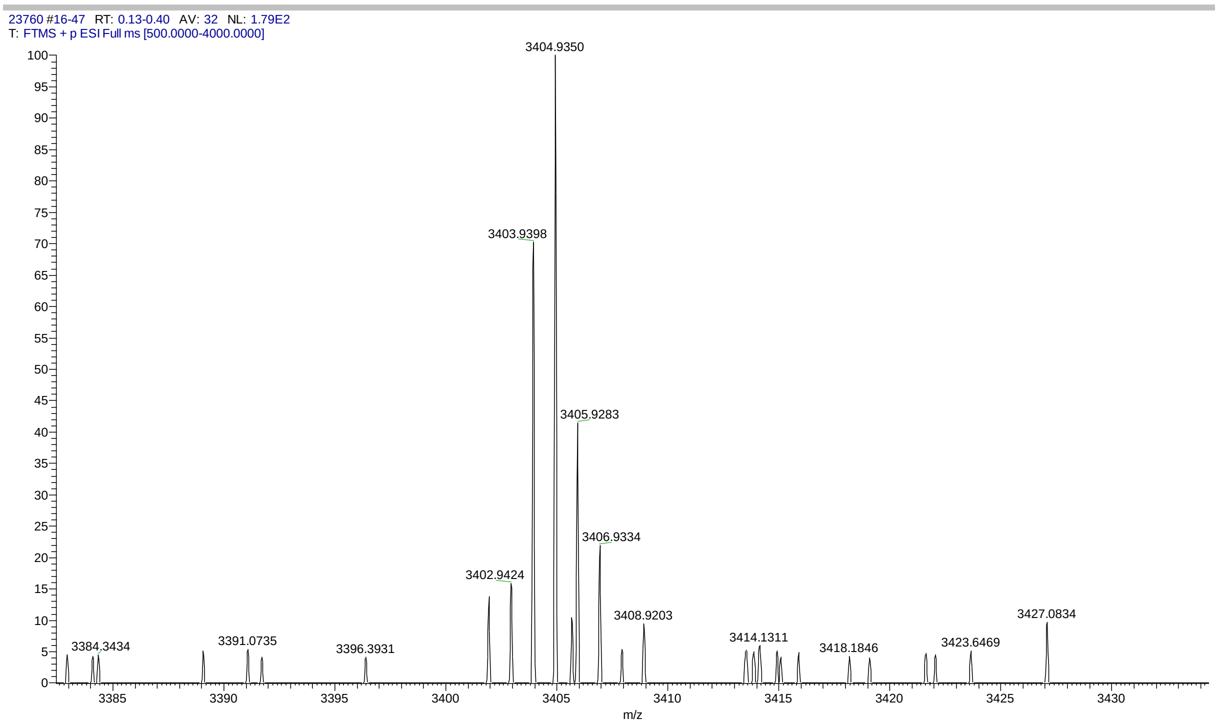

Figure S.7.12: Electrospray ionisation accurate mass measurement of CuTPP-{Cr_7Ni}-TEMPO **(9),** recorded in DCM.

References

[1] A. M. Bowen, M. W. Jones, J. E. Lovett, T. G. Gaule, M. J. McPherson, J. R. Dilworth, C. R. Timmel, J. R. Harmer, *Phys. Chem. Chem. Phys.* **2016**, *18*, 5981–5994.

[2] C. Frixa, M. F. Mahon, A. S. Thompson, M. D. Threadgill, *Tetrahedron Lett.* **2002**, *43*, 1557–1559.

[3] A. Fernandez, J. Ferrando-Soria, E. Moreno Pineda, F. Tuna, I. J. Vitorica-Yrezabal, C. Knappke, J. Ujma, C. A. Muryn, G. A. Timco, P. E. Barran, A. Ardavan, R. E. P. Winpenny, *Nat. Commun.* **2016**, *7,* 10240.

[4] R. H. Blessing, *Acta Crystallogr. Sect. A* **1995**, *51*, 33–38.

[5] H. P. O. V. Dolomanov, L. J. Bourhis, R. J. Gildea, J. A. K. Howard, *J. Appl. Cryst.* **2008**, *42*, 339–341.

[6] S. Stoll, A. Schweiger, *J. Magn. Reson.* **2006**, *178*, 42–55.

[7] J. E. Lovett, A. M. Bowen, C. R. Timmel, M. W. Jones, J. R. Dilworth, D. Caprotti, S. G. Bell, L. L. Wong, J. Harmer, *Phys. Chem. Chem. Phys.* **2009**, *11*, 6840–6848.

[8] S. J. Lockyer, S. Nawaz, A. Brookfield, A. J. Fielding, I. J. Vitorica-Yrezabal, G. A. Timco, N. A. Burton, A. M. Bowen, R. E. P. Winpenny, E. J. L. McInnes, *J. Am. Chem. Soc.* **2020**, *142*, 37, 15941–15949.

[9] E. Bordignon, *eMagRes* **2017**, *6*, 235–254.

[10] S. Richert, J. Cremers, H. L. Anderson, C. R. Timmel, *Chem. Sci.* **2016**, *7*, 6952–6960.

[11] B. E. Bode, J. Plackmeyer, T. F. Prisner, O. Schiemann, *J. Phys. Chem. A* **2008**, *112*, 5064–5073.

[12] F. Neese, *Wiley Interdiscip. Rev. Comput. Mol. Sci.* **2012**, *2*, 73–78.

[13] B. E. Bode, J. Plackmeyer, T. F. Prisner, O. Schiemann, *J. Phys. Chem. A* **2008**, *112*, 5064–5073.

[14] S. J. Lockyer, A. J. Fielding, G. F. S. Whitehead, G. A. Timco, R. E. P. Winpenny, E. J. L. McInnes, *J. Am. Chem. Soc.* **2019**, *141*, 14633–14642.

[15] S. Stoll, *eMagRes* **2017**, *6*, 23–37.

[16] J. Ferrando-Soria, E. Moreno Pineda, A. Chiesa, A. Fernandez, S. A. Magee, S. Carretta, P. Santini, I. J. Vitorica-Yrezabal, F. Tuna, G. A. Timco, E. J. L. Mcinnes, R. E. P. Winpenny, *Nat. Commun.* **2016**, *7*.
